# Docosahexaenoic acid for reading, working memory and behavior in UK children aged 7-9: A randomized controlled trial for replication (the DOLAB II study)

**DOI:** 10.1371/journal.pone.0192909

**Published:** 2018-02-20

**Authors:** Paul Montgomery, Thees F. Spreckelsen, Alice Burton, Jennifer R. Burton, Alexandra J. Richardson

**Affiliations:** 1 School of Social Policy, University of Birmingham, Birmingham, United Kingdom; 2 Centre for Evidence-based Intervention, Department of Social Policy and Intervention, University of Oxford, Oxford, United Kingdom; TNO, NETHERLANDS

## Abstract

**Background:**

Omega-3 fatty acids are central to brain-development of children. Evidence from clinical trials and systematic reviews demonstrates the potential of long-chain Omega-3 supplementation for learning and behavior. However, findings are inconclusive and in need of robust replication studies since such work is lacking.

**Objectives:**

Replication of the 2012 DOLAB 1 study findings that a dietary supplementation with the long-chain omega-3 docosahexaenoic acid (DHA) had beneficial effects on the reading, working memory, and behavior of healthy schoolchildren.

**Design:**

Parallel group, fixed-dose, randomized (minimization, 30% random element), double-blind, placebo-controlled trial (RCT).

**Setting:**

Mainstream primary schools (n = 84) from five counties in the UK in 2012–2015.

**Participants:**

Healthy children aged 7–9 underperforming in reading (<20^th^ centile). 1230 invited, 376 met study criteria.

**Intervention:**

600 mg/day DHA (from algal oil), placebo: taste/color matched corn/soybean oil; for 16 weeks.

**Main outcome measures:**

Age-standardized measures of reading, working memory, and behavior, parent-rated and as secondary outcome teacher-rated.

**Results:**

376 children were randomized. Reading, working memory, and behavior change scores showed no consistent differences between intervention and placebo group. Some behavioral subscales showed minor group differences.

**Conclusions:**

This RCT did not replicate results of the earlier DOLAB 1 study on the effectiveness of nutritional supplementation with DHA for learning and behavior. Possible reasons are discussed, particularly regarding the replication of complex interventions.

**Trial registration and protocol:**

www.controlled-trials.com (ISRCTN48803273) and protocols.io (https://dx.doi.org/10.17504/protocols.io.k8kczuw)

## Introduction

Some high-quality evidence demonstrates that increasing children’s dietary intake of the long-chain omega-3 fatty acids may improve concentration, reduce disruptive behavior and leads to better reading and spelling [1,2]. Biochemical and neuroscientific research has long demonstrated the important role of longer-chain omega-3 fatty acids–docosahexaenoic acid (DHA) and eicosapentaenoic acid (EPA)- for brain development [3,4].

Influential evidence for the potential benefits from DHA omega-3 supplementation in children stems from the DOLAB (DHA Oxford Learning and Behavior) I study [5]. This randomized, controlled trial (RCT) found that a 16-week dietary intervention with 600mg/day of algal-source DHA led to significant improvement over placebo for behavior and learning among healthy but under-performing children, aged 7–9 years, from mainstream UK schools.

Prior to DOLAB I, most studies of omega-3 supplementation for learning and behavior had involved child populations with specific developmental conditions such as attention deficit hyperactivity disorder (ADHD) [6,7], dyslexia and developmental coordination disorder (DCD) [8]. Those studies were small and their generalizability was limited by differences between the populations being studied, the treatment formulations that were used, and the outcomes assessed [9]. By contrast the DOLAB I study provided the first good evidence for the benefits of DHA omega-3 in a large sample of healthy pupils with particularly poor reading but otherwise without any behavioral or learning diagnosis.

Since the publication of the original study and the observation of heterogeneous evidence regarding learning and behaviour outcomes several trials have been published. Notably these usually focus on population with diagnosed learning or behavioural problems. A recent systematic review of polyunsaturated fatty acid (PUFA) supplementation for learning disorders found insufficient evidence of benefits in children with ADHD [10]. Notably, this review also pointed to the lack of comparable studies reporting reading as an outcome. Since then a few smaller trials found no effects for ADHD [11] however positive effects on spelling [12] and comprehensive assessments of reading ability in mainstream Scandinavian children [13,14] have been found, while other trials obtained insufficient evidence in these domains [15]. However, these studies test combinations of PUFAs with e.g. iron, and often recruited very different samples of school age children.

Three recent systematic reviews find small improvements in ADHD-type behavioral outcomes [16–18]. At the same time two Cochrane reviews [10,19] and a recent review of reviews [20] conclude that current evidence for a positive effect of polyunsaturated fatty acid supplementation for ADHD is insufficient. Interestingly, Gillies et al. [19] comment on the contradicting results to Bloch & Qawasmi [1],partly suggest that such results are due to differing combinations of parent- and teacher-rated behavior and different sets of inclusion criteria.

Of the aforementioned reviews, two included the original DOLAB I study. Whilst Tan et al.’s [10] inclusion criteria excluded DOLAB I, and Gillies et al. [19] was written prior to the publication of the original trial paper. The DOLAB I study was part of meta-analyses in Hawkey & Nigg [18] and notably in Cooper et al. [17]. For example, the latter study’s findings are strongly influenced by the results from the DOLAB 1 study, with meta-regression weights >40%.

The inconclusiveness of the current evidence on PUFA supplementation for learning and behavior in young children, particularly due to the lack of comparable studies, and the potential impact of the original DOLAB I study in past systematic reviews, highlights the need for the replication of the trial.

Importantly for the current state of evidence, Gillies et al. recommend that “*future research [should] address[…] current weaknesses in this area*, *which include small sample sizes*, *variability of selection criteria*, *variability of the type and dosage of supplementation*, *short follow-up times and other methodological weaknesses*.” [19]. This recommendation relates to ADHD studies, and should apply even more to studies in more general populations that are less common. The DOLAB II trial was a well-designed and well-powered study, with the same selection criteria, dosage and intervention period as the initial trial, thus providing the most rigorous direct test of the original findings. To the authors’ knowledge it is the first trial to assess the effects of DHA omega-3 on children’s learning and behavior in a replication RCT.

## Objectives

To replicate the beneficial effects of dietary supplementation with the long-chain omega-3 docosahexaenoic acid (DHA) on the reading, working memory, and behavior of healthy schoolchildren as originally found in the DHA-Oxford-Learning-and-Behaviour (DOLAB I) study.

## Methods

This was a parallel group, fixed-dose, randomized, double-blind placebo-controlled trial (RCT). The protocol for this trial and CONSORT checklist are available as supporting information; see **Protocol S1 File** (and at https://dx.doi.org/10.17504/protocols.io.k8kczuw) and **Checklist S2 File** and the study was registered at www.controlled-trials.com (ISRCTN48803273).

### Participants and setting

The study was open to healthy children attending mainstream UK primary schools in Oxfordshire, Northamptonshire, Buckinghamshire, Milton Keynes and Swindon who were aged 7–9 years.

#### Inclusion

Included children had to be below the 20th centile on a standardized word reading test, “The British Ability Scales” (BAS II) [21] but with no other significant special educational needs.

However, during the first wave of recruitment it was found that due to recent changes in the teaching of literacy, children’s ability to decode words had considerably improved. Consequently this study used a recalibrated version of the BAS II (New BAS II) and for comparison the new BAS 3 [22], to appropriately measure children’s reading ability. In order to meet the planned sample size, it was decided to recruit children who fell below the 20^th^ centile on *either* the recalibrated new BAS II *or* the BAS 3 word reading tests and the protocol was modified accordingly.

#### Exclusion

Children with specific medical disorders (e.g. visual or hearing impairment), or who were taking medications expected to affect behavior and learning, were excluded from the study, as were those whose first language at home was not English. Schools were also asked to exclude any children whose social/family circumstances would have made inclusion into the study inappropriate (e.g. serious illness in the family). Children who, according to their parents, ate oily fish twice or more a week or took omega-3 supplements were also excluded.

Local authorities in Oxfordshire, Buckinghamshire and Northamptonshire and the Unitary Authorities in Swindon and Milton Keynes were partners in the research, providing information on children’s performance on national attainment tests conducted at age 7 (Key Stage 1)–further details on the recruitment can be found in the supporting information. (**Recruitment S3 File**)

**Fig 1:**
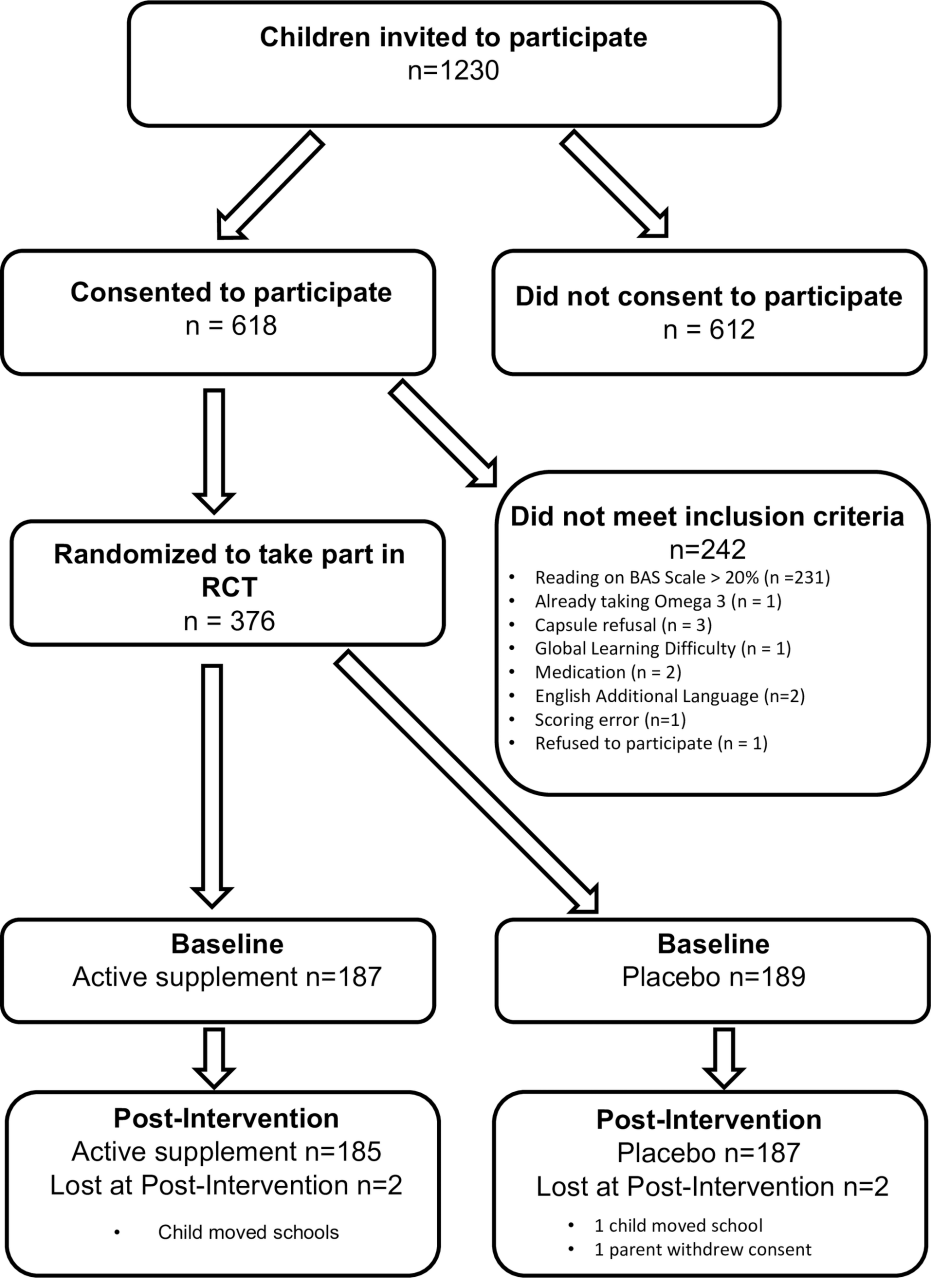
Flow of participants from invitation to randomization (CONSORT flowchart).

Having been informed about inclusion and exclusion criteria for the study, teachers at participating primary schools and academies created lists of those children whose current reading performance suggested they may benefit from inclusion to the study and on this basis, letters of invitation were sent to parents (see Fig 1).

### Ethics

Written informed consent was gained from parents, and verbal assent from the children, prior to the initial screening assessments. Ethical consent was gained from the Oxford B NHS Ethics Board, 15/10/2012, ref:12/SC/0465. Data was stored and processed anonymously.

### Intervention

Active treatment consisted of a fixed dose of 600 mg DHA (from algal oil), delivered in three 500 mg capsules per day, each providing 200 mg DHA. The placebo treatment consisted of three, taste- and color-matched 500 mg capsules per day containing corn/soybean oil. Both treatments were provided by DSM Nutritional Products, for full details see Supporting Information **Capsule Content S4 File.**

Schools were given a 16-week supply of capsules (labelled with each participating child’s name) and asked to dispense 3 capsules daily at lunch time during school terms. Likewise, parents were given a 16-week capsule supply for weekends, school holidays and at any other time pupils were absent from school.

To ensure implementation fidelity schools and parents were given detailed instructions for dispensing capsules. To increase compliance parents further received a sticker diary to record capsule consumption. To log any health issues and/or problems with capsule consumption, schools and parents received fortnightly phone calls during the course of the intervention, which were also used to encourage continued compliance.

Due to issues with the colorant and key ingredient (non-vegetarian gelatine) of the capsule shells these were changed in January 2014 and the protocol amended (for more information see **Protocol Amendment S5 File**).

### Outcomes

Primary outcomes assessed at baseline and at 16-week follow-up were:

#### a) Reading

Assessment through both the Word Reading Achievement sub-tests from the British Ability Scales (New BAS II and BAS 3 [21,22]). These are a widely used age-standardized, single word reading test, normed on UK children, and sensitive enough to show significant change over four months. Standardized scores have a mean of 100 and a standard deviation of 15, with higher scores indicating better reading.

#### b) Working memory

Assessment via the recall of digits forward and recall of digits backward sub-tests from the BAS II. Again, these measures are age standardized, but use T-scores, with a mean of 50 and a standard deviation of 10, with higher scores indicating better working memory.

#### c) Behavior

Assessment by parents using the long version of the Conners’ Rating Scale (CPRS-L) [23,24]. This is an age-standardized, highly valid and reliable instrument, measuring child behavioral problems over several domains, expressed as T-scores (mean = 50, sd = 10). Reductions in these scores represent an improvement of child behavior.

For many years these scales have been routinely used in medication trials for children with behavior problems such as ADHD; they have also been successfully used in several previous trials of fatty acid supplementation. The secondary outcome of behavior in school was measured with the teacher version of the Conners’ Rating Scale (CTRS-L)[25,26].

### Other measures

#### i) Demographic information

Information on eligibility for free school meals (FSM) was gained from local authority data and used as a proxy for Social Economic Status (SES). Local authority data were also used to report gender and age. Where such information was unavailable, parent reported data was used instead.

#### ii) Health information

At baseline information was collected from parents/guardians on each child’s current health status (including items from the side effects scale, see below). Information was also collected on possible diagnoses of ADHD and Dyslexia. Height and weight were assessed by the researchers at each child’s baseline assessment and BMI percentiles were calculated using Center for Disease Control and Prevention (CDC) guidelines [27].

#### iii) Medication

Medication information along with supplement use and fish consumption were collected from parents using a checklist. This latter information was used to confirm eligibility for the study.

#### iv) Compliance

Compliance was assessed by counting the capsules returned and by way of analyses of fingerstick blood tests pre- and post-intervention (for technical details see Supporting Information **Blood fatty acid data S6 File**). Schools and parents were also provided with a ‘calendar’ and stickers to encourage children’s compliance and to help keep track of each day’s capsule consumption. Fortnightly health-check calls also provided an opportunity for researchers to encourage compliance.

#### v) Side effects

Side effects were recorded using the Barkley Side Effects Rating Scale (SERS) [28], a commonly-used instrument assessing the frequency and severity of 17 common side effects which may occur as the result of taking medication or supplements. Each symptom is rated on a 10-point scale from absent to severe.

#### vi) Attendance

Parental consent was gained for schools to disclose each child’s attendance at school during the 16-week intervention, and this was recorded and collected at post-intervention measuring each half day’s absenteeism due to illness. Parents were also asked to report the number of days off school due to ill-health in the past school term at baseline and during the course of the intervention at the end of the study.

### Description of procedures

#### Baseline

Baseline assessments took place in schools during normal school hours in a quiet room by two trained researchers. Each child was assessed individually on reading. Only those children who met our inclusion criteria (< 20^th^ centile on the New BAS II or BAS 3), were included into the study and assessed on their working memory. Behavior questionnaires were sent out to parents with our letter of invitation whilst teachers of all those included in the study were given these questionnaires at the end of this assessment.

#### Post-intervention

Children were re-assessed at school 16 weeks post-intervention, when all primary outcome measures were repeated. On completion of the study, all participants were given a three months’ supply of the active supplement, as well as a £5 gift token.

### Sample size

Power calculations were based on change scores of reading ability from DOLAB I. In children with initial reading performance below the 20th percentile these were mean = 2.0 (SD 4.2) for the active group and mean = 0.9 (SD 3.9) for the placebo group, giving an effectsize of d = 0.28. Sample sizes were calculated with GPOWER, v3.15 [29] for a t-test. These indicated that approximately 200 participants per group would provide 80% power with an α of 5%.

### Randomization

A statistician at Sealed Envelope Ltd. independently performed the randomization with minimization via a 1:1 allocation ratio. The program’s minimization algorithm ensured balanced allocation of participants between the treatment groups for each school (to allow for any sociodemographic/school differences) and sex of the child (a potentially important factor [30]) but also included a 30% random allocation element. It was performed after eligibility was assured and was independently concealed until after the initial two-group analyses were complete. All processes are in line with CONSORT 2010 Explanation and Elaboration procedures [31] (For technical specifications see Supporting Information–**Randomisation S7 File**).

### Blinding

Investigators, participants and those assessing outcomes were all blind to treatment allocation. Post-intervention, both teachers and parents of participants were asked whether they thought their child had been allocated to Active treatment or Placebo, and these estimates were used to assess the maintenance of blinding.

### Imputation

Item-missing values in the Conner’s Rating Scales were imputed using treatment group median values, which provide some robustness against outliers, whilst not relying on an uncertain MAR assumption needed for multiple imputations. Observations lost to follow-up were also imputed using treatment group median values. Appropriate checks were made that participants with missing data did not differ significantly on any demographic variables. The methods replicated those used in DOLAB 1.

### Statistical methods

The assessment of blinding (i.e. treatment group guess) was examined using *χ*^2^–test by treatment group, whilst differences in side-effects scores were tested using Wilcoxon-rank sum tests.

Group comparisons on primary outcomes were carried out using change scores (i.e. the post-intervention score minus baseline score), in line with previous studies including DOLAB I. Main analyses were conducted using t-tests for mean differences of changes (in line with the original study) on an intention-to-treat principle (ITT): thus, all children were included according to treatment allocation, irrespective of continued participation in the trial after randomization.

For all primary outcomes, pre-planned group comparisons were carried out on the whole sample of children who were recruited into the study. Subgroup comparisons were also carried out on those children whose baseline reading scores were ≤10^th^ centile (to evaluate any possible trends related to the severity of initial reading problems).

To assess potential biases due to missingness additional per-protocol analyses were conducted on any measure with >15% missing values. Furthermore, post-hoc multivariate (OLS) regressions were undertaken to assess whether the statistically inefficient use of change-scores (in line with original paper) might affected the results. A second set of models further accounted for the minimization factors (school and gender) and assessed the consistency of the results based on the group comparisons (for details see **Supporting Information–Multivariate Analyses S1 Table).** These robustness checks are briefly discussed.

All analyses were undertaken using Stata 15.0 (StataCorp, College Station TX). Analysis syntax and an anonymised dataset are available for replication through the Open Science Framework: https://osf.io/9ynjf.

## Results

### Recruitment

Recruitment was carried out in 84 primary schools and academies in five local and unitary authorities proximate to Oxfordshire, beginning in January 2013 and finishing in March 2015. Post-intervention assessments (16 weeks after enrolment) were completed in July 2015. Of the 1230 children who were invited, 618 of their parents/guardians gave consent and their children were assessed. Of these, 376 met study inclusion criteria and were randomized. The most common reason for exclusion was that their reading exceeded the 20^th^centile (n = 231); other reasons for exclusion are described in the flowchart of participants (n = 11) detailed in Fig 1. The achieved sample size is 24 short of the planned N reflecting resource constraints.

#### Follow-up

Of the 376 children randomized, 372 were assessed again after the 16-week intervention (185 Active, 187 Placebo). Lost participants were equally balanced between groups.

#### Baseline data

The two treatment groups did not differ on any of the core demographic variables, nor on any of the primary outcome measures at baseline with the exception of working memory (Digits Forward). Demographic information is provided in Table 1. The mean age of the sample was 8 years 7 months, 62.5% were male, 84% white, and around 20% were eligible for free school meals. Baseline data on the primary outcomes are shown in Table 2. With respect to these, mean reading performance of the children randomized was 1.3 sd (20.4 points) below the normative value (score = 100), equating to a reading performance around 27 months below chronological age. Working memory scores were around 0.8 sd (8 points, digits forward) and 0.7 sd (7 points, digits backward) below population norms (score = 50). On the behavior measures, both teacher and parent ratings were all within the normative range, with the exception of the ‘cognitive problems’ sub-scale (assessing attentional and related difficulties), where these children scored 1 (parent rated, approx. 10 points) to 1.5 (teacher rated, approx. 15 points) sd above population means, as well as parent rated DSM-IV Inattentive, +1.2sd. All other behavioral measures were slightly elevated (> +0.5 sd), with the exception of ‘perfectionism’ (parent rated) and ‘oppositional’, ‘global emotional lability’, as well as ‘DSM-IV Hyperactive Impulsive’.

**Table 1:** Demographic information.

		Whole sample	(SD)	Active	(SD)	Placebo	(SD)
		N = 376		N = 187		N = 189	
***Age***	**(in months)**	105.5	10.1	105.6	10.2	105.3	10.1
***Sex***			**(%)**		**(%)**		**(%)**
	**Male**	235	62.5	120	64.2	115	63.2
	**Female**	141	37.5	67	35.8	74	40.7
***Ethnicity***							
	**White**	315	83.8	155	82.9	160	87.9
	**Mixed**	5	1.3	3	1.6	2	1.1
	**Asian**	9	2.4	5	2.7	4	2.2
	**Black**	8	2.1	5	2.7	3	1.6
	**Other**	2	0.5	2	1.1	0	0
	**Unknown**	37	9.8	17	9.1	20	11.0
***Eligibility for Free School Meals (FSM)***							
	**Not entitled**	214	56.9	109	58.3	105	57.7
	**Entitled to FSM**	78	20.7	33	17.6	45	24.7
	**Unknown**	84	22.3	45	24.1	39	21.4

**Table 2:** Primary outcomes at baseline, mean (sd).

	Whole Sample (n = 376)	(SD)	N	Active (n = 187)	(SD)	N	Placebo (n = 189)	(SD)	N
READING*									
**Word Reading–Standard Score (sd) §**	79.6	(6.5)	376	80	(6.4)	187	79.2	(6.5)	189
***Reading age*, *months (sd)***	78.4	(8)	376	79.1	(7.8)	187	77.7	(8.1)	189
Working memory*	42	(8.6)	376	42.9	(8.6)	187	41.1	(8.5)	189
**Digits Forward–T-Scores**^**††**^ **(sd)**	42	(8.6)	376	42.9	(8.6)	187	41.1	(8.5)	189
**Digits Backward–T-Scores**^**††**^ **(sd)**	42.8	(8.4)	374	43.2	(8.1)	187	42.5	(8.8)	187
***BEHAVIOR*:**									
***Parent rated‡***									
**Sub-scales (T-scores)**^**†**^									
**Oppositional**	55.5	(12.6)	303	55.3	(12.5)	147	55.7	(12.7)	156
**Cognitive Problems**	60.5	(11.6)	307	60.6	(11.7)	150	60.5	(11.5)	157
**Hyperactivity**	57.1	(13.1)	306	57.7	(12.6)	148	56.6	(13.5)	158
**Anxiety**	52.7	(11.8)	307	51.6	(11.1)	148	53.8	(12.4)	159
**Perfectionism**	50.2	(11.4)	307	50.8	(11.7)	148	49.7	(11.1)	159
**Social Problems**	55.9	(13.3)	307	55.2	(13.2)	148	56.4	(13.4)	159
**Psychosomatic**	54.9	(13.3)	307	55	(14)	148	54.8	(12.7)	159
**Global scales (T-scores)** ^**††**^									
**ADHD Index**	58.4	(11.7)	305	58.5	(11.7)	146	58.3	(11.8)	159
**Global Restless-Impulsive**	57.5	(12.7)	302	58	(12.8)	144	57.1	(12.6)	158
**Global Emotional Lability**	55.2	(12)	303	54.9	(12)	146	55.5	(12)	157
**Global Index Total**	57.4	(12.5)	302	57.6	(12.6)	144	57.2	(12.5)	158
**DSM-IV Inattentive**	57.5	(11.8)	305	57.5	(11.9)	148	57.6	(11.8)	157
**DSM-IV Hyperactive-Impulsive**	58.1	(13.1)	303	58.7	(12.8)	145	57.6	(13.4)	158
**DSM-IV Total**	58.1	(12.6)	306	58.5	(12.6)	148	57.8	(12.6)	158
***Teacher rated‡‡***									
**Sub-scales (T-scores)** ^**††**^									
**Oppositional**	54.7	(12.2)	269	55.4	(12.9)	136	54	(11.5)	133
**Cognitive Problems**	66.8	(8.4)	266	66.7	(8.1)	134	67	(8.6)	132
**Hyperactivity**	55.2	(11.2)	268	55.8	(11.2)	135	54.6	(11.1)	133
**Anxiety**	59.2	(13)	269	59	(12.9)	136	59.4	(13.1)	133
**Perfectionism**	47.7	(7.5)	267	48	(8.4)	135	47.4	(6.5)	132
**Social Problems**	55	(12)	268	54.8	(11.5)	135	55.3	(12.6)	133
**Global scales (T-scores)**^**††**^									
**ADHD Index**	59.3	(10.8)	267	59.7	(10.5)	135	58.9	(11.2)	132
**Global Restless-Impulsive**	58.7	(11)	269	59.4	(10.5)	136	57.9	(11.4)	133
**Global Emotional Lability**	54.6	(12.4)	267	55.5	(13.1)	135	53.6	(11.7)	132
**Global Index Total**	58.2	(11.5)	268	59.1	(11.2)	135	57.4	(11.8)	133
**DSM-IV Inattentive**	62.4	(9.8)	267	62.3	(9.6)	135	62.5	(10)	132
**DSM-IV Hyperactive-Impulsive**	54	(11.4)	267	54.6	(11.3)	135	53.4	(11.5)	132
**DSM-IV Total**	59.5	(10.1)	267	59.7	(10)	135	59.2	(10.4)	132

*Obtained from the British Ability Scales II.

‡Obtained from Conners' Parent Rating Scale (CPRS-P).

‡‡Obtained from Conners' Teacher Rating Scale (CTRS-L).

^**†**^Standard Scores have a mean of 100 (sd = 15).

^**††**^Standard Scores have a mean of 50 (sd = 10).

#### Did blinding work?

Parent and teacher estimates of group allocation at post-intervention were used to assess the maintenance of blinding. Group comparisons carried out on these estimates showed there were no significant differences between groups (parents’ estimate: chi2(df) = 1.327(2); teachers’ estimate: chi2(df) = 0.818(2), as shown in Table 3.

**Table 3:** Maintenance of blinding for parents and teachers, n (%) returned questionnaires.

	Actual Treatment Allocation			
	Parents		Teachers	
**"Guessed" treatment allocation**	**Active (n = 99)**	**Placebo (n = 110)**	**Active (n = 124)**	**Placebo (n = 122)**
**Active**	39 (39.4%)	46 (41.8%)	49 (39.5%)	43 (35.2%)
**Placebo**	58 (58.5%)	61 (55.5%)	69 (55.7%)	71 (58.2%)
**Don’t know**	2 (2.1%)	3 (2.7%)	6 (4.8%)	8 (6.6%)
**Missing (questionnaires not returned)**	88	79	63	67
**Test (Allocation vs. Guess)**	**Parents: chi2 (df)**	**Pvalue**	**Teachers: chi2 (df)**	**Pvalue**
	1.327 (2)	0.723	0.818 (2)	0.845

#### Numbers analysed

Intention-to-treat analyses were carried out on the whole sample randomized (n = 376). Analyses were also carried out on the pre-planned sub-group defined by baseline reading of below the 10th centile (n = 213) in line with the protocol. Behavior ratings were the only measures with >15% of the data missing (change scores n = 196 for teachers (52%), and n = 187 for parents (50%)), so additional per-protocol analyses were conducted on these measures.

### Outcomes

#### a) Reading

**Fig 2:**
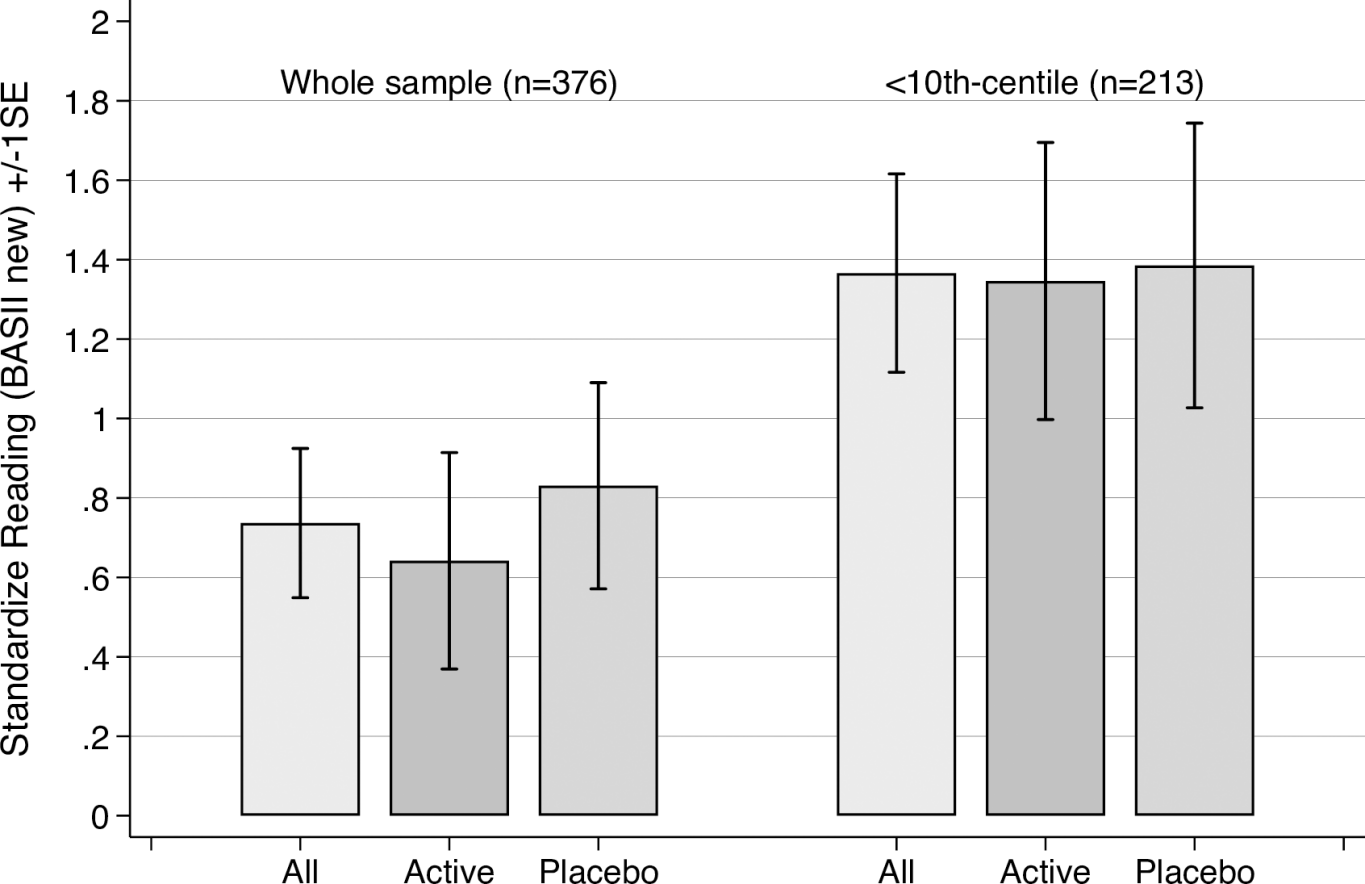
Change in standardized reading scores^†^ by treatment group for all children randomised and for sub-groups with initial reading of ≤10th centiles (± 1 SE). **Note**: ^†^Obtained from the British Ability Scales II new calibration. Standardized scores have a mean of 100 (sd = 15).

Standardized reading score data are shown in Table 4, and changes on this measure, which were the primary outcome, are illustrated in Fig 2. The same data expressed as ‘reading ages’ are shown in Table 5.

**Table 4:** Standardized* reading measures^†^, means (sd).

	Baseline				Post-Intervention				Change Scores			
	Active	Placebo	p	t	Active	Placebo	p	t	Active	Placebo	p	t
**All randomized (n = 376)**	80.0 (6.4)	79.2. (6.5)	0.240	1.177	80.6 (6.7)	80.0 (6.5)	0.385	0.870	.64 (3.7)	.83 (3.6)	0.616	-0.502
**Reading ≤ 10th Centile (n = 213)**	75.4 (4.5)	74.8 (4.8)	0.332	0.972	76.7 (5.5)	76.2 (5.1)	0.432	0.786	1.4 (3.6)	1.4 (3.7)	0.938	-0.078

* Obtained from the British Ability Scales II.

^†^Standardized scores have a mean of 100 (sd = 15).

**Table 5:** Standardized* reading age (in months), means (sd).

	Baseline				Post-Intervention				Change Scores			
	Active	Placebo	p	t	Active	Placebo	p	t	Active	Placebo	p	t
**All randomized (n = 376)**	79.1 (7.9)	77.7 (8.2)	0.091	1.695	82.1 (9.1)	81.4 (8.1)	0.437	0.778	3.1 (4.4)	3.7 (4.9)	0.143	-1.467
**Reading ≤ 10th Centile (n = 213)**	75.6 (5.7)	74.5 (5.9)	0.172	1.369	78.4 (6)	78 (6.5)	0.672	0.424	2.7 (3.5)	3.5 (4.3)	0.179	-1.348

* Obtained from the British Ability Scales II.

After the 16-week treatment period no statistically significant differences were found between treatment groups post-intervention.

The whole group randomized (n = 376), showed no statistically significant reading gain differences by treatment group above those that would be expected over this time period (Active change(sd) = 0.64(3.7); placebo change(sd) 0.83(3.6), p(t) = 0.616(-0.502). This is further illustrated by the fact that children’s reading age increased by 3.1 months (active) and 3.7 months (placebo) respectively over the 4 months of the intervention (Table 5).

The same result was obtained for the pre-planned sub-group whose baseline reading was at or below the 10^th^ centile (n = 213). In this subgroup, no statistically significant group differences in change-scores were observed (Active change(sd) = 1.4(3.6); Placebo change(sd) = 1.4(3.7); p(t) = 0.938(-0.078)).

Finally, Table 6 reports the group mean differences and 95% confidence intervals, in the main sample the differences is -0.594 (95% CI: -1.937, 0.749) in the subgroup -0.576 (-2.019, 0.867) points on the BASII reading scores. This further shows that the treatment group differences are not substantially meaningful.

**Table 6:** Post-intervention mean differences (95% CI) for standardized^†^ reading and reading age (in months) *.

	All randomized (n = 376)			Reading ≤ 10th Centile (n = 213):		
	Mean Difference	95%	CI	Mean Difference	95%	CI
Reading	-0.594	-1.937	0.749	-0.576	-2.019	0.867
Reading Age	-0.689	-2.431	1.052	-0.365	-2.06	1.331

* Obtained from the British Ability Scales II.

^†^Standardized scores have a mean of 100 (sd = 15).

#### b) Working memory

At baseline (Table 7), *digits forward* scores differed statistically significantly between the treatment groups in both the whole sample and the subgroup of children in the <10-centile of the (normative BAS) reading distribution. At post-intervention, group means differed significantly for digits forward (Whole sample: active mean(sd) = 43.8, (9.1), placebo mean(sd) = 42.0(9.3), p(t) = 0.059(1.982); 10^th^ centile subgroup: Active mean(sd) = 43.7(8.1), placebo mean (sd) = 41.9(9.0), p(t) = 0.047(1.994)). In line with these differences the change scores are both small and not statistically significant for digits forward (active change(sd) = 0.91, (7.7), placebo change(sd) = 1.72(7.9), p(t) = 0.826(-0.22)). Table 8 reports the group mean differences and 95% confidence intervals, in the main sample the differences is -1.797 (95% CI: -3.665, 0.071) in the subgroup -0.576 (95% CI; -5.304, -0.031) points, neither is close to the 10-point, one standard deviation measure indicating a clinically relevant difference.

**Table 7:** Standardized* working memory (recall of digits forward)^†^, means (sd).

	Baseline T Score (sd)				Post-Intervention T Score (sd)				Change Scores, T Score (sd)			
	Active	Placebo	p	t	Active	Placebo	p	t	Active	Placebo	p	t
**All randomized (n = 376)**	42.9 (8.6)	41.1 (8.5)	0.048	1.980	43.8 (9.1)	42.0 (9.3)	0.059	1.892	.95 (7.4)	.91 (7.7)	0.957	0.054
**Reading ≤ 10th Centile (n = 213)**	42.9 (8.8)	40.0 (8.3)	0.014	2.475	44.4 (9.8)	41.7 (7.8)	0.047	1.994	1.48 (7.7)	1.72 (7.9)	0.826	-0.220

* Obtained from the British Ability Scales II.

^†^Standardized scores have a mean of 50 (sd = 10).

**Table 8:** Post-intervention mean differences (95% CI) for working memory (recall of digits forward and backward) *^†^.

	All randomized (n = 376)			Reading ≤ 10th Centile (n = 213):		
	Mean Difference	95%	CI	Mean Difference	95%	CI
Digits Forward	-1.797	-3.665	0.071	-2.667	-5.304	-0.031
Digits Backward	-1.774	-3.503	-0.045	-3.061	-5.597	-0.526

* Obtained from the British Ability Scales II.

^†^Standardized scores have a mean of 50 (sd = 10).

*Digits Backwards* (Table 9) only differed statistically significantly at post-intervention (Whole sample: Active mean(sd) = 43.7(8.1), placebo mean(sd) = 41.9(9.0), p(t) = 0.044(2.018); and for the 10% Subgroup: Active mean(sd) = 43.5(9.3), placebo mean(sd) = 40.5(9.5), p(t) = 0.018(2.38)). Again, we find the change scores for digits backwards (active change(sd) = -0.4, (9.8), placebo change(sd) = -0.4(9.8), p(t) = 0.356(0.925)), do not differ in a statistically significant way.

**Table 9:** Standardized* working memory (recall of digits backward)^†^, means (sd).

	Baseline T Score (sd)				Post-Intervention T Score (sd)				Change Scores, T Score (sd)			
	Active	Placebo	p	t	Active	Placebo	p	t	Active	Placebo	p	t
**All randomized (n = 376)**	43.2 (8.1)	42.5 (8.8)	0.427	0.795	43.7 (8.1)	41.9 (9.0)	0.044	2.018	0.4 (9.3)	-0.4 (9.8)	0.356	0.925
**Reading ≤ 10th Centile (n = 213)**	42.3 (7.8)	41.9 (9.8)	0.742	0.330	43.5 (9.3)	40.5 (9.5)	0.018	2.380	1.1 (10.1)	-1.3 (10.7)	0.075	1.788

* Obtained from the British Ability Scales II.

^†^Standardized scores have a mean of 50 (sd = 10).

The group mean differences (Table 8) for digits backwards are, in the main sample -1.774 (95% CI: -3.503, -0.045) and in the subgroup -3.061 (95% CI; -5.597–0.526) points.

#### c) Behavior

Across both treatment groups, behavior ratings from parents showed small changes (ranging from -1 to -3.8 points) over the 16-week treatment period, as shown in Table 10 (ITT) and Table 11 (per-protocol). These reductions of behavioral problems at post-intervention occur across *both* treatment groups, and no statistically significant group differences are found. Table 12 further highlights this point due to the small group mean differences and corresponding 95% confidence intervals including zero.

**Table 10:** Standardized* behavior measures—Parent rated^†^ (ITT), means (sd).

	Baseline						Post-intervention						Change scores					
	Active (n = 187)	sd	Placebo (n = 189	sd	P	t	Active (n = 187)	sd	Placebo (n = 189	sd	P	t	Active (n = 187)	sd	Placebo (n = 189	sd	P	t
**Oppositional**	54.6	(11.1)	55.4	(11.5)	0.487	-0.696	53.6	(8.8)	53.3	(8.7)	0.782	0.276	-1.0	(9.4)	-2.1	(10.5)	0.302	1.034
**Cognitive Problems**	60.3	(10.5)	60.4	(10.5)	0.874	-0.159	59.2	(8.3)	57.5	(8.2)	0.059	1.895	-1.1	(9.4)	-2.9	(9.5)	0.068	1.833
**Hyperactivity**	57.2	(11.3)	55.9	(12.5)	0.262	1.123	54.3	(8.9)	53.1	(9.2)	0.195	1.298	-2.9	(10.1)	-2.7	(9.7)	0.872	-0.161
**Anxiety**	50.9	(10.)	53.2	(11.4)	0.036	-2.108	49.9	(7.9)	49.4	(7.1)	0.526	0.634	-1.0	(7.9)	-3.8	(9.6)	0.002	3.123
**Perfectionism**	49.9	(10.6)	49.0	(10.3)	0.358	0.92	47.6	(7.1)	47.4	(8.)	0.773	0.288	-2.3	(9.6)	-1.5	(9.)	0.423	-0.802
**Social Problems**	54.1	(12.)	55.6	(12.5)	0.261	-1.125	52.6	(9.3)	53.1	(8.9)	0.645	-0.461	-1.5	(10.3)	-2.5	(11.3)	0.377	0.884
**Psychosomatic**	53.5	(12.7)	54.2	(11.7)	0.604	-0.52	51.3	(11.)	51.4	(9.8)	0.928	-0.09	-2.2	(14.1)	-2.8	(12.2)	0.681	0.412
**ADHD Index**	58.1	(10.4)	58.1	(10.8)	0.949	0.064	56.3	(8.2)	55.3	(8.2)	0.242	1.172	-1.9	(8.1)	-2.8	(9.6)	0.318	0.999
**Global Restless-Impulsive**	57.3	(11.3)	56.8	(11.5)	0.658	0.442	55.0	(8.4)	53.7	(8.5)	0.145	1.461	-2.3	(9.5)	-3.1	(9.9)	0.455	0.747
**Global Emotional Lability**	54.3	(10.7)	55.2	(10.9)	0.409	-0.827	53.4	(9.5)	52.0	(8.3)	0.119	1.562	-0.9	(9.6)	-3.2	(9.8)	0.019	2.357
**Global Index Total**	57.6	(12.6)	57.2	(12.5)	0.76	0.306	56.0	11.7	54.5	(10.5)	0.334	0.969	-1.7	(8.4)	-3.2	7.9	0.22	1.231
**DSM-IV Inattention**	57.5	(11.9)	57.6	(11.8)	0.926	-0.093	57.3	11.2	55.1	(12.)	0.189	1.318	-1.7	(8.)	-2.9	10.5	0.368	0.902
**DSM-IV Hyperactive-Impulsive**	58.7	(12.8)	57.6	(13.4)	0.468	0.726	56.5	11.6	55.6	(11.6)	0.604	0.519	-2.8	(8.1)	-2.2	8.1	0.615	-0.504
**DSM-IV Total ADHD**	58.5	(12.6)	57.8	(12.6)	0.588	0.543	57.0	11.5	55.7	(10.9)	0.382	0.877	-2.6	(8.)	-2.6	8.5	0.99	0.013

*T-scores have a mean of 50 (sd = 10).

^†^Behaviour measures are derived from the Conners’ Parent Rating Scale (CPRS).

**Table 11:** Standardized* behavior measures—Parent rated^†^ (per-protocol), means (sd).

	Baseline								Post-intervention								Change scores							
	Active	sd	N	Placebo	sd	N	P	t	Active	sd	N	Placebo	sd	N	P	t	Active	sd	N (listwise)	Placebo	sd	N (listwise)	P	t
**Oppositional**	55.3	(12.5)	147	55.7	(12.7)	156	0.779	-0.281	54.9	11.8	101	54.2	(11.3)	111	0.68	0.414	-0.8	(9.3)	92	-1.1	9.9	95	0.789	0.268
**Cognitive Problems**	60.6	(11.7)	150	60.5	(11.5)	157	0.969	0.039	60.1	11.2	101	57.9	(10.8)	111	0.143	1.471	-2.1	(8.4)	92	-3.2	9.7	96	0.429	0.793
**Hyperactivity**	57.7	(12.6)	148	56.6	(13.5)	158	0.4735	0.718	55.5	12.1	101	54.6	(11.7)	111	0.602	0.523	-2.8	(9.7)	92	-2.1	8.0	97	0.616	-0.502
**Anxiety**	51.6	(11.1)	148	53.8	(12.4)	159	0.106	-1.621	51.5	10.5	100	50.4	(9.2)	111	0.398	0.847	-0.8	(7.3)	91	-4.1	9.2	98	**0.007**	2.717
**Perfectionism**	50.8	(11.7)	148	49.7	(11.1)	159	0.377	0.885	49.1	9.4	100	49.1	(10.1)	111	0.971	-0.036	-0.7	(10.3)	91	-0.7	8.0	98	0.957	-0.055
**Social Problems**	55.2	(13.2)	148	56.4	(13.4)	159	0.437	-0.778	54.9	12.3	100	55.2	(11.2)	111	0.854	-0.185	-0.5	(8.9)	91	-2.2	11.8	98	0.285	1.073
**Psychosomatic**	55.0	(14.)	148	54.8	(12.7)	159	0.895	0.132	54.2	14.5	100	53.8	(12.2)	111	0.831	0.214	-0.9	(15.9)	91	-2.0	12.6	98	0.598	0.528
**ADHD Index**	58.5	(11.7)	146	58.3	(11.8)	159	0.889	0.140	57.3	11.0	101	56.2	(10.6)	110	0.444	0.767	-2.3	(7.1)	92	-2.9	9.1	97	0.64	0.469
**Global Restless-Impulsive**	58.0	(12.8)	144	57.1	(12.6)	158	0.558	0.587	55.8	11.4	100	54.9	(10.9)	111	0.55	0.599	-2.4	(8.)	91	-2.9	8.2	97	0.677	0.417
**Global Emotional Lability**	54.9	(12.)	146	55.5	(12.)	157	0.704	-0.381	54.7	12.9	100	52.7	(10.8)	110	0.232	1.198	-0.2	(9.7)	91	-2.7	9.2	95	0.071	1.814
**Global Index Total**	57.6	(12.6)	144	57.2	(12.5)	158	0.76	0.306	56.0	11.7	100	54.5	(10.5)	110	0.334	0.969	-1.7	(8.4)	91	-3.2	7.9	96	0.22	1.231
**DSM-IV Inattention**	57.5	(11.9)	148	57.6	(11.8)	157	0.926	-0.093	57.3	11.2	100	55.1	(12.)	111	0.189	1.318	-1.7	(8.)	91	-2.9	10.5	96	0.368	0.902
**DSM-IV Hyperactive-Impulsive**	58.7	(12.8)	145	57.6	(13.4)	158	0.468	0.726	56.5	11.6	101	55.6	(11.6)	110	0.604	0.519	-2.8	(8.1)	92	-2.2	8.1	96	0.615	-0.504
**DSM-IV Total ADHD**	58.5	(12.6)	148	57.8	(12.6)	158	0.588	0.543	57.0	11.5	100	55.7	(10.9)	111	0.382	0.877	-2.6	(8.)	91	-2.6	8.5	97	0.99	0.013

*T-scores have a mean of 50 (sd = 10).

^†^Behaviour measures are derived from the Conners’ Parent Rating Scale (CPRS)

**Table 12:** Post-intervention mean differences (95% CI) for standardized* behavior measures—Parent rated^†^ (ITT).

	All randomized (n = 376)			Reading ≤ 10th Centile (n = 213):		
	Mean Difference	95%	CI	Mean Difference	95%	CI
**Oppositional**	-0.249	-2.024	1.525	-1.639	-4.068	0.79
**Cognitive Problems**	-1.615	-3.292	0.061	-3.222	-5.523	-0.92
**Hyperactivity**	-1.21	-3.043	0.623	-2.826	-5.315	-0.338
**Anxiety**	-0.491	-2.012	1.03	-0.887	-3.077	1.303
**Perfectionism**	-0.224	-1.748	1.301	-0.84	-2.963	1.282
**Social Problems**	0.433	-1.414	2.279	-1.016	-3.578	1.546
**Psychosomatic**	0.097	-2.011	2.204	-0.94	-3.603	1.724
**ADHD Index**	-0.987	-2.642	0.669	-3.293	-5.6	-0.985
**Global Restless-Impulsive**	-1.27	-2.978	0.439	-3.178	-5.604	-0.753
**Global Emotional Lability**	-1.433	-3.237	0.371	-3.082	-5.676	-0.487
**Global Index Total**	-1.144	-2.845	0.558	-3.266	-5.682	-0.85
**DSM-IV Inattention**	-1.949	-3.727	-0.171	-3.991	-6.527	-1.454
**DSM-IV Hyperactive-Impulsive**	-0.797	-2.571	0.977	-2.559	-4.919	-0.199
**DSM-IV Total ADHD**	-0.636	-2.354	1.081	-2.553	-4.923	-0.182

*T-scores have a mean of 50 (sd = 10).

^†^Behaviour measures are derived from the Conners’ Parent Rating Scale (CPRS).

1) Parent-ratings:

The ITT analyses showed a significant difference *in favor of the Placebo* group change scores for the Anxiety sub-scale (Active mean(sd) = -1.0(7.9), Placebo mean(sd) = -3.8(9.6), p(t)<0.01(3.123)) and for the Global change scores for Emotional Lability (Active mean(sd) = 0.9(9.6), Placebo mean(sd) = -2.8(9.8), p(t)<0.05(2.357) and DSM IV Inattention (Active mean(sd) = -1.0(8.9), Placebo mean(sd) = -3.2(10.2), p(t)<0.02(2.417).

In the per-protocol analyses (n = 187–8), no group differences were significant with the exception of a trend *in favor of the Placebo group* on the Anxiety sub-scale (Active mean(sd) = -0.8(7.3), Placebo mean(sd) = -4.1(9.3), p(t)<0.007(2.717)).

2) Teacher-ratings:

The ITT analyses (Table 13) showed that behavioral improvements (that is lower scores) as rated by teachers were greater for the Placebo group over Active treatment on the Anxiety sub-scale (Active mean = -0.5(10.8), Placebo mean(sd) = -3.7(11.0); p(t)<0.01(2.847)).

**Table 13:** Standardized* behavior measures—Teacher rated^†^ (ITT), means (sd).

	Baseline						Post-intervention						Change scores					
	Active (n = 187)	sd	Placebo (n = 189	sd	P	t	Active (n = 187)	sd	Placebo (n = 189	sd	P	t	Active (n = 187)	sd	Placebo (n = 189	sd	P	t
**Oppositional**	53.4	(11.5)	52.2	(10.)	0.291	1.057	54.2	(10.9)	53.8	(10.1)	0.694	0.393	0.8	(10.3)	1.5	(9.7)	0.467	-0.729
**Cognitive Problems**	66.6	(6.9)	66.8	(7.2)	0.795	-0.26	63.3	(7.7)	64.9	(7.9)	0.063	-1.868	-3.3	(7.3)	-2.0	(7.9)	0.093	-1.685
**Hyperactivity**	55.3	(9.5)	53.3	(9.6)	0.036	2.104	54.1	(8.5)	52.8	(8.6)	0.131	1.514	-1.2	(8.1)	-0.5	(8.4)	0.387	-0.865
**Anxiety**	58.7	(11.)	59.0	(11.)	0.799	-0.254	58.3	(10.5)	55.3	(10.)	0.006	2.752	-0.5	(10.8)	-3.7	(11.)	0.005	2.847
**Perfectionism**	46.9	(7.3)	46.4	(5.6)	0.477	0.713	48.2	(7.7)	46.7	(5.9)	0.038	2.079	1.3	(6.9)	0.3	(4.9)	0.109	1.605
**Social Problems**	53.4	(10.)	53.7	(10.8)	0.794	-0.262	53.8	(10.4)	52.8	(10.)	0.374	0.889	0.3	(9.5)	-0.9	(9.4)	0.211	1.252
**ADHD Index**	59.5	(8.9)	58.6	(9.4)	0.364	0.91	57.9	(8.8)	56.6	(9.4)	0.166	1.388	-1.6	(8.6)	-2.0	(9.1)	0.623	0.492
**Global Restless-Impulsive**	59.3	(8.9)	57.1	(9.7)	0.019	2.362	57.8	(8.8)	56.4	(9.2)	0.124	1.544	-1.5	(8.5)	-0.6	(9.1)	0.357	-0.922
**Global Emotional Lability**	54.0	(11.4)	52.5	(9.9)	0.186	1.326	54.2	(11.2)	51.8	(8.8)	0.021	2.325	0.2	(10.7)	-0.8	(8.6)	0.342	0.951
**Global Index Total**	58.8	(9.5)	56.7	(9.9)	0.036	2.11	57.8	(9.2)	55.5	(9.3)	0.014	2.481	-1.0	(8.7)	-1.2	(8.8)	0.773	0.288
**DSM-IV Inattention**	62.2	(8.2)	62.3	(8.4)	0.889	-0.14	59.6	(8.4)	59.4	(8.3)	0.772	0.289	-2.6	(7.9)	-3.0	(8.4)	0.66	0.441
**DSM-IV Hyperactive-Impulsive**	53.6	(9.7)	52.1	(9.8)	0.126	1.534	53.1	(8.9)	51.1	(9.2)	0.029	2.189	-0.5	(8.9)	-1.0	(8.7)	0.584	0.548
**DSM-IV Total ADHD**	59.2	(8.5)	58.6	(8.7)	0.469	0.724	57.9	(8.5)	57.0	(8.4)	0.312	1.013	-1.3	(8.1)	-1.5	(8.4)	0.779	0.281

*T-scores have a mean of 50 (sd = 10).

^**†**^Behaviour measures are derived from the Conners’ Teacher Rating Scale (CTRS).

However, these were not consistent across sub- and global scales and the per-protocol analyses (n = 196, Table 14), no significant effects of treatment were found. Table 15 further highlights this point due to the small group mean differences and corresponding 95% confidence intervals including zero.

**Table 14:** Standardized* behavior measures—Teacher rated^†^ (per-protocol), means (sd).

	Baseline								Post-intervention								Change scores							
	Active	sd	N	Placebo	sd	N	P	t	Active	sd	N	Placebo	sd	N	P	t	Active	sd	N (listwise)	Placebo	sd	N (listwise)	P	t
**Oppositional**	55.4	(12.9)	136	54.0	(11.5)	133	0.342	0.952	55.6	12.8	130	55.1	(12.1)	127	0.76	0.305	-0.3	(9.)	101	0.4	9.5	95	0.594	-0.534
**Cognitive Problems**	66.7	(8.1)	134	67.0	(8.6)	132	0.789	0.268	63.9	9.3	129	64.8	(9.7)	127	0.482	0.704	-3.1	(7.5)	101	-2.2	8.4	95	0.397	-0.849
**Hyperactivity**	55.8	(11.2)	135	54.6	(11.1)	133	0.374	0.890	54.8	10.1	130	53.6	(10.4)	127	0.359	0.919	-1.7	(9.)	101	-1.9	9.1	95	0.887	0.142
**Anxiety**	59.0	(12.9)	136	59.4	(13.1)	133	0.78	0.280	58.8	12.6	130	56.0	(12.2)	127	0.07	1.818	-1.4	(11.)	101	-2.7	11.2	95	0.405	0.834
**Perfectionism**	48.0	(8.4)	135	47.4	(6.5)	132	0.547	0.604	49.4	9.0	130	47.6	(7.)	127	0.074	1.792	0.5	(6.1)	101	0.1	5.2	95	0.64	0.469
**Social Problems**	54.8	(11.5)	135	55.3	(12.6)	133	0.723	0.355	55.4	12.1	130	54.2	(11.9)	127	0.423	0.802	-0.1	(8.9)	101	-0.8	9.7	95	0.619	0.497
**ADHD Index**	59.7	(10.5)	135	58.9	(11.2)	132	0.561	0.583	58.3	10.6	129	57.4	(11.4)	127	0.499	0.677	-2.0	(8.6)	101	-2.6	9.3	95	0.644	0.462
**Global Restless-Impulsive**	59.4	(10.5)	136	57.9	(11.4)	133	0.255	1.140	58.2	10.5	130	57.1	(11.2)	127	0.412	0.822	-1.9	(8.6)	101	-1.5	9.9	95	0.765	-0.299
**Global Emotional Lability**	55.5	(13.1)	135	53.6	(11.7)	132	0.212	1.252	55.6	13.2	129	53.1	(10.5)	127	0.097	1.666	-1.6	(10.1)	101	-0.8	9.7	95	0.585	-0.547
**Global Index Total**	59.1	(11.2)	135	57.4	(11.8)	133	0.223	1.222	58.2	11.1	129	56.7	(11.2)	127	0.268	1.111	-1.9	(8.9)	101	-1.4	9.5	95	0.703	-0.382
**DSM-IV Inattention**	62.3	(9.6)	135	62.5	(10.)	132	0.88	0.151	59.9	10.1	130	60.0	(10.1)	127	0.907	0.116	-2.8	(8.4)	101	-2.9	8.7	95	0.9	0.126
**DSM-IV Hyperactive-Impulsive**	54.6	(11.3)	135	53.4	(11.5)	132	0.379	0.880	54.1	10.6	129	52.6	(10.9)	127	0.267	1.112	-1.4	(9.5)	101	-1.8	9.0	95	0.756	0.311
**DSM-IV Total ADHD**	59.7	(10.)	135	59.2	(10.4)	132	0.729	0.347	58.3	10.2	129	57.5	(10.2)	127	0.537	0.619	-1.9	(8.6)	101	-2.5	9.1	95	0.68	0.414

*T-scores have a mean of 50 (sd = 10).

^†^Behaviour measures are derived from the Conners’ Teacher Rating Scale (CTRS).

**Table 15:** Post-intervention mean differences (95% CI) for standardized* behavior measures—Teacher rated^†^ (ITT).

	All randomized (n = 376)			Reading ≤ 10th Centile (n = 213):		
	Mean Difference	95%	CI	Mean Difference	95%	CI
**Oppositional**	-0.425	-2.551	1.701	-0.704	-3.744	2.336
**Cognitive Problems**	1.51	-0.079	3.099	0.695	-1.442	2.832
**Hyperactivity**	-1.337	-3.073	0.399	-1.395	-4.042	1.252
**Anxiety**	-2.913	-4.994	-0.832	-3.834	-6.534	-1.133
**Perfectionism**	-1.47	-2.861	-0.08	-2.021	-4.024	-0.018
**Social Problems**	-0.934	-2.998	1.131	-1.898	-4.747	0.951
**Psychosomatic**	-1.306	-3.156	0.544	-1.476	-4.028	1.075
**ADHD Index**	-1.432	-3.257	0.392	-1.368	-3.956	1.221
**Global Restless-Impulsive**	-2.409	-4.447	-0.371	-3.021	-6.056	0.014
**Global Emotional Lability**	-2.374	-4.255	-0.493	-2.63	-5.395	0.136
**Global Index Total**	-0.25	-1.948	1.448	-0.719	-3.1	1.662
**DSM-IV Inattention**	-2.044	-3.88	-0.208	-2.121	-4.914	0.672
**DSM-IV Hyperactive-Impulsive**	-0.883	-2.595	0.83	-1.203	-3.693	1.286
**DSM-IV Total ADHD**	-0.425	-2.551	1.701	-0.704	-3.744	2.336

*T-scores have a mean of 50 (sd = 10).

^†^Behaviour measures are derived from the Conners’ Parent Rating Scale (CPRS).

One systematic finding was the consistent reduction in the teacher ratings across both treatment groups.

### Multivariate robustness checks

The above results were check for robustness given the statistically inefficient use of change-scores as well as for the influence of the minimization factors gender and school. Multivariate (OLS) regressions resulted in the same overall conclusions and are reported in Supporting Materials—**Multivariate Analyses S1 Table.**

### Other measures

#### Adverse events

The DHA supplement provided is generally regarded as safe (G.R.A.S.) [32] and so no stopping guidelines were put in place except in the case of severe adverse events. As expected, there were none in the course of this trial. The parents of one child in each group reported episodes of diarrhoea and one child in the placebo group was diagnosed with Asperger’s and prescribed Ritalin during the course of the intervention. In addition, one school reported a negative behavior change in 9 children (4 in the Active and 5 in the Placebo group) and another school reported the onset of severe nose bleeds in a child in the Active group.

#### Health information and attendance

No group differences were found post-intervention either on child’s health status reported in the health questionnaire. No differences were found in school-reported “half-day absences for illness” between groups at post-intervention assessment. Those in the active group (n = 169) reported 4.9 (sd = 5.3) half day’s absence as compared to those in the placebo group (n = 170) who had 5.4 (sd = 6.2) half day’s absence, p = 0.63 (Wilcoxon-z = -0.31).

#### Reported side effects

No group differences were found for potential side effects assessed by the Barkley scale (Table 16 and Table 17).

**Table 16:** Scores for Barkley’s side effects questionnaire I (for all returned).

		Counts:										Test for association	
	N	Absent: 0	1	2	3	4	5	6	7	8	Serious: 9	Z(Wilcoxon)	Pvalue
Insomnia: Placebo	97	59	8	7	5	3	3	1	5	1	5	0.252	0.401
Active	106	63	14	7	9	3	2	2	3	1	2	.	.
Nightmares: Placebo	98	75	5	6	3	4	2	1	1		1	0.170	0.432
Active	107	81	16	2	1	2	3	0	1		1	.	.
Day Dreams: Placebo	98	51	6	15	9	3	4	1	3	2	4	0.801	0.212
Active	106	58	15	12	5	2	3	2	3	3	3	.	.
Talks Less: Placebo	98	82	5	2	2	1	2	2	1		1	-0.193	0.577
Active	106	87	7	4	1	4	2	1	0		0	.	.
Uninterested: Placebo	98	79	5	5	1	1	2	2	2	0	1	-0.511	0.695
Active	107	82	11	4	3	3	2	0	1	1	0	.	.
Decreased Appetite: Placebo	97	78	3	6	4	2	2		1	1		0.115	0.454
Active	106	86	3	7	2	2	5		1	0		.	.
Irritability: Placebo	98	39	11	12	6	10	10	4	2		4	0.876	0.190
Active	106	46	11	13	13	10	5	5	1		2	.	.
Stomach: Placebo	98	66	11	7	2	3	5		2	2		-0.412	0.660
Active	107	69	11	10	5	3	7		0	2			.
Headache: Placebo	98	70	10	6	2	3	4	2	1		0	-0.118	0.547
Active	107	76	10	4	6	6	1	1	2		1	.	.

**Table 17:** Scores for barkley’s side effects questionnaire II (for all returned).

		Counts:										Test for association	
	N	Absent: 0	1	2	3	4	5	6	7	8	Serious: 9	Z(Wilcoxon)	Pvalue
Drowsiness: Placebo	98	83	4	3	2	3		2	1			0.780	0.218
Active	107	94	6	3	2	2		0	0			.	.
Sad: Placebo	98	57	6	9	8	4	5	2	5		2	-0.067	0.527
Active	107	54	20	13	8	4	4	1	3		0	.	.
Crying: Placebo	98	55	12	8	7	1	5	1	3	3	3	0.193	0.423
Active	107	59	13	15	7	3	4	3	1	0	2	.	.
Anxious: Placebo	99	57	9	9	5	4	3	4	3	2	3	0.864	0.194
Active	109	66	13	11	4	6	3	2	4	0	0	.	.
Bites Nails: Placebo	97	57	8	3	4	4	3	1	5	1	11	1.383	0.083
Active	107	73	4	3	3	7	4	2	3	2	6	.	.
Euphoric: Placebo	98	70	5	6	4	6	4	1	1		1	0.633	0.263
Active	107	80	8	4	5	3	4	0	2		1	.	.
Dizziness: Placebo	98	92	2	1	1	1	1	0				0.162	0.436
Active	107	101	3	1	0	0	1	1				.	.
Tics: Placebo	98	85	1	3	1	3	1	2	0	1	1	-0.247	0.598
Active	107	91	5	3	2	1	1	1	1	1	1	.	.

#### Compliance

Counts of capsules returned by schools indicated mean compliance of approximately 75% and this did not significantly differ between Active (capsules were returned from n = 108 participants) and Placebo groups (capsules were returned from n = 104 participants). From 200 capsules allocated to schools for each child, quantities returned were: Active mean(sd) = 42.5(43.8) and Placebo mean(sd) = 48.9(48.8) (p(t)<0.317(-1.1)). Of the 142 capsules allocated to parents for non-school days, more than 50% of data were missing and so these are not reported.

**Fig 3:**
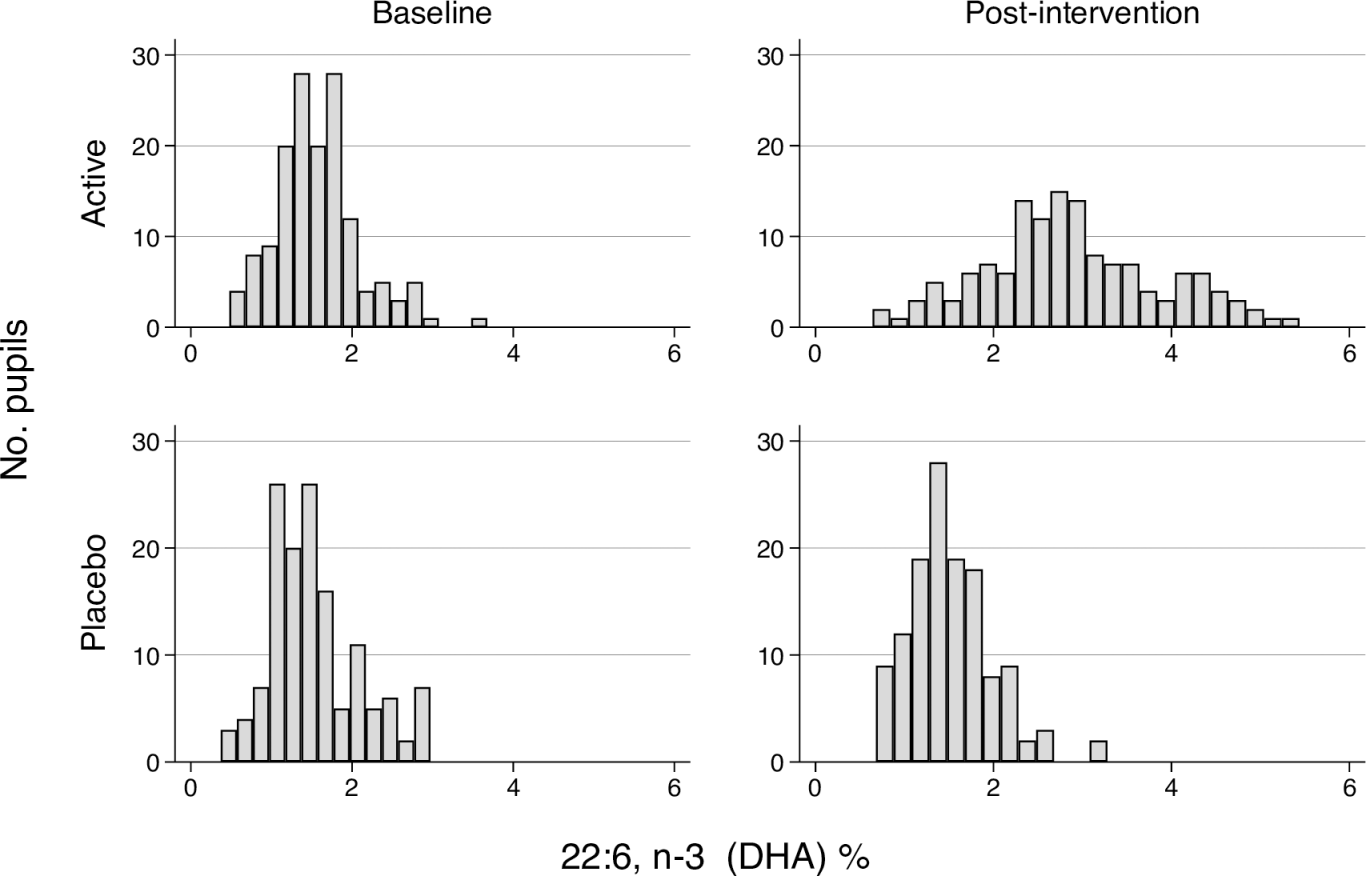
Blood DHA omega-3 (22:6) distributions by treatment group before and after intervention.

Objective data from fingerstick tests show that children in the active group had DHA levels of 2.9% (n = 140) compared to 1.5% in the placebo group (n = 129) (p(z)<0.001(11.3)) at post-intervention. Change scores indicate the active group increase their blood DHA from 1.6% to 2.9%, while the placebo group showed no such changes (p<0.001(10.54)). The baseline and post-intervention distribution of blood DHA levels by treatment group are illustrated in Fig 3. below.

## Discussion

With this randomized, control trial, we made every attempt to rigorously replicate our previous findings of an improvement in reading and behavior following a dietary supplementation with the omega-3 fatty acid DHA amongst school children aged 7–9 whose reading was initially below the 20^th^-centile of pupils. In line with the original DOLAB I study, our primary outcomes were changes in reading, working memory and behavior (ADHD-type symptoms, parent-rated). In summary, this study did not replicate the original findings of significant, positive effects of omega-3 DHA on either learning or behavior. No systematic adverse effects from the supplementation were observed. As such the study does not provide supporting evidence for the benefits of this safe nutritional intervention.

### Why did the DOLAB studies not replicate?

The results of the DOLAB II replication RCT and DOLAB I are clearly at odds. It is not entirely surprising that this study did not replicate the earlier one as has been found in many trials recently [33,34]. A number of substantive and necessary differences between the initial and the replication study might have contributed to these findings, despite the similar design of the two studies a combination of recruitment, measurement and uptake differences will have introduced considerable between-study heterogeneity.

First, the UK national curriculum relating to reading was changed in 2011 with a re-introduction of the phonic teaching approach. To address this change, a recalibrated version of the BAS II reading measure was used, which may, perhaps, have been less sensitive to detecting reading changes than its uncalibrated version.

Second, whilst the trial design of the DOLAB II replication RCT was identical to the initial study, we focused from the onset on the poorest reader amongst the pupils. Arguably this should have provided a higher power for detecting statistically significant intervention effects. However, the more restrictive inclusion criteria made recruitment more difficult. Compared to DOLAB I, pupils were recruited from five counties rather than one and the recruitment period was extended to 29 instead of 23 months. The larger recruitment area prevented the research team from repeated follow-up data collection visits, and consequently was identified as one source of the substantive missing teacher- and parent- self-report data.

Third, an additional recruitment challenge arose from the change of local authority run primary schools to self-governing academies, which had to be individually approached to gain school consent.

Fourthly, the recruitment issues further meant that a well-powered sample size of n = 400 was not quite fully achieved, and thus anticipated power gains by focusing on the subgroup of the 20^th^-centile readers were not fully realized. For illustrative purposes only, had we taken the observed effect size (d = 0.05) on the primary outcome–reading–the achieved power (α = 0.05) of this study would be 0.08 (8%), correspondingly to achieve 80% power given this effect size a sample of more than 11500 participants would have been necessary.

Finally, there appears to have been a lower omega 3 DHA uptake than in the previous trial, with DHA levels post-intervention being 2.9% as opposed to 3.8% in DOLAB I. However, changes in blood DHA levels bear no clear relationship in changes with primary outcomes when considering those with higher increases in DHA levels compared with those with lower increases or no changes (see **Supporting Information S8 File**).

### Contrasting with common challenges to replication

This study is a good example of the replication problems outlined in the literature [33], we will discuss key issues following from John Ioannidis seminal paper. Protocol *power* calculations indicated a sample size of n = 400 would be required and in the event n = 376 participants were recruited. Our achieved power calculations underscoring this point even further. Several potential sources of *bias* may have affected the results, however our preregistered protocol (**Protocol S1 File**) and CONSORT-compliant (**Checklist S2 File)** reporting attends to most of these and provides transparency through the study. For example, clear hypotheses and a *preselected* (and reported) outcomes are provided therein. Both implementers and assessors were blinded to treatment group. Further, data and analysis syntax (Stata dofile) are available without restriction through the Open Science Framework: https://osf.io/9ynjf.

For additional analyses. Systematic reviews and *other studies of this question* provide inconsistent results, as they include heterogeneous groups of participants, interventions, comparators and outcomes [10,11,16–20]. Furthermore, there are implementation differences in dose, delivery, uptake and context both generally [35], specifically to this field [36], and with regard to this trial as discussed (see above). Consequently, the *ratio of true to no relationships* in the area of fatty-acid supplementation is problematic, and this is partly due to the large number of small studies finding small effects which are known to provide a poor basis for replication. This is arguably a complex intervention to evaluate [37], with multiple modes of delivery and outcome (child, parent, school), long causal pathway (bio-psycho-social mechanism for a behavioral change), where proximal (16-week) outcomes may not indicate distal change. This study was conducted without direct influence of its funder by way of a robust contract, there may remain researcher biases (self-serving, consistency and allegiance [38]) but again, transparent reporting guidelines aim to address these matters.

Finally, the reporting of these null-results illustrates our commitment to avoid publication biases, and our conviction that these add to the knowledge base on nutritional interventions. At a minimum, these studies contribute to the increased power of systematic reviews and meta-analyses.

### Implications for research and practice

This study serves as an example for the need for robust, comparable trials for replication. Standardization of populations, interventions in terms of dose, composition and delivery would help evaluate the evidence base for this safe intervention. Currently trials use a range of placebos making comparisons difficult and result in mixed and vague outcomes. This poses a particular challenge to systematic reviews and meta-analysis trying to establish the best available evidence. The development of a core outcome set for similar trials on nutrition, learning, and behavior would be helpful [39]. Secular changes, such as reading curricula updates, may make replication challenging. And thus, even if the design and setting of studies are comparable non-replication will occur as this study demonstrated.

## Supporting information

S1 File Protocol. (DOCX)

S2 File CONSORT checklist. (DOCX)

S3 File Further details on recruitment. (DOCX)

S4 File Capsule content. (DOCX)

S5 File Protocol amendment change of capsule shell. (DOCX)

S6 File Blood fatty acid data- methods. (DOCX)

S7 File Randomization technical details. (DOCX)

S8 File Prediction of learning and behaviour from DHA change. (DOCX)

S1 Table Multivariate analyses. (DOCX)
