# Supplementary material for: Docosahexaenoic acid for reading, working memory and behavior in UK children aged 7-9: A randomized controlled trial for replication (the DOLAB II study)
Source: PLoS One. 2018 Feb 20;13(2):e0192909. doi: 10.1371/journal.pone.0192909 (PMC5819802; doi:10.1371/journal.pone.0192909)
Supplement: S1 File — (DOCX) [file pone.0192909.s001.docx]

**The DHA (Docosahexaenoic acid) Oxford Learning And Behaviour (DOLAB) II Study**

**PROTOCOL**

Funder: DSM Nutritional Products

Principal Investigators: Professor Paul Montgomery* & Dr Alex Richardson

(*Acting as Chief Investigator)

Centre for Evidence Based Intervention

University of Oxford

Sponsor: University of Oxford

Version 9: 16^th^ May 2014

# TABLE OF CONTENTS

[TABLE OF CONTENTS i](#_Toc378856099)

[PROTOCOL SUMMARY 3](#_Toc378856100)

[Research Questions 3](#_Toc378856101)

[BACKGROUND AND RATIONALE 4](#_Toc378856102)

[Omega-3 for child behaviour and learning 4](#_Toc378856103)

[Study Population 5](#_Toc378856104)

[Age and Sex 5](#_Toc378856105)

[Outcome Measures 6](#_Toc378856106)

[Objectives 6](#_Toc378856107)

[STUDY DESIGN 7](#_Toc378856108)

[Design 7](#_Toc378856109)

[Primary Endpoint 7](#_Toc378856110)

[Secondary Endpoints 7](#_Toc378856111)

[STUDY POPULATION 7](#_Toc378856112)

[Selection of participants 7](#_Toc378856113)

[Inclusion criteria 7](#_Toc378856114)

[Exclusion criteria 8](#_Toc378856115)

[Selection of schools 8](#_Toc378856116)

[School Inclusion criteria 8](#_Toc378856117)

[School Exclusion criteria 8](#_Toc378856118)

[ENROLMENT 8](#_Toc378856119)

[Screening 9](#_Toc378856120)

[Enrolment/ Baseline Assessments 9](#_Toc378856121)

[Randomisation Process 9](#_Toc378856122)

[STUDY TREATMENTS 9](#_Toc378856123)

[Active supplementation 10](#_Toc378856124)

[Placebo 10](#_Toc378856125)

[Supply and Labeling 10](#_Toc378856126)

[Unblinding of Individual Participants During The Study 10](#_Toc378856127)

[Assessment of Compliance with Study Treatment/s 11](#_Toc378856128)

[Early withdrawal 11](#_Toc378856129)

[Participant Transfer and Withdrawal 11](#_Toc378856130)

[Participant Transfers 11](#_Toc378856131)

[Withdrawal from Study Intervention 11](#_Toc378856132)

[Withdrawal from Study Completely 12](#_Toc378856133)

[Loss to Follow-up 12](#_Toc378856134)

[ASSESSMENTS AND PROCEDURES (1) - Overview 12](#_Toc378856135)

[Study Schedule 13](#_Toc378856136)

[Screening 13](#_Toc378856137)

[Baseline 14](#_Toc378856138)

[Bi-weekly 14](#_Toc378856139)

[Post-intervention 14](#_Toc378856140)

[ASSESSMENTS AND PROCEDURES (2) – Details of measures 15](#_Toc378856141)

[Screening 15](#_Toc378856142)

[Key Stage 1 Reading score 15](#_Toc378856143)

[Baseline and post-intervention 15](#_Toc378856144)

[Conners Teacher and Parent Ratings (CTRS-L and CPRS-L) 15](#_Toc378856145)

[British Ability Scales Word reading 15](#_Toc378856146)

[British Ability Scale (BAS II) Recall of Digits 16](#_Toc378856147)

[Fingerprick blood measures to assess fatty acid status and compliance 16](#_Toc378856148)

[Prediction of treatment group 16](#_Toc378856149)

[Side effects scale 16](#_Toc378856150)

[Attendance 17](#_Toc378856151)

[STATISTICAL CONSIDERATIONS 17](#_Toc378856152)

[Method of Randomisation 17](#_Toc378856153)

[Outcome Measures 17](#_Toc378856154)

[Primary outcome 17](#_Toc378856155)

[Secondary outcomes 17](#_Toc378856156)

[Sample Size 18](#_Toc378856157)

[Analysis Plan 18](#_Toc378856158)

[ADVERSE EVENT COLLECTION 18](#_Toc378856159)

[Reporting Procedures for Serious Adverse Events 19](#_Toc378856160)

[Potential Risks and Benefits of Omega 3s 19](#_Toc378856161)

[Potential Risks 19](#_Toc378856162)

[Potential Benefits 19](#_Toc378856163)

[ETHICAL CONSIDERATIONS 20](#_Toc378856164)

[Ethical considerations 20](#_Toc378856165)

[Ethical Approval 20](#_Toc378856166)

[Informed Consent Process 20](#_Toc378856167)

[STUDY MONITORING 21](#_Toc378856168)

[Site Monitoring 21](#_Toc378856169)

[Direct Access to Data 21](#_Toc378856170)

[Confidentiality 21](#_Toc378856171)

[Quality Assurance and Quality Control of Data 21](#_Toc378856172)

[Records Retention 22](#_Toc378856173)

[INDEMNITY 22](#_Toc378856174)

[FINANCIAL ARRANGEMENTS 22](#_Toc378856175)

[Payroll Staff - Roles and Responsibilities 22](#_Toc378856176)

[STUDY REGISTRATION AND STEERING COMMITTEE 23](#_Toc378856177)

[PUBLICATIONS 23](#_Toc378856178)

[PROTOCOL AMENDMENTS 25](#_Toc378856179)

[REFERENCES 26](#_Toc378856180)

# PROTOCOL SUMMARY

**Title:** The DHA(docosahexaenoic acid) Oxford Learning and Behaviour (DOLAB) II Study.

**Type of Study:** The study is a randomized, placebo controlled trial (RCT), double blind, fixed dose, parallel groups.

**Population:** Four hundred children from academic year groups 3, 4 and 5 (who are generally aged 7 to 9 years) attending mainstream schools who are currently underperforming in reading (lowest quintile on nationally standardized achievement tests).

**Number of Sites:** Up to two hundred schools from up to eight local authorities as required.

**Intervention:** The active compound (DHA Omega-3), 600mg per day) and corn/soy placebo (matching in package, taste and appearance) will be administered in an oral supplement.

**Study Duration and process:**

The parents/guardians of children who are underperforming in reading (according to national tests or school records) will be invited to consent to their child participating in a short school-based session, involving brief assessments of reading and working memory, an optional pinprick blood sample, and behaviour ratings from their teachers and parents. Our previous study (DOLAB) indicated that we should anticipate that 1300 children will need to be screened at the Local Authority stage. Of those approximately half are likely to consent to participate in these brief assessments at school. Those children whose parents consent, and whose reading assessment scores place them in the lowest quintile (on a standardized reading test), will be invited to join the study provided they meet the other inclusion/exclusion criteria (n = 400). Baseline measures will be collected after which children will be randomly allocated to DHA Omega-3 or placebo conditions and followed up after 16 weeks. Recruitment will be carried out over two years.

## Research Questions

**Primary: Can supplementation with DHA Omega-3 improve child behaviour and learning in underperforming children in year groups 3, 4 and 5 (who are generally aged 7-9 years)?** Outcomes assessed will include reading performance, working memory and ADHD-type symptoms (inattention, hyperactivity, impulsivity) as rated by parents.

**Secondary: Can supplementation with DHA Omega-3 improve child ADHD-type symptoms (inattention, hyperactivity, impulsivity) as rated by teachers?**

**Do children’s levels of DHA Omega-3 or related fatty acids (**assessed objectively via a pinprick blood sample**) predict their learning or behaviour?**

**Are any observed changes in behaviour or cognitive performance associated with changes in blood fatty acid status?**

#

# BACKGROUND AND RATIONALE

## Omega-3 for child behaviour and learning

Evidence from human clinical trials has shown that an increased dietary intake of the highly unsaturated Omega 3 fatty acids found in seafood (principally EPA and DHA) may have significant benefits for various aspects of mood, behaviour and learning (Freeman *et al.,* 2006). Indeed, accumulating evidence from epidemiological, biochemical and intervention studies suggests that low dietary intakes of these Omega-3 may have a detrimental effect on children’s behaviour and cognitive development (Schuchardt *et al.,* 2010, Ryan *et al*., 2010).

Until recently 4 previous controlled treatment trials in children had shown benefits for behaviour and/or learning from supplementation with these Omega 3 (Richardson and Puri 2002, Stevens *et al* 2003, Richardson and Montgomery 2005, Sinn & Bryan 2006), while two others had not (Voigt *et al*.,2001, Hirayama *et al.,* 2004).

Almost all such studies had involved populations with specific developmental conditions such as attention-deficit/hyperactivity disorder (ADHD), dyslexia or developmental coordination disorder (DCD). They were also small, with considerable differences between the populations studied, treatment formulations used, and outcomes assessed. Findings from these randomised controlled trials (RCTs) were therefore mixed, but the most consistently reported benefits in children of school age have included improvements in attention and concentration, and reductions in other ‘ADHD-type’ symptoms (particularly impulsive and oppositional behaviour, anxiety, and emotional lability) (Richardson, 2006, Bloch and Qawasmi, 2011). Highly significant improvements in both reading and spelling performance were also found in the one study that assessed these outcomes (Richardson and Montgomery, 2005).

These findings raised the important question of whether any such results might have broader applicability, so the DOLAB study (a double blind, placebo controlled parallel group RCT) was designed to explore whether healthy children (in year groups 3, 4 and 5) from a larger general school population might also benefit from Omega 3 supplementation. Rather than target the entire population (most of who had little or no room to improve on the outcomes of interest) the study focused on those children whose current reading age ability placed them within the bottom 33% of the school children.

Analyses of baseline data from this study showed that children with higher blood DHA Omega-3 status was associated both with a better level of reading and working memory but also better parent-rated behaviour and less teacher-rated anxiety. In addition the RCT showed promising results for those children in the active supplement group, particularly for the poorest readers (those in the bottom 20^th^ centile on a standardised reading assessment). Both reading ability and parent reported behaviour showed a significant improvement over the course of the intervention.

If these results can be confirmed in a larger population, the implications would be profound since there is an urgent need for safe, effective interventions for child learning and behaviour problems, which create substantial costs for society and affected individuals.

.

## Study Population

As noted above, the first DOLAB trial was designed to explore whether previously reported benefits for child behaviour and learning from DHA Omega-3 supplementation may extend to the general school population.

Findings from this study showed a significant improvement in reading ability for those children whose reading fell into the bottom 20% on a standardised reading assessment at baseline. Indeed, results appear to show that the more severe reading impairment at baseline, the greater the positive impact of the intervention.

- **This study is therefore designed to replicate and strengthen the findings of the first DOLAB trial. The population will be larger than the previous trial and will involve up to 200 schools. It will once again include children attending mainstream primary schools whose reading ability at baseline falls into the *bottom 20%* but who are otherwise healthy and who have no other significant learning difficulty according to their teachers.**

## **Age and Sex**

Behaviour and learning difficulties usually compound with age, so early intervention is always preferable, but our clinical and research experience indicates that 7-9 year olds are the age group most likely to show significant improvements in literacy skills following DHA Omega-3 supplementation (o*r other inter*ventions). Standardised national assessments of performance in a range of key areas (including reading) are carried out at ages 7, 11 and 14 on all UK children attending state schools,^[[1]](#footnote-1)^ and results from these can be used to select children who are underperforming for their age.

Findings from the DOLAB study show that those children who benefit most from the intervention do not differ significantly by age. For these reasons, this study will once again involve children from year groups 3, 4 and 5. Previous trials in this area of clinical groups have included mainly boys, who are more likely than girls to show overt behavioural and/or learning difficulties.

The DOLAB study involved equal numbers of boys and girls, and showed that those children who benefitted most from the intervention did not differ significantly by sex. This study will therefore focus simply on children who are underperforming for their age, although possible sex differences will be explored in the analyses.

## Outcome Measures

Since the aim in this new study is to replicate and strengthen the findings of the first DOLAB trial, the outcome measures chosen will be identical. Behaviour will again be assessed by both parents and teachers, using the well-validated Conners’ Scales (Conners 1997). These scales were originally developed to assess pharmacological treatments for child behaviour problems, and have shown benefits in four of the successful RCTs of Omega-3 for child behaviour and learning (Richardson & Puri, 2002; Richardson & Montgomery, 2005; Sinn & Bryan 2006, Richardson et al, 2012)

With respect to cognitive function, simple, transparent measures are again likely to be the most useful. The Oxford-Durham study showed clear benefits from fatty acid treatment for literacy skills (reading and spelling), and also on a simple, widely used test of working memory. More recently the DOLAB study has again shown promising gains in age-standardised tests of reading performance and working memory and these measures will therefore be used once again as primary outcomes in this study. Further, attendance at school will be captured by way of school records and parent report to assess the possible impact of the intervention on this important outcome.

## Objectives

Primary objective

As reported above, results from the DOLAB study have shown an improvement in both reading ability and parent reported behaviour in healthy children from year groups 3, 4 and 5 (who are generally aged 7-9 years) from mainstream state schools who are underperforming in reading but who have no other significant learning difficulties. The primary purpose of this trial is therefore to replicate the finding that DHA Omega-3 (in a daily dose of 600 mg) does indeed benefit this population of poorer readers and to establish further knowledge on the size of this effect in a larger sample of children.

Secondary objectives:

Analyses from the DOLAB study have shown that levels of DHA Omega-3 in this population of children as a whole appear to be remarkably low, under 2% compared with concentrations of at least 3-4% DHA Omega-3 generally considered to be the minimum needed to maintain general and cardiovascular health in adults. In addition findings from the DOLAB study have shown an association between DHA Omega-3 concentration and both cognitive function and behaviour (Montgomery et al, in preparation).

Secondary objectives for this new study will be to gain further knowledge on Omega-3 status in a larger population of children but also evaluate sickness and missing school as well as explore further the association between blood biochemical measures of fatty acid status and behavioural, cognitive and health outcomes at baseline and in terms of changes following treatment.

# STUDY DESIGN

## Design

This is a randomized controlled trial of DHA Omega-3 versus placebo for 16 weeks (fixed dose, parallel group double blind study).

## Primary Endpoint

1. To confirm or refute that supplementation with DHA Omega-3, (600mg/day) for 16 weeks improves learning and parent-reported behaviour in children from year groups 3, 4 and 5 (who are generally aged 7-9 years) from mainstream schools who are underperforming in reading.

## Secondary Endpoints

1. To assess whether supplementation with DHA Omega-3 (600 mg/day) for 16 weeks improves teacher reported behaviour.

2. To assess whether levels of DHA Omega-3 or related fatty acids (assessed objectively via a pinprick blood sample) predict their learning or behaviour.

3. To assess whether any changes in behaviour or cognitive performance are associated with changes in objective measures of blood fatty acid status.

4. To assess whether DHA Omega-3 influences health and/or school attendance

# STUDY POPULATION

## Selection of participants

### Inclusion criteria

1. Children from year groups 3, 4 and 5 from mainstream state schools who are underperforming in literacy skills according to nationally standardized assessments of scholastic achievement at age 7 years (Key Stage 1^[[2]](#footnote-2)^). To be eligible, children must score ≤ 20th centile for reading, but are not judged by their teachers to have any other significant learning difficulties.
2. English as a first language.

### Exclusion criteria

1. Major learning disabilities or medical disorders
2. Taking medications expected to affect behaviour and learning
3. Taking omega 3 supplements already, or eating fish > 2 x week

## Selection of schools

Outline permission for the study has already been given by Senior Executives at Oxfordshire, Milton Keynes, Wiltshire and Northamptonshire Local Authorities along with Swindon Unitary Authority. Further Local Authorities will be recruited should these be necessary.

### School Inclusion criteria

- Mainstream local authority primary schools in Oxfordshire and other local authorities as required.
- Educational authority approval
- Agreement of head and participating teachers

### School Exclusion criteria

- Not meeting the inclusion criteria listed above

Subject to the above, each participating school will be carefully identified on the basis of:

- Having at least one teacher with a specific interest in, and responsibility for, supervising and managing suitable children.
- Showing enthusiasm to participate in the study.
- Ensuring that sufficient time, staff and adequate facilities are available for the trial.
- Providing information to all supporting staff members, parents and children involved with the study.
- Acknowledging and agreeing to conform to the administrative and ethical requirements and responsibilities of the study.

# ENROLMENT

## Screening

Initial screening to assess a child’s possible eligibility for the study is based on the results of age 7+ Key Stage I assessments. Since for some children this assessment will have taken place one or two years previously, teachers in individual schools will asked to confirm whether the children screened this way are still eligible for the study and will be excluded at this stage if not. In the same way, teachers will also be asked to add the names of any children whose reading has fallen behind since their Key Stage 1 assessment.

Parents/carers of these eligible children will be provided with information on the study and invited to consent to their child’s participation. Fully informed written proxy consent (and assent) to participate in the study will be required before any baseline assessments are carried out.

Children whose parents consent to their taking part in the study will be assessed in schools and only those whose reading falls into the bottom 20^th^ centile on the BAS word reading measure will be invited to take part in the RCT.

## Enrolment/ Baseline Assessments

The following baseline measures will be obtained on participating children:

1. Teacher: Conners Teacher Ratings (CTRS-L) (Conners 1997) Attendance at school over previous term
2. Parent: Conners Parent Ratings (CPRS-L) (Conners 1997) Demographics Health questionnaire
3. Child: Word reading and Recall of digits from the British Ability Scales – BAS 3 (Elliot, 2011) and BAS II (Elliott, 1997) Fingerprick blood measures to assess fatty acid status and compliance

## Randomisation Process

1. Fully informed written proxy consent
2. Confirmation of eligibility, randomisation and issue of allocated treatment by way of a computer generated sequence which will ensure blinding of the study.

# STUDY TREATMENTS

The study is a simple parallel group randomised double-blind controlled trial comparing the effects of active supplementation with DHA Omega-3 at a fixed dose (600mg DHA Omega-3 per day) versus taste and colour-matched corn/soy placebo. DHA Omega-3 or placebo supplements will be supplied in numbered bottles to be delivered once daily to participating children over 16 weeks by teachers (Monday – Friday) and by parents/caregivers at weekends and during school holidays.

## Active supplementation

The active intervention will consist of 3 x 500 mg capsules per day, each capsule providing 200 mg of DHA Omega-3 as a triglyceride. The liquid fill contains DHA-S oil derived from the microalgae, Schizochytrium sp., high-oleic sunflower oil, natural mixed tocopherols, ascorbyl palmitate, rosemary extract, natural orange flavouring and natural masker. The vegetarian gelatine shell contains carrageenan, non-GMO modified cornstarch, glycerine, sorbitol, water, betacarotene and caramel powder.

## Placebo

The placebo will consist of 3 x 500 mg capsules per day containing corn/soy oil. The dimensions, taste, appearance and colour will be identical to those of the DHA Omega-3 capsules. The shell of the capsule will be the same as the DHA Omega-3 capsule. The liquid fill contains corn/soy oil, natural orange flavouring, natural masker, tocoblend L70 IP, rosemary oil and ascorbyl palmitate.

## Supply and Labeling

The active supplement and placebo will be supplied in coded bottles for dispensing by schools and parents. Each participant will be required to consume 3 capsules daily (600 mg of DHA Omega-3). Each bottle will be labeled with a 2-part label. The inner portion contains the protocol number, the participant random allocation number, a place to record the participant’s initials, storage instructions, sponsor and PI’s name and address and an Investigational Product Statement. The outer tear-off portion of the bottle label contains the protocol number, the participant random allocation number and a place to record the participant’s initials.

## Unblinding of Individual Participants During The Study

Unblinding will be considered only when knowledge of the treatment assignment is deemed essential for the child’s care by their physician. In general, unblinding of participants during the conduct of the study is not allowed unless there are compelling medical or safety reasons to do so. The decision to unblind a single case will only be made when knowledge of an individual’s allocated treatment is essential to:

- Enable treatment of severe adverse event/s, or
- Enable administration of another therapy that is contraindicated by the study treatment.

Where possible, the agreement of the Chief Investigator or designee will be sought before requests for individual unblinding are made. Unblinding will be performed by the independent randomizing service Sealed Enveloped Ltd.

## Assessment of Compliance with Study Treatment/s

At the end of each child’s participation in the study, a member of the research staff will collect all unused supplements allocated to that child, and a pill count will be conducted and recorded to determine the doses administered. All unused capsules collected will be returned to the research centre where they will be retained for the duration of the study; at the end of the study they will be collected for destruction.

Blood fatty acid measures will provide an additional objective measure of compliance.

## Early withdrawal

If a participant wishes to withdraw from the study, all unused supplements will be collected and a pill count conducted and recorded. The unused supplements will be returned and retained for the duration of the study; at the end of the study they will be collected for destruction.

## Participant Transfer and Withdrawal

In consenting to the study, participants are consenting to study treatment, follow-up and data collection. If voluntary withdrawal occurs, the parent/caregiver will be asked to allow continuation of scheduled evaluations and to complete an end-of-study evaluation. Follow-up of these participants will be continued through the study.

### Participant Transfers

Where possible we will aim to screen for these in advance and exclude if the child is known to be moving. If any child does transfer we will attempt to follow him/her up at the new school.

### Withdrawal from Study Intervention

Participants may be withdrawn from the intervention for any of the following reasons:

1. Parent/carer of the child withdraws consent for treatment.
2. Unacceptable adverse effects.
3. Intercurrent illness preventing further participation.
4. Development of serious disease preventing further participation.
5. Any change in the participants’ condition that justifies the discontinuation of participation in the opinion of parent, teacher or clinician.

If a parent/carer wishes to withdraw a child from the intervention, schools will inform the project administrator who should document the reason. The project coordinator will attempt to contact the parent/carer, explain the importance of remaining in the study and being followed-up, and request that data be collected as per the study schedule. Generally, follow-up will continue unless the parent/carer explicitly also withdraws consent for follow-up (see following section).

### Withdrawal from Study Completely

Parents/caregivers are free to withdraw consent at any time without providing a reason. Those who do this will have anonymised data collected up to the point of that withdrawal of consent included in the analyses. The child will no longer receive a daily dose of either active treatment or placebo. Data up to the time of withdrawal will be included in the analyses unless the parent/carer explicitly states that this is not their wish.

## Loss to Follow-up

If any of the study participants are lost to follow up, contact will initially be attempted through the school and parent address.

#

# ASSESSMENTS AND PROCEDURES (1) - Overview

The following assessments and procedures schedule will be will be followed during the course of the study.

Assessment and procedures Schedule – DOLAB II

| Procedure | Screening | Baseline | Bi-Weekly Contact | Post-  intervention |
| --- | --- | --- | --- | --- |
|  |  | Day 7 +/- 2 d |  | Day 112 +/- 2d |
| Informed Consent | X |  |  |  |
| Prior & Concomitant Medications | X | X |  | X |
| Inclusion/Exclusion | X |  |  |  |
| Finger Prick (optional) |  | X |  | X |
| BAS 3 Reading Test (C) |  | X |  | X |
| BAS II Reading Test (C) |  | X |  | X |
| BAS II Working Memory Digit Span (C) |  | X |  | X |
| Conners Teacher Ratings (T) |  | X |  | X |
| Connors Parent Ratings (P) |  | X |  | X |
| Demographics (P) |  | X |  |  |
| Randomisation |  | X |  |  |
| Prediction Test Group (T, C, P) |  |  |  | X |
| Barkley Side Effects Rating Scale |  |  |  | X |
| Adverse Events |  |  | X | X |
| Capsule Compliance |  |  | X | X |
| Illness Tracking Questions |  |  | X | X |

Recruitment will be carried out over two years. There will be two assessment points for children: i) screening/baseline, and ii) post-intervention for children who are randomized to treatment.

This section will outline the overall study schedule, the measures that will be administered at each of the assessment points (and by whom), how they will be performed and what will be done with these data.

The following section will provide further details of each of the assessments used.

## Study Schedule

The study will run for 36 months: September 2012 to August 2015. It is anticipated that recruitment will be carried out between September 2012 and January 2015.

NB: The exact study schedule will be planned in detail with participating schools according to term length and any school activities which could interfere with testing sessions (such as examination, sporting or other activities).

Data analysis and write up will be conducted between April 2015 and August 2015.

## Screening

Children from year groups 3, 4 and 5 whose Key Stage 1 scores at age 7 years indicate they are within the lowest quintile for literacy, but whose teachers do not judge them to have any other significant learning difficulties.

Research staff will check with the school whether the child meets other eligibility criteria, i.e. has no major learning disabilities or medical disorders.

If the child passes this initial school screening stage, teachers will be asked to confirm whether their reading is still a cause for concern. If it is not they will no longer be considered for inclusion in the study. Teachers will be asked to add the names of any additional children whose reading shows room for improvement. These children will be assessed to ensure they meet inclusion/exclusion criteria.

Parents of potential participants will be contacted via a letter of invitation from their child’s school briefly describing the study. More detailed participant information will also be mailed with this letter along with a simple version for children health and demographic questionnaires, the consent form and an envelope addressed to their child’s school.

The invitation letter will inform parents that schools will be phoning them to request permission to pass on their contact details to the research team, in line with Data Protection policy. Unless parents express a wish not to be contacted, researchers will then telephone them so that they are given the opportunity to ask questions and discuss the study. If necessary, arrangements will be made to either visit the parents at home or meet them at school.

Parents of children randomized into the study will be asked to provide assessments at baseline and again at post-intervention. It will be made clear to parents that if their child is randomised to take part in the intervention they will be asked to give their child supplements each day during weekends and school holidays.

## Baseline

Confirmation of parent’s written consent and child verbal assent is required before any baseline assessment can be carried out in schools. The following measures will be administered in the school by the research staff:

Teacher: Conners Teacher Ratings

Attendance over past term (days missed due to illness)

Child: Standardised Reading test

Working memory digit span test

Fingerprick blood measures to assess compliance

At this point assessment measures will be scored by researchers. Children who meet inclusion criteria will be identified and randomization will be performed by telephone.

The following measures will be collected from parents using Freepost mail services and chased up with telephone calls or visits as required.

Parent: Conners Parent Ratings

Health questionnaire

Demographics

## Bi-weekly

Parents will be telephoned to check on any absences their child may have had from school, due to illness, once a fortnight. Details will be logged about any such illnesses, for example upper respiratory illness (nose and throat), lower respiratory illness (lungs, chest), ear infection, vomiting, diarrhea, rash or ectopy, food allergy, asthma, fever, lower GI (gastritis).

## Post-intervention

Teacher: Conners Teacher Ratings

Attendance over past term (days missed due to illness)

Prediction of treatment group

Child: Reading test age

Working memory digit span test

Fingerprick blood measures to assess compliance

Prediction of treatment group

The following measures will be collected from parents using Freepost mail services and chased up with telephone calls or visits as required.

Parent: Conners Parent Ratings

Prediction of treatment group

Health questionnaire

Barkley Side Effects Rating Scale (SERS) (Barkley, 1990)

A final telephone call will be made to parents to check on any absences their child may have had from school, due to illness. Details will be logged about any such illnesses

Participants in both stages of the study will be given a 3 month supply of DHA Omega-3 (Life’s DHA, provided by DSM Nutritional Products) as a token of thanks for their participation in the study.

In due course, the parents of all participating children will also receive details of the overall study results, as will all participating schools.

# ASSESSMENTS AND PROCEDURES (2) – Details of measures

## Screening

### Key Stage 1 Reading score

Key Stage tests are nationally standardized assessments taken by all UK children at ages 7, 11 and 14. They encompass five areas: speaking and listening, reading, writing, science and mathematics.

Reading scores from Key Stage 1 tests will be used to screen for children performing in the lowest quintile for their age against national norms at age 7. To be eligible for the study, children must not be judged by teachers to have any other significant learning difficulties.

## Baseline and post-intervention

### Conners Teacher and Parent Ratings (CTRS-L and CPRS-L)

These are standardized highly valid and reliable scales which measure child behaviour over several domains, yielding age-standardised scores for 7 different sub-scales (6 for teacher ratings) and 7 global scales.

These scales have shown significant improvements following fatty acid supplementation in several previous trials, and have been routinely used in medication trials for children with behaviour problems such as ADHD.

### British Ability Scales Word reading

This is an age standardized single word reading test from the British Ability Scales, normed on UK children It is administered to each child individually, taking only a few minutes to complete.

The BAS II version of this measure was used successfully in the Oxford-Durham trial and more recently in the first DOLAB study. This reading assessment is sensitive enough to show significant change over three to four months and will be used for replication purposes.

However, following recent policy changes an alternate-form scale whose content reflects changes in national curricula over the past 15 years has been developed (Elliot, 2011). In light of this, the BAS3 will be used for screening purposes and assessment.

### British Ability Scale (BAS II) Recall of Digits

This is a widely accepted measure of ‘working memory’ from the British Ability Scales. Again it is age standardized and administered at the individual level.

Working memory problems are common to a wide range of different conditions in which Omega-3 supplementation has been found beneficial including dyslexia, dyspraxia, ADHD, depression and age-related cognitive decline. Improvements in working memory are also likely to benefit many different aspects of cognitive performance in the general population.

### Fingerprick blood measures to assess fatty acid status and compliance

A fingerprick blood test will be performed at baseline and at the end of the study for randomized children in order to assess levels of fatty acids including docosahexaenoic acid (DHA Omega-3), arachidonic acid (AA), and eicosapentaenoic acid (EPA Omega-3). These blood samples (labeled by ID No. only) will be collected by way of absorbent paper sealed in a tube and stored in a specific refridgerator before being sent by courier to DSM Nutritional Products in the United States. Both DSM and the investigators will remain blinded to the allocation group until treatment codes are unlocked.

The fatty acid data will be used to verify compliance and to determine the relationship between levels of fatty acids in the blood and specific endpoints.

This blood test will be optional and will appear as a separate item on the consent form, but every effort will be made to maximize participation.

### Prediction of treatment group

The value of this measure is that it tests that the participants have remained blind to their group allocation.

### Side effects scale

Side effects will be measured post-intervention in all randomised children using the Barkley Side Effects Rating Scale (SERS) (Barkley 1990). This scale was designed for use in studies of stimulant medication for ADHD-type symptoms. Based on our own and others’ previous studies, negative side effects are not expected from supplementation with DHA Omega-3, but we are conscious that few studies specifically ask about these effects, so the use of this scale will redress that omission.

### Attendance

Attendance will be measured by way of school records pre and post-intervention. Parents will also be contacted fortnightly in order to establish any absences and the nature of any illnesses during the course of trial. We will make a distinction between absences due to illness and those due to, for example, holidays during term-time.

# STATISTICAL CONSIDERATIONS

A separate and full statistical analysis plan will be developed prior to the analysis of the study. The protocol will be discussed and agreed by the Trial Steering Committee (see below).

##

## Method of Randomisation

Randomisation of children will be conducted using dedicated computer software. The randomisation programme will include a minimisation algorithm to ensure balanced allocation of participants across the treatment groups by sex and school (to allow for any sociodemographic/school differences).

## Outcome Measures

### Primary outcome

- Can supplementation with DHA Omega-3 improve child behaviour and learning in underachieving children in year groups 3, 4 and 5 (who are generally aged 7-9 years)? Outcomes assessed will include reading performance, working memory and ADHD-type symptoms (inattention, hyperactivity, impulsivity) as rated by parents.

### Secondary outcomes

- Can supplementation with DHA Omega-3 improve child ADHD-type symptoms (inattention, hyperactivity, impulsivity) as rated by teachers?
- Do children’s levels of DHA Omega-3 or related fatty acids (assess objectively via a pinprick blood sample) predict their learning or behaviour?
- Are any changes in behaviour or cognitive performance associated with changes in blood fatty acid status?
- Sickness measures, school days missed.

## Sample Size

Power calculations (using post-intervention standardised reading change scores on the bottom 20^th^ centile readers from the DOLAB study) indicates that an n of 400 (199 per treatment arm) is an appropriate target when desired power is set at .05 alpha level.

## Analysis Plan

The study will be analysed and reported following the ‘CONSORT’ guidelines (Consolidated Standard of Reporting Trials) (Moher, 2001).

On completion of the study, data from all randomised children will be analysed on an intention to treat basis.

All analyses will be pre-specified in detail in the statistical analysis plan to be drawn up and agreed by the Trial Steering Committee, but will include subgroup analyses for the bottom 10% readers in line with exploratory findings (Richardson et al 2012). Missing data will be handled using various imputation methods. The robustness of the complete case analysis will be assessed (with a worst and best case) sensitivity analysis, however these will be informed by data collected on the reasons for any missing data.

# ADVERSE EVENT COLLECTION

Parents will be telephoned to check on any absences their child may have had from school, due to illness, once a fortnight. Details will be logged about any such illnesses, for example upper respiratory illness (nose and throat), lower respiratory illness (lungs, chest), ear infection, vomiting, diarrhoea, rash or ectopy, food allergy, asthma, fever, lower GI (gastritis). During this telephone call parents will be asked about any adverse events which may have occurred.

Definition of Serious Adverse Events

A serious adverse event is any untoward medical occurrence that:

- Results in death,
- Is life-threatening,

NOTE: The term "life-threatening" in the definition of "serious" refers to an event in which the participant was at risk of death at the time of the event; it does not refer to an event which hypothetically might have caused death if it were more severe.

- Requires inpatient hospitalisation or prolongation of existing hospitalisation,
- Results in persistent or significant disability/incapacity, or
- Is a congenital anomaly/birth defect.
- Other important medical events.*

*Other events that may not result in death, are not life threatening, or do not require hospitalisation, may be considered a serious adverse event when, based upon appropriate medical judgement, the event may jeopardise the participant and may require medical or surgical intervention to prevent one of the outcomes listed above.

## Reporting Procedures for Serious Adverse Events

A serious adverse event (SAE) occurring to participant should be reported to the REC that gave a favourable opinion of the study where in the opinion of the Chief Investigator the event was: ‘related’ – that is, it resulted from administration of any of the research procedures; and ‘unexpected’ – that is, the type of event is not listed in the protocol as an expected occurrence. Reports of related and unexpected SAEs should be submitted within 15 days of the Chief Investigator becoming aware of the event, using the NRES [report of serious adverse event](http://www.nres.npsa.nhs.uk/docs/forms/Safety_Report_Form_(non-CTIMPs).doc) form (see IRAS/NRES website).

The Chief Investigator shall inform DSM of any SAEs in relation to the use of the Study Product within a maximum of 24 hours after the University is made aware of the SAE. The SAE reports will not contain any information which could identify any study participant. On completion of the study, the Chief Investigator shall provide DSM will a list of SAE’s occurring throughout the study period.

## Potential Risks and Benefits of Omega 3s

### Potential Risks

None are known. According to the US FDA, 3g per day of EPA + DHA is generally regarded as safe (GRAS). This study will use only 600mg.

Clinical studies of Omega-3 supplementation in humans (adult and child volunteers and patients of both sexes and all ages) have not shown any consistent or serious short or long-term adverse side-effects.

DSM Nutritional Products’ DHA-S is already widely used in human nutrition as it is used in adult, maternal and child supplements as well as a variety of food and beverage products. The FDA considers both DHA-S and corn/soy oil to be “highly refined oils” that are not associated with allergic reactions.^[[3]](#footnote-3)^

### Potential Benefits

Clearly, benefits can never be guaranteed for individual participants in any research trial, but average intakes of DHA in the UK and other developed countries are widely acknowledged to be suboptimal for general health (Hibbeln *et al* 2006). Adequate supplies of DHA are essential for optimal development and functioning of the brain and nervous system (particularly for vision), and for cardiovascular and immune health.

As noted above, preliminary RCT evidence from studies of healthy children has already shown improvements in attention, concentration and behaviour, and benefits for literacy skills. The aim of this study is to confirm and extend these findings.

# ETHICAL CONSIDERATIONS

## Ethical considerations

The study will abide by the principles of the Declaration of Helsinki and subsequent revisions. We consider the specific ethical issues relating to participation in this study to be:

- Taking a placebo (dummy) treatment or the active (DHA Omega-3) treatment.

There is no single, 'gold standard' treatment that is used to combat scholastic underachievement and/or behaviour problems in children. Those drugs that are sometimes prescribed for behaviour problems associated with ADHD have obvious and unpleasant side-effects that would make blinding difficult in any case, but more fundamentally, they are not an appropriate intervention for normal children who are simply underperforming at school. Placebo is therefore an appropriate comparator because (a) there is no standard treatment and (b) double-blinding enables the true treatment effect of DHA Omega-3 to be investigated.

- Case Report Forms and Assessments in English only

To provide appropriate translations of all study documentation and assessments would require checks and validations of language, as well as a comprehensive review of cultural and linguistic influences on the study measures. The latter include age-standardised tests of literacy skills, which are likely to be affected by the primary language spoken. We unfortunately do not have the resources to fulfil these standards adequately and therefore must limit study entry to children with English as a first language.

## Ethical Approval

No study procedures will take place until full approval has been given by an NHS ethics committee.

## Informed Consent Process

Informed consent is a process initiated prior to an individual agreeing to participate in a study and continues throughout the individual’s participation. Informed consent is required for all individuals participating in coordinated studies. In obtaining and documenting informed consent, the investigators will comply with applicable regulatory requirements and adhere to the ethical principles that have their origin in the Declaration of Helsinki.

# STUDY MONITORING

## Site Monitoring

### Direct Access to Data

Site monitoring may be deemed to be necessary as a result of central data checks. In order to perform their role effectively, the trial coordinator and others involved in Quality Assurance and Inspection may need direct access to primary data, eg. school records, relevant medical reports etc. Since this affects the participant’s confidentiality, this fact is included on the Participant Information Sheet and Informed Consent Form.

### Confidentiality

Individual participant medical information obtained as a result of this study is considered confidential, and disclosure to third parties is prohibited with the exceptions noted below.

Electronic and paper record forms will be labeled with participant’s unique trial registration and/or randomisation number. Verification of appropriate informed consent will be enabled by the provision of copies of participants’ signed informed consent/assent forms being supplied to the schools.

The Centre for Evidence Based Intervention (CEBI) will preserve the confidentiality of participants taking part in the study and The University of Oxford is registered under the Data Protection Act. Access to anonymised data may be given to authorized individuals from both the University of Oxford and DSM Nutritional Products for the purposes of monitoring and/or audit.

### Quality Assurance and Quality Control of Data

This study will undergo a risk assessment, the outcome of which is likely to indicate that it is a low risk study. As such, site visits will be conducted and source data reviewed if indicated to be required as a result of central monitoring processes. To this end:

- The Investigators, the Trial Coordinator and other staff will attend the launch meetings for key staff from participating schools, which will incorporate elements of study-specific training necessary to fulfill protocol requirements.
- The Trial Coordinator will verify that appropriate approvals are in place prior to initiation of a school, and to check that the relevant personnel have attended study-specific training.
- The Trial Coordinator will check safety and compliance reporting rates between centres.
- The Trial Coordinator will monitor screening, recruitment and drop-out rates between schools.
- The Trial Coordinator will conduct data entry consistency checks and follow-up data queries.
- Independent oversight of the study will be provided by the Trial Steering Committee.

##

## Records Retention

The CEBI undertake to store originally completed record forms and separate copies of the above documents for up to a maximum of 3 years or until the University informs the investigators that the documents are no longer to be retained.

Arrangements will be made to ensure the continued storage of the documents, even if the investigators, for example, leave the University or retire before the end of required storage period. Delegation will be documented in writing, and the investigators will archive all relevant source documents so that the study data can be compared against source data after completion of the study.

# INDEMNITY

Indemnity for this study will be provided by the University of Oxford.

# FINANCIAL ARRANGEMENTS

These have been negotiated between the Research Services Office at Oxford University and DSM Nutritional Products

### Payroll Staff - Roles and Responsibilities

***The Chief Investigator*** will take overall responsibility for the research, including the trial design; directing, managing and supervising the implementation of the study; data analyses; and reporting and disseminating of the findings. He will ensure that all phases of research adhere to the highest scientific, ethical and other professional standards.

***The Study Coordinator*** will liaise with schools and Local Authority staff to set up efficient systems for screening, recruitment, administration of treatments and data collection. The Coordinator will be responsible for the day to day running of the study and data management, including supervising the data collection assistants throughout the course of the study. The Coordinator will also work closely with Professor Montgomery and Dr. Richardson, assisting with the preparation of the final study protocol and research ethics applications, and also with the writing up of research publications.

***The Research Assistant*** will assist the Study Coordinator with all of the duties listed above. In addition they will be responsible for the helping with both data entry and cleaning. The RA will also be responsible for, along with the Study Coordinator, overseeing the all routine administration, including distribution and collection of information and consent forms, and questionnaire measures; and ensuring that supplies of intervention and placebo are delivered to and collected from schools according to protocol.

***Data Collection Assistants*** (part-time, session-based) will visit schools under the Study Coordinator’s direction and supervision, as well as carrying out some home visits if required (to collect data needed from parents’ questionnaires. Their role will centre on administration of questionnaire and other measures at screening, baseline and post-intervention, but will also involve ‘troubleshooting’, i.e. chasing up of participants when necessary (e.g. if scheduled school appointments cannot be met)

# STUDY REGISTRATION AND STEERING COMMITTEE

This trial will be registered in advance with the Current Controlled Trials (<http://www.controlled-trials.com/>).

The Trial Steering Committee will consist of:

- Professor Chris Bonell, Professor of Sociology and Social Intervention, University of Oxford
- Professor Frances Gardner, Professor of Child and Family Psychology, University of Oxford
- Dr Thees Spreckelsen, University of Oxford (Empirical Social Scientist)
- Dr Alexandra Richardson, University of Oxford
- Professor Paul Montgomery, University of Oxford

The Trial Steering Committee will be consulted regularly and its role will be to provide guidance and advice on the following topics:

- Recruitment
- Randomisation
- Trial progress
- Protocol amendments
- Ethics
- Budget
- Adverse events and serious adverse events

# PUBLICATIONS

It is anticipated that at least two strong academic papers will be forthcoming from this study: one on the primary outcomes (to address whether DHA Omega-3 improves child behaviour and learning) and a second on the objective blood biochemical measures and their correlations with the outcome measures. The data generated should also provide material for further academic publications in addition to these major ones.

All academic papers will be produced independently and submitted to journals such as PLoS, Pediatrics, Lipids, the British Medical Journal and Archives of Disease in Childhood where the authors have published in the past.

Professor Paul Montgomery

Dr Alexandra Richardson

Principal Investigators

# PROTOCOL AMENDMENTS

Version 8: 30^th^ January, 2014

This version has been amended to reflect additional information received on the capsule make up since the start of the study (see page 10). Previously this section read as follows:

Active supplementation

The active intervention will consist of 3 x 500 mg capsules per day, each capsule providing 200 mg of DHA Omega-3 as a triglyceride. The liquid fill contains DHA-S oil, derived from the microalgae, Schizochytrium sp., high-oleic sunflower oil, natural mixed tocopherols, ascorbyl palmitate, and rosemary extract, and orange extract (flavouring). The gelatin shell contains glycerin, water, and colouring (caramel, carmine, turmeric).

Version 9: 16^th^ May, 2014

This version has been amended to reflect a new formulation for the capsules to be used in the study from September 2014 onwards (page 10). This change in ingredients arises from concerns raised in the above Protocol Amendment (Version 8, January 2014). Previously this section read as follows:

Active supplementation

The active intervention will consist of 3 x 500 mg capsules per day, each capsule providing 200 mg of DHA Omega-3 as a triglyceride. The liquid fill contains DHA-S oil, derived from the microalgae, Schizochytrium sp., high-oleic sunflower oil, natural mixed tocopherols, ascorbyl palmitate, and rosemary extract, and orange extract (flavouring). The animal gelatin shell contains glycerin, water, and colouring (Sunset yellow (E110)) [*Sentence amended on 10^th^ January 2014*].^[[4]](#footnote-4)^

# REFERENCES

Barkley RA (1990). Attention-Deficit Hyperactivity Disorder: A Handbook for Diagnosis and Treatment. New York, NY: Guilford Press

Bloch MH and Qawasmi, A (2011). Omega 3 fatty acid supplementation for the treatment of children with attention-deficit hyperactivity disorder symptomatology:systematic review and meta-analysis. Journal of the American Academy of Child and Adolescent Psychiatry, 50, (10), 991-1000.

Burd L, Klug MG, Coumbe MJ. & Kerbeshian J (2003). Children and adolescents with attention deficit-hyperactivity disorder: 1. Prevalence and cost of care. Journal of Child Neurology, 18, 555-561.

Connors CK (1997). Conners’ Parenting Rating Scales – Revised. Technical manual. New York: Multi-Health Systems Inc.

Elliot, C., Smith, P. & McCulloch, K. (1996) British Ability Scales: Second Edition (BAS II). Windsor, UK: NFER-Nelson.

Elliot, C with Smith, P. (2011) British Ability Scales: Third Edition (BAS 3). GL Assessment Ltd. London.

FDA (2005). The Threshold Working Group of the FDA. Approaches to Establish Thresholds for Major Food Allergens and for Gluten in Food. U.S. Food and Drug Administration, June 2005. Available at <http://www.cfsan.fda.gov/~dms/alrgn.html>

Freeman MP, Hibbeln JR, Wisner KL, Davis JM, Mischoulon D, Peet M, Keck PE Jr, Marangell LB, Richardson AJ, Lake J, Stoll AL (2006). Omega-3 fatty acids: evidence basis for treatment and future research in psychiatry. Journal of Clinical Psychiatry, 67(12): 1954-67.

Hirayama, S., Hamazaki, T. & Terasawa, K. (2004). Effect of docosahexaenoic acid-containing food administration on symptoms of attention-deficit/hyperactivity disorder - a placebo-controlled double-blind study. European Journal of Clinical Nutrition, 58, 467-473.

Hibbeln JR, Nieminen LRG, Blasbalg TL, Riggs JA, Lands WEM (2006). Healthy intakes of n-3 and n-6 fatty acids: estimations considering worldwide diversity. American Journal of Clinical Nutrition, **83**(suppl) 1483S-93S

Moher D, Schulz KF & Altman D (2001). The CONSORT statement: revised recommendations for improving the quality of reports of parallel-group randomised trials. Lancet, 357, 1191-1194.

Montgomery P, Stores G & Wiggs L (2004). The relative efficacy of two brief treatments for sleep problems in young learning disabled (mentally retarded) children: a randomised controlled trial. Archives of Disease in Childhood, 89 (2) 125-130.

NIH (1998). NIH Consensus Statement: Diagnosis and Treatment of Attention Deficit Hyperactivity Disorder (ADHD). Bethesda: National Institutes of Health.

Owens, J. A., Spirito, A., & McGuinn, M. (2000). The Children’s Sleep Habits Questionnaire. (CSHQ): Psychometric properties of a survey instrument for school-aged children. *Sleep, 23*(8), 1-9.

Richman N & Graham PJ (1971). A behaviour screening questionnaire for use with three year old children. Journal of Child Psychology and Psychiatry, 12, 5.33.

Richardson AJ, Montgomery P (2005). The Oxford-Durham study: a randomized controlled trial of dietary supplementation with fatty acids in children with developmental coordination disorder. Pediatrics 115 (5) 1360-1366.

Richardson AJ (2006). Omega-3 fatty acids in ADHD and related neurodevelopmental disorders. Int Rev Psychiatry, 18(2):155-72.

Richardson AJ, Puri BK (2002). A randomized double-blind, placebo-controlled study of the effects of supplementation with highly unsaturated fatty acids on ADHD-related symptoms in children with specific learning difficulties. Prog Neuropsychopharm Biol Psychiat, 26(2) 233-239.

Ryan AS, Astwood JD, Gautier S, Kuratko CN, Nelson EB, Salem N Jr. (2010) Effects of long-chain polyunsaturated fatty acid supplementation on neurodevelopment in childhood: a review of human studies. Prostaglandins Leukot Essent Fatty Acids. 2010 Apr-Jun;82(4-6):305-14. Epub Feb 25.

Schuchardt JP, Huss M, Stauss-Grabo M, et al. Significance of long-chain polyunsaturated fatty acids (PUFAs) for the development and behaviour of children. Eur J Pediatr 2010; 169:149–164.

Sinn N, Bryan J (2007). Effect of supplementation with polyunsaturated fatty acids and micronutrients on learning and behaviour problems associated with child ADHD Journal of Developmental & Behavioural Pediatrics 28 82-91

Stevens L, Zhang W, Peck L, Kuczek T, Grevstat N, Mahon A, Zentall SS, Arnold LE & Burgess JR (2003). EFA supplementation in children with inattention, hyperactivity and other disruptive behaviours. Lipids, 38, 1007-1021.

Voigt RG, Llorente AM, Jensen CL, Fraley JK, Berretta MC & Heird WC (2001). A randomized, double-blind, placebo-controlled trial of docosahexaenoic acid supplementation in children with attention-deficit/hyperactivity disorder. Journal of Pediatrics, 139, 189-196.

Wechsler D (1993). Wechsler Objective Reading Dimensions (WORD): London, United Kingdon. The Psychological Corporation.

Wechsler D. (1991). Wechsler Intelligence Scale for Children-111. San Antonio, TX: The Psychological Corporation

Yehuda S, Rabinovitz S, Mostofsky DI (1998) Essential fatty acids and sleep: mini-review and hypothesis. Med Hypotheses. 50(2):139-45

Yehuda S, Rabinovitz S, Carasso RL, Mostofsky DI. (2007) Pretreatment with a mixture of essential fatty acids protects rats from anxiogenic effects of REM deprivation. Nutr Neurosci, 10(5-6):269-71.

1. These are known as ‘Key Stage’ assessments, and cover five major domains: reading, writing, speaking & listening, maths and science [↑](#footnote-ref-1)
2. Key Stage 1 assessments are carried out for all children attending UK state schools at the end of the school year in which they reach 7 years of age. The tests are nationally consistent, and cover five main areas: reading, writing, speaking and listening, maths, and science. [↑](#footnote-ref-2)
3. In general, edible oils can be derived from major food allergens such as soybeans and peanuts, and may contain variable levels of protein. “The consumption of highly refined oils derived from major food allergens by individuals who are allergic to the source food does not appear to be associated with allergic reactions.” (The Threshold Working Group of the FDA, 2005). [↑](#footnote-ref-3)
4. The oil encapsulation is performed by a validated Good Manufacturing Processing facility. The gelatin used for encapsulation protects the oil from oxidation and results in a capsule shelf life of two years or more when stored at room temperature. [↑](#footnote-ref-4)
