# Supplementary material for: Docosahexaenoic acid for reading, working memory and behavior in UK children aged 7-9: A randomized controlled trial for replication (the DOLAB II study)
Source: PLoS One. 2018 Feb 20;13(2):e0192909. doi: 10.1371/journal.pone.0192909 (PMC5819802; doi:10.1371/journal.pone.0192909)
Supplement: S2 File — (DOCX) [file pone.0192909.s002.docx]

## S2 – CONSORT Checklist

As set out in the discussion, the DOLAB II study should be regard as a complex social intervention. Consequently, the authors opted to use the draft of the new extended CONSORT-SPI check list (Montgomery et al. 2013) to accommodate the differences to more traditional randomized controlled trials.

*Check list below.*

*Reference:*

*Montgomery, P., Grant, S., Hopewell, S., Macdonald, G., Moher, D., Michie, S., & Mayo-*Wilson, E. (2013). Protocol for CONSORT-SPI: An extension for social and psychological interventions. *Implementation Science, 8*: 99. DOI: [10.1186/1748-5908-8-99](http://dx.doi.org/10.1186/1748-5908-8-99).

**The CONSORT-SPI Checklist***

| **Section** | **Item #** | **CONSORT 2010** | **CONSORT-SPI** |
| --- | --- | --- | --- |
| **Title and abstract** |  |  |  |
|  | 1a | Identification as a randomised trial in the title**^§^** | **Done** |
|  | 1b | Structured summary of trial design, methods, results, and conclusions (for specific guidance see CONSORT for abstracts)**^§^** | **Done** |
| **Introduction** |  |  |  |
| Background and objectives | 2a | Scientific background and explanation of rationale**^§^** | Line 62-114 Page 3/5 |
|  | 2b | Specific objectives or hypotheses**^§^** | If pre-specified, how the intervention was hypothesised to work  Lines 115-119 Page 5 |
| **Methods** |  |  |  |
| Trial Design | 3a | Description of trial design (such as parallel, factorial) including allocation ratio**^§^** | If the unit of random assignment is not the individual, please refer to CONSORT for Cluster Randomised Trials  Lines 120-124 Page 5 |
|  | 3b | Important changes to methods after trial commencement (such as eligibility criteria), with reasons | Lines 178-179 Page 7 |
| Participants | 4a | Eligibility criteria for participants**^§^** | When applicable, eligibility criteria for settings and those delivering the interventions  Lines 125-155 Page 5/6 |
|  | 4b | Settings and locations where the data were collected | Lines 147-151 Page 6 |
| Interventions | 5 | The interventions for each group with sufficient details to allow replication, including how and when they were actually administered**^§^** | Extent to which interventions were delivered and taken up as planned, including what they actually involved Lines 163-179 Page 7 |
|  |  |  | Where other informational materials about delivering the intervention can be accessed |
|  |  |  | When applicable, how intervention providers were assigned to each group  Lines 249-256 Page 10 |
| **Section** | **Item #** | **CONSORT 2010** | **CONSORT-SPI** |
| Outcomes | 6a | Completely defined pre-specified primary and secondary outcome measures, including how and when they were assessed**^§^** | Lines 180-237 Pages 7-9 |
|  | 6b | Any changes to trial outcomes after the trial commenced, with reasons | Lines 129-138 Page 5-6 |
| Sample Size | 7a | How sample size was determined**^§^** | Lines 245-248 Page 10 |
|  | 7b | When applicable, explanation of any interim analyses and stopping guidelines | Lines 428-429 Page 31 |
| *Randomisation:* |  |  |  |
| Sequence generation | 8a | Method used to generate the random allocation sequence | Lines 249-256 and S7 Page 10 |
|  | 8b | Type of randomization; details of any restriction (such as blocking and block size)**^§^** | Lines 249-256 and S7 Page 10 |
| Allocation concealment Mechanism | 9 | Mechanism used to implement the random allocation sequence (such as sequentially numbered containers), describing any steps taken to conceal the sequence until interventions were assigned**^§^** | Lines 249-256 and S7 Page 10 |
| Implementation | 10 | Where applicable, who generated the random allocation sequence, who enrolled participants, and who assigned participants to interventions**^§^** | Lines 249-256 and S7 Page 10 |
| Awareness of Assignment | 11a | Who was aware of intervention assignment after allocation (for example, participants, providers, those assessing outcomes), and how any masking was done | Lines 257-261 Page 11 |
|  | 11b | If relevant, description of the similarity of interventions | Lines 163-168 and S4 Page 7 |
| Analytical methods | 12a | Statistical methods used to compare groups for primary and secondary outcomes**^§^** | How missing data were handled, with details of any imputation method  Lines 262-268 Page 11 |
|  | 12b | Methods for additional analyses, such as subgroup analyses and adjusted analyses | Lines 280-281 Page 11 |
| **Section** | **Item #** | **CONSORT 2010** | **CONSORT-SPI** |
| **Results** |  |  |  |
| Participant flow (a diagram is strongly recommended) | 13a | For each group, the numbers randomly assigned, receiving the intended intervention, and analysed for the primary outcome**^§^** | Where possible, the number approached, screened, and eligible prior to random assignment, with reasons for dropout  Lines 286-296 Page 12 and Fig 1 |
|  | 13b | For each group, losses and exclusions after randomization, together with reasons**^§^** | Lines 286-296 Page 12 and Fig |
| Recruitment | 14a | Dates defining the periods of recruitment and follow-up | Line 288-289 Page 12 |
|  | 14b | Why the trial ended or was stopped | Line 288 Page 12 |
| Baseline data | 15 | A table showing baseline characteristics for each group**^§^** | Include socioeconomic variables where applicable  Lines 297-324 Page 12-16 And Table 1 & 2 |
| Numbers analysed | 16 | For each group, number included in each analysis and whether the analysis was by original assigned groups**^§^** | Lines 332-337 Page 19 |
| Outcomes and estimation | 17a | For each primary and secondary outcome, results for each group, and the estimated effect size and its precision (such as 95% confidence interval)**^§^** | Indicate availability of trial data  Lines 338-425 Page 19-31, Data: Line 283 |
|  | 17b | For binary outcomes, presentation of both absolute and relative effect sizes is recommended | None |
| Ancillary analyses | 18 | Results of any other analyses performed, including subgroup analyses and adjusted analyses, distinguishing pre-specified from exploratory | Lines 338-455 Page 19-33 |
| Harms | 19 | All important harms or unintended effects in each group (for specific guidance see CONSORT for harms) | Line 427-442 Page 32 |
| **Discussion** |  |  |  |
| Limitations | 20 | Trial limitations, addressing sources of potential bias, imprecision, and, if relevant, multiplicity of analyses | Lines 470-526 Page 34-37 |
| Generalisability | 21 | Generalisability (external validity, applicability) of the trial findings**^§^** | Lines 527-546 Page 36-37 |
| **Section** | **Item #** | **CONSORT 2010** | **CONSORT-SPI** |
| Interpretation | 22 | Interpretation consistent with results, balancing benefits and harms, and considering other relevant evidence | Lines 527-546 Page 36-37 |
| **Important information** |  |  |  |
| Registration | 23 | Registration number and name of trial registry | www.controlled-trials.com (ISRCTN48803273) |
| Protocol | 24 | Where the full trial protocol can be accessed, if available | **S1** |
| Declaration of Interests | 25 | Sources of funding and other support, role of funders | Declaration of any other potential interests Lines 53-60 Page 2 |
| *Stakeholder involvement | New Item |  | Any involvement of the intervention developer in the design, conduct, analysis, and reporting of the trial  None |
|  |  |  | Other stakeholder involvement in trial design, conduct, and/or analyses  None |
|  |  |  | Incentives offered as part of the trial  Lines 243-244 Page 10 |

This table lists CONSORT 2010 checklist items with modifications or additions for social and psychological intervention RCTs; empty items in the CONSORT-SPI column indicate no modification of the CONSORT 2010 item.

*We strongly recommended that the CONSORT-SPI Explanation and Elaboration (E&E) document be reviewed when using the CONSORT-SPI checklist for important clarifications on each item (see below).

§Indicates that an extension item for cluster trials exists for this CONSORT 2010 item.
