# Supplementary material for: Docosahexaenoic acid for reading, working memory and behavior in UK children aged 7-9: A randomized controlled trial for replication (the DOLAB II study)
Source: PLoS One. 2018 Feb 20;13(2):e0192909. doi: 10.1371/journal.pone.0192909 (PMC5819802; doi:10.1371/journal.pone.0192909)
Supplement: S3 File — (DOCX) [file pone.0192909.s003.docx]

## S3 – Further Details on Recruitment

Using local authority and Key Stage 1 information, schools were identified and contacted if it appeared there was a sufficient cohort of children whose reading was considered significantly below average. This information was not available for self-governing primary academies, which were approached if principals had expressed an interest following emails and flyer drops.
