# Supplementary material for: Docosahexaenoic acid for reading, working memory and behavior in UK children aged 7-9: A randomized controlled trial for replication (the DOLAB II study)
Source: PLoS One. 2018 Feb 20;13(2):e0192909. doi: 10.1371/journal.pone.0192909 (PMC5819802; doi:10.1371/journal.pone.0192909)
Supplement: S4 File — (DOCX) [file pone.0192909.s004.docx]

## S4 – Capsule content

The capsule shells of the treatment where change half-way through the study, the protocol and trial registration was amended accordingly (see S1 – Trial Protocol, yellow highlights).

| **STUDY PRODUCT** | | | |  | |
| --- | --- | --- | --- | --- | --- |
|  |  | Capsules prior to amendment | | Capsules after the amendment | |
|  |  | Active | Placebo | Active | Placebo |
| **Saturated** |  |  |  |  |  |
| (14:0) |  | 4 | <0.1 | 4.5 | <0.1 |
| (16:0) |  | 12.9 | 10.5 | 13.8 | 10.9 |
| (18:0) |  | 1 | 3 | 0.9 | 3.1 |
| **Mono-unsaturated** | |  |  |  |  |
| Oelic (18:1 n-9) | | 16.3 | 24.1 | 15.1 | 25.3 |
| **Omega-3** |  |  |  |  |  |
| ALA (18:3 n-3) | | 0.1 | 3.8 | <0.1 | 3.5 |
| EPA (20:5 n-3) | | 1.3 | <0.1 | 1.4 | <0.1 |
| DHA (22:6 n-3)* | | 37.5 | <0.1 | 37.3 | <0.1 |
| **Omega-6** |  |  |  |  |  |
| LA (18:2 n-6) | | 1.4 | 52.7 | 1.2 | 51.6 |
| DPA (22:5 n-6) | | 15.7 | <0.1 | 15.7 | <0.1 |
| **CAPSULE SHELL** | |  |  |  |  |
|  | | Gelatin (European Procine bone) | | Carrageenan, |  |
|  | | Glycerol BP | | Glycerine BP |  |
|  | | White titanium paste | | Non-GMO cornstarch | |
| **Coloring:** | |  |  |  |  |
|  |  | Sunset yellow (E110) (0.13%) | | Betacarotene Caramel powder. | |
| **Shell Weight:** | | 194mg | 194mg | 194mg | 194mg |
| **Other** | |  |  |  |  |
| Placebo only | | Tocoblend L70 IP | | Tocoblend L70 IP | |
|  | | Rosemary Oil | | Rosemary Oil | |
|  | | Ascorbyl palmitate  Orange Flavouring  Bitterness Masker | | Ascorbyl palmitate  Orange Flavouring  Bitterness Masker | |
|  | | Refined corn oil | | Refined corn oil | |
| *Derived from Microalgae (Schizochytrium sp.) | | | |  | |
