# Supplementary material for: Docosahexaenoic acid for reading, working memory and behavior in UK children aged 7-9: A randomized controlled trial for replication (the DOLAB II study)
Source: PLoS One. 2018 Feb 20;13(2):e0192909. doi: 10.1371/journal.pone.0192909 (PMC5819802; doi:10.1371/journal.pone.0192909)
Supplement: S5 File — (DOCX) [file pone.0192909.s005.docx]

## S5 - Protocol amendment – Intervention -Change of capsule shell

In December 2014, it became apparent that the shell of the capsules contained animal gelatine (despite the oil itself being from a plant source and thus suitable for vegetarians) and a colourant (E110, “Sunset Yellow FCF”) that had been linked to behavior and attentional problems in children (McCann et al. (1)). Since the intervention was a) specifically targeted at behavior and was advertised as suitable for vegetarians the formulation of the capsule shell had to be changed during the trial.

This was reported to both the supplier and also the Bristol Research Ethics committee responsible for the approval of the study on 10^th^ January 2014. Two substantial amendments were submitted and approved by the appropriate Ethics Committee in order 1) to continue recruitment, and 2) to use the new capsules once these became available. These changes are documented through amendments to the trial registration page (<http://www.isrctn.com/ISRCTN48803273> ). Parents of children already recruited to the study were informed. No participants subsequently dropped out. As a result, capsules containing neither of these substances were manufactured and used in the remainder of the study.

Post-hoc analyses revealed no substantial or statistical significant differences in the primary study outcomes between those children receiving the initial capsules and the remanufactured capsules.

*Reference:*

(1) McCann, D. et al., 2007. Food additives and hyperactive behaviour in 3-year-old and 8/9-year-old children in the community: a randomised, double-blinded, placebo-controlled trial. The Lancet, 370(9598), pp.1560–1567. Available at: <http://linkinghub.elsevier.com/retrieve/pii/S01406736076130631>
