# Supplementary material for: Docosahexaenoic acid for reading, working memory and behavior in UK children aged 7-9: A randomized controlled trial for replication (the DOLAB II study)
Source: PLoS One. 2018 Feb 20;13(2):e0192909. doi: 10.1371/journal.pone.0192909 (PMC5819802; doi:10.1371/journal.pone.0192909)
Supplement: S6 File — (DOCX) [file pone.0192909.s006.docx]

## S6 – Blood Fatty Acid Data: Methods for capillary whole blood fatty acid analysis

Capillary whole blood from a finger stick was analyzed for total lipid fatty acids. A small lancet device (BD Microtainer Contact-Activated Lancets or similar, Emergency Medical Products) was applied to a pupil’s finger and drops of blood were collected on a 1.5 x 1.5 cm piece of filter paper (Whatman 3MM chromatography paper, Whatman Inc.) impregnated with BHT and prepared according to the methods of Ichihara et al. (1). The sample was dried at room temperature overnight and analyzed within 7 days of the blood collection. The blood saturated filter paper samples were directly methylated without a prior extraction step. Tricosanoic free fatty acid (23:0) (NuCheck Prep Elysian, MN, U.S.A) was added to each sample as an internal standard. The total lipids were saponified with 0.5 N methanolic sodium hydroxide and the fatty acids were converted to methyl esters with 14% BF_3_/methanol (Sigma) at 100°C for 30 minutes (Morrison and Smith (2)). The methyl esters were extracted with hexane and washed with saturated sodium chloride distilled water. Butylated hydroxytoluene was added before saponification and all samples were purged with N_2_ throughout the process to minimize oxidation. Fatty acid methyl esters were analyzed by gas-liquid-chromatography using a Hewlett Packard 6890 detector equipped with a flame ionization. The fatty acid methyl esters were separated on a 30 meter FAMEWAX capillary column (Restek, Bellefonte, PA; 0.25 mm diameter, 0.25 μm coating thickness) using hydrogen at a flow rate of 2.1 mL/min with a split ratio of 20:1. The chromatographic run parameters included an oven starting temperature of 130°C that was increased at 6°C/min to 225°C, where it was held for 20 minutes before increasing to 250°C at 15°C/min, with a final hold of 5 minutes. The injector and detector temperatures were constant at 220°C and 230°C, respectively. Peaks were identified by comparison of retention times with external fatty acid methyl ester standard mixtures from NuCheck Prep (Elysian, MN, U.S.A). The fatty acid profiles were expressed as a percent of the total μg of fatty acid (weight percent).

*See also*: Montgomery, P. et al., 2013. Low Blood Long Chain Omega-3 Fatty Acids in UK Children Are Associated with Poor Cognitive Performance and Behavior: A Cross-Sectional Analysis from the DOLAB Study. PLoS ONE, 8(6),

<https://doi.org/10.1371/journal.pone.0066697>.

*References*

(1) [Ichihar K](http://www.ncbi.nlm.nih.gov/pubmed?term=Ichihar%20K%5BAuthor%5D&cauthor=true&cauthor_uid=12056596), [Waku K](http://www.ncbi.nlm.nih.gov/pubmed?term=Waku%20K%5BAuthor%5D&cauthor=true&cauthor_uid=12056596), [Yamaguchi C](http://www.ncbi.nlm.nih.gov/pubmed?term=Yamaguchi%20C%5BAuthor%5D&cauthor=true&cauthor_uid=12056596), [Saito K](http://www.ncbi.nlm.nih.gov/pubmed?term=Saito%20K%5BAuthor%5D&cauthor=true&cauthor_uid=12056596), [Shibahara A](http://www.ncbi.nlm.nih.gov/pubmed?term=Shibahara%20A%5BAuthor%5D&cauthor=true&cauthor_uid=12056596), [Miyatani S](http://www.ncbi.nlm.nih.gov/pubmed?term=Miyatani%20S%5BAuthor%5D&cauthor=true&cauthor_uid=12056596), [Yamamoto K](http://www.ncbi.nlm.nih.gov/pubmed?term=Yamamoto%20K%5BAuthor%5D&cauthor=true&cauthor_uid=12056596). (2002) A Convenient Method for Determination of C20-22 PUFA Composition of Glycerolipids in Blood and Breast Milk. Lipids 37:523-526.

(2) Morrison, WR.; Smith LM. (1964) Preparation of fatty acid methyl esters and dimethylacetals from lipids with boron fluoride–methanol. J. Lipid Res. 5: 600-608.
