# Supplementary material for: Docosahexaenoic acid for reading, working memory and behavior in UK children aged 7-9: A randomized controlled trial for replication (the DOLAB II study)
Source: PLoS One. 2018 Feb 20;13(2):e0192909. doi: 10.1371/journal.pone.0192909 (PMC5819802; doi:10.1371/journal.pone.0192909)
Supplement: S7 File — (DOCX) [file pone.0192909.s007.docx]

## S7 – Randomization technical details

Specifications as per Sealed Envelope Ltd (<https://www.sealedenvelope.com/>):

**Treatment groups:** Active, Placebo

**Allocation ratio:** 1:1

**Balancing factors:** gender, school

**Method:** Minimisation with 30% chance of simple random allocation. This is equivalent to randomising to the treatment that would provide the most balance between treatment groups with probability 0.85. Siblings of previously randomised pupils were allocated to the same treatment group as their sibling. Forced randomisations and duplicates were not allowed.
