# Supplementary material for: Docosahexaenoic acid for reading, working memory and behavior in UK children aged 7-9: A randomized controlled trial for replication (the DOLAB II study)
Source: PLoS One. 2018 Feb 20;13(2):e0192909. doi: 10.1371/journal.pone.0192909 (PMC5819802; doi:10.1371/journal.pone.0192909)

## S8 – Prediction of Learning and Behaviour from DHA omega-3 change scores

The DOLAB II study used objective blood fatty acid measures to estimate adherence to the intervention. These also allow examination of changes in blood DHA levels to predict learning and behaviour outcomes. The relationship of the changes in DHA levels with the primary outcomes is assessed below using scatterplots with non-parametric (LOWESS) curves to visually inspect any relationship. – For reading and working memory an upward curve indicates positive relationship between changes in DHA levels and the outcomes, whilst downward curves for the behavioural outcomes indicate improvements in behaviour i.e. a reduction in behavioural problems.

### Reading


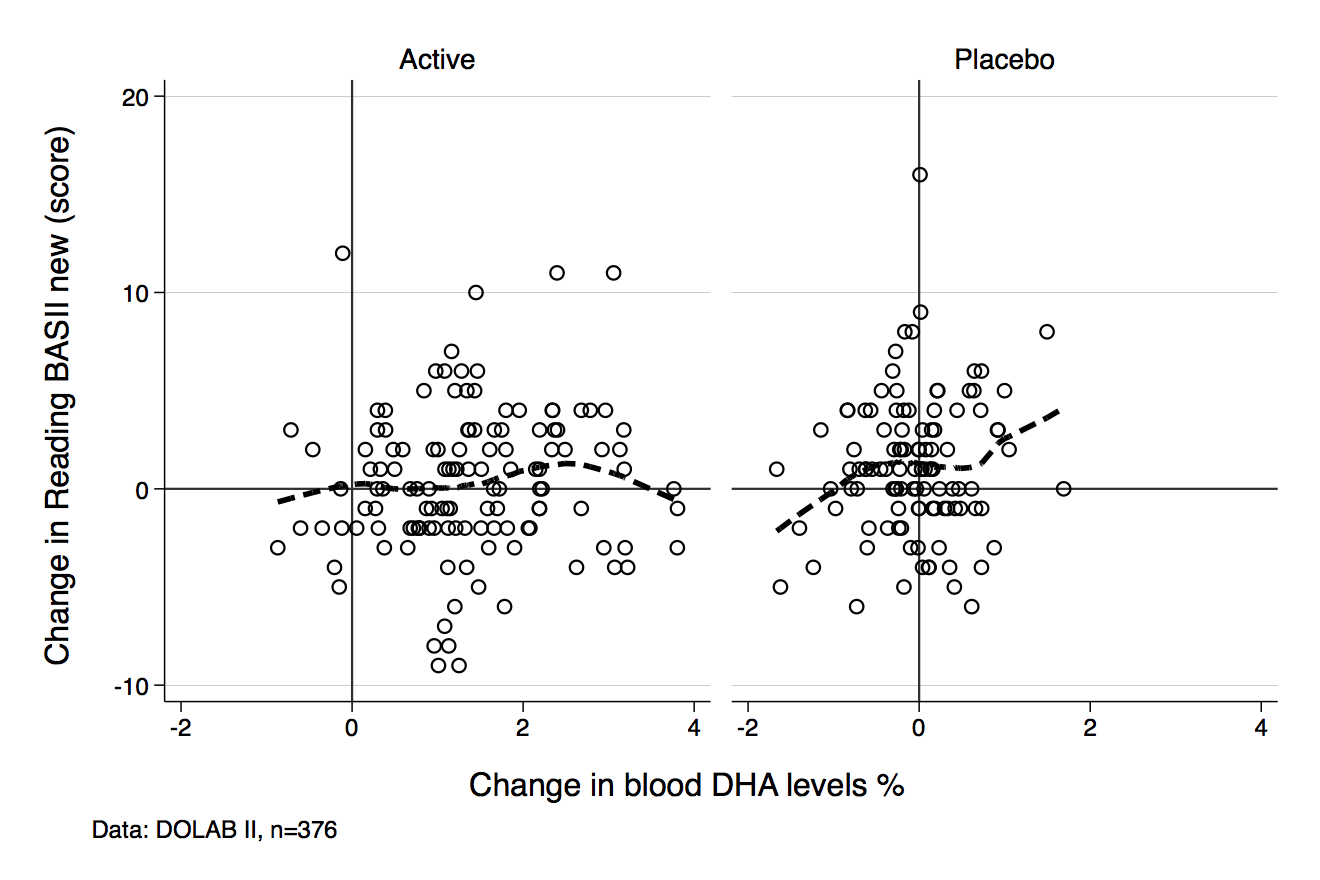


### Working Memory


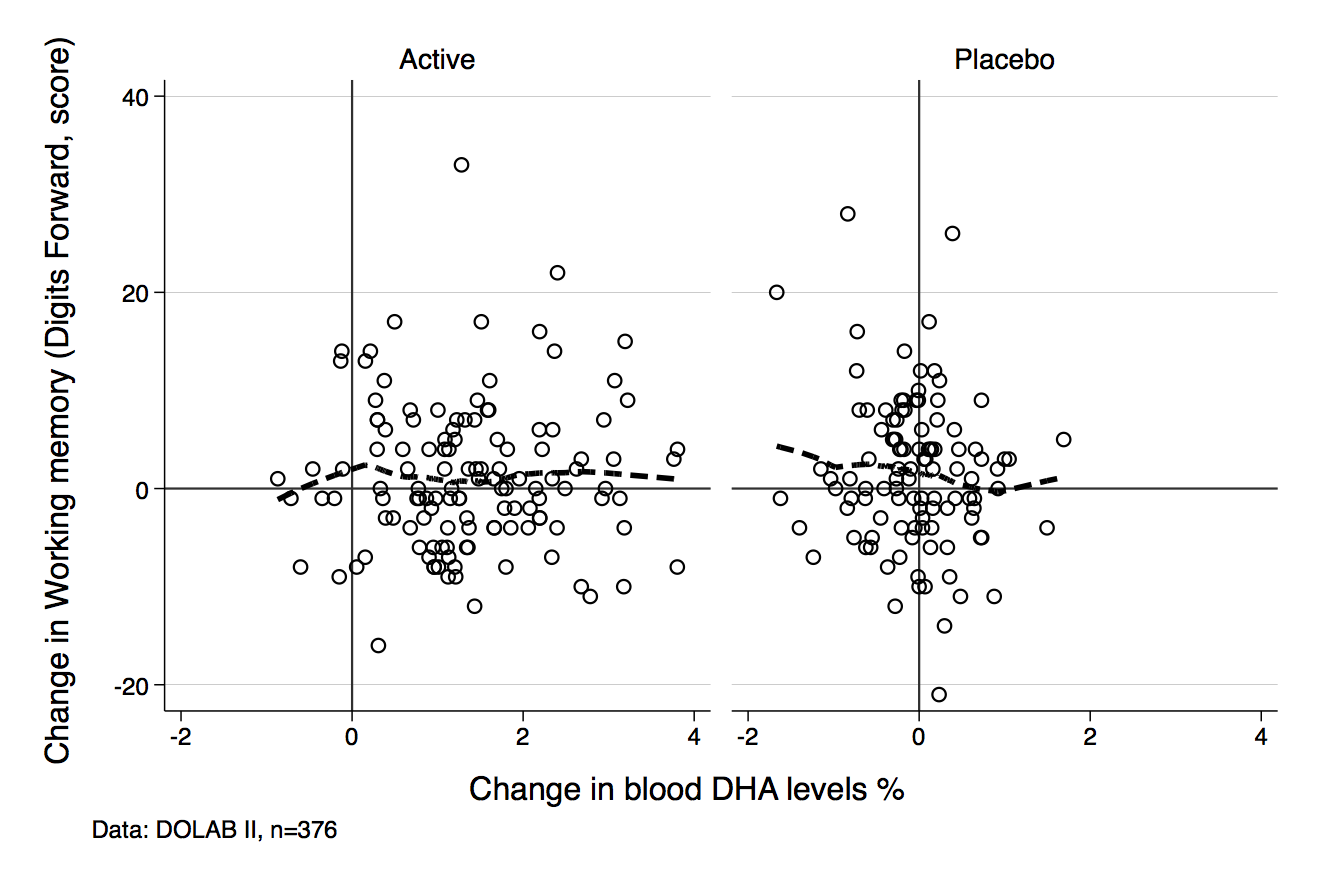


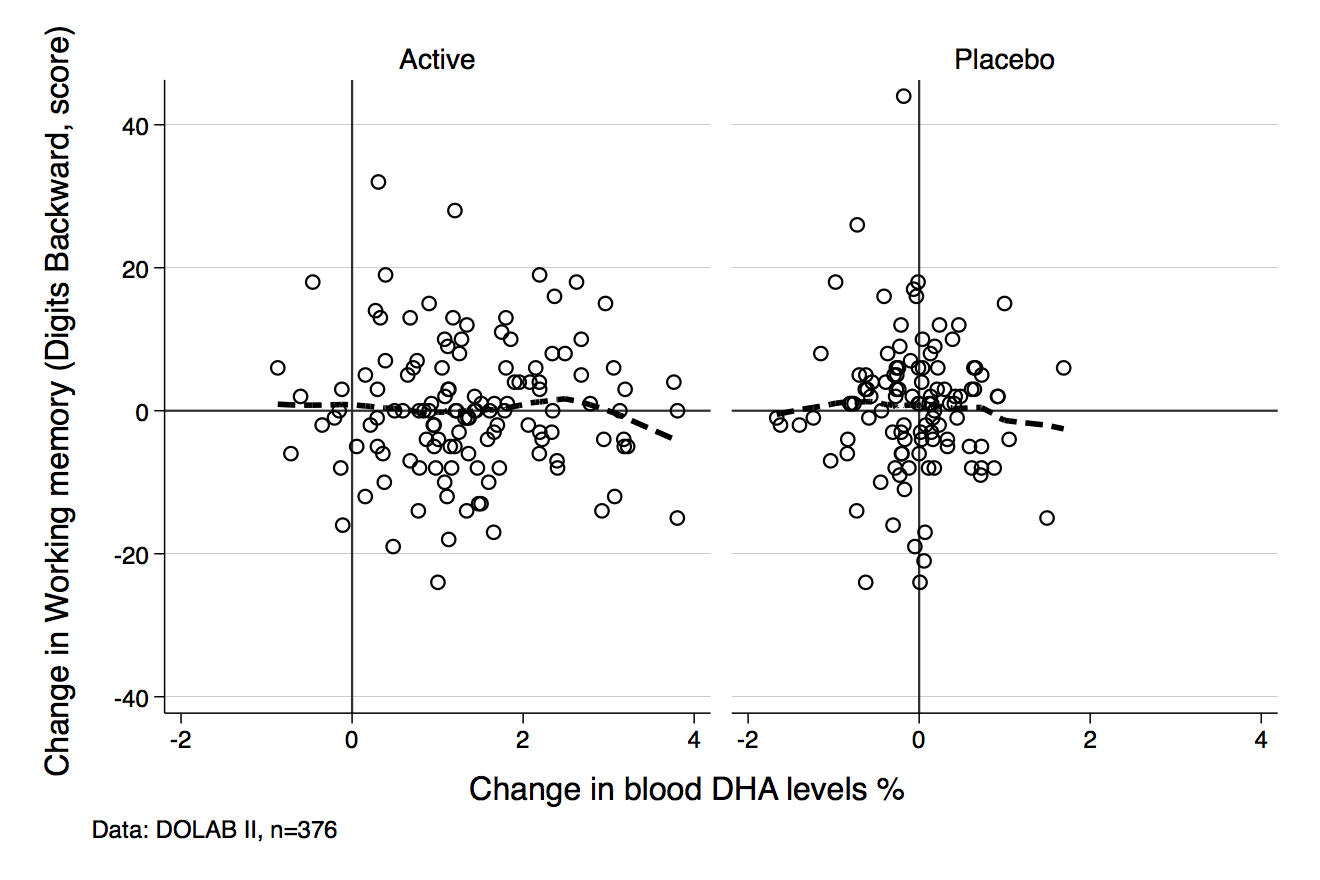


### Change score: Parent Oppositional T score


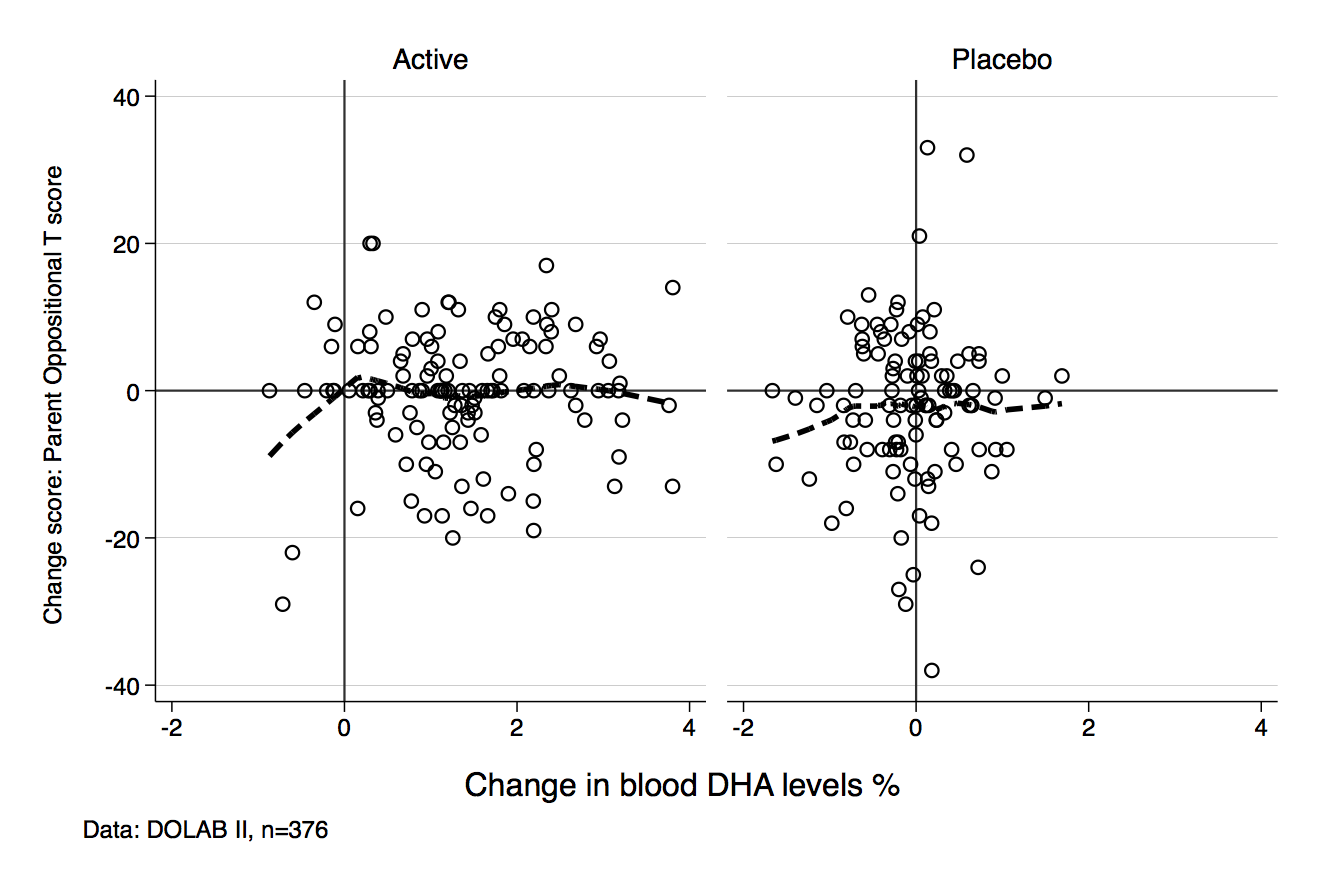


### Change score: Parent Cognitive Problems/Inattention T score


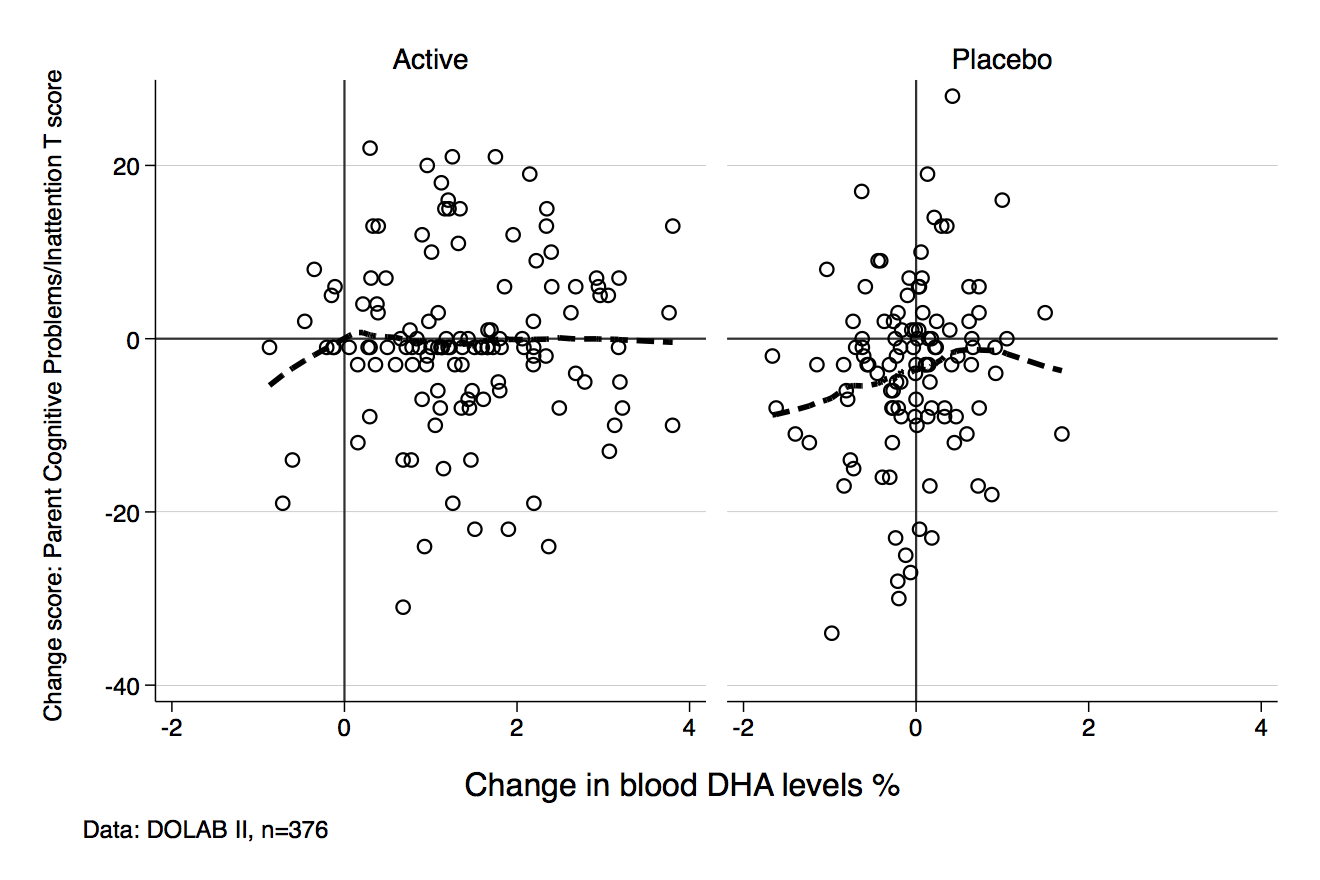


### Change score: Parent Hyperactivity T score


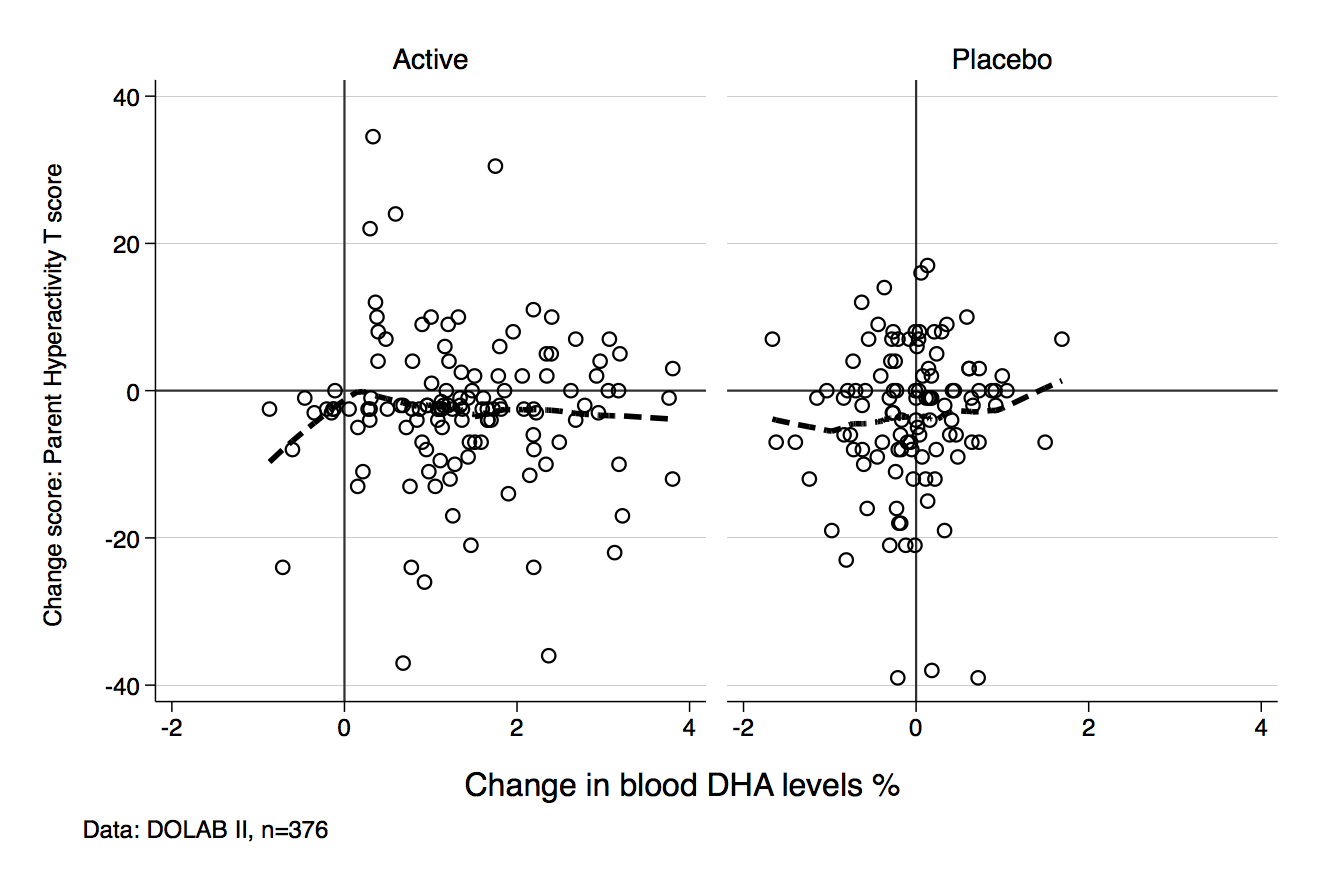


### Change score: Parent Anxious-Shy T score


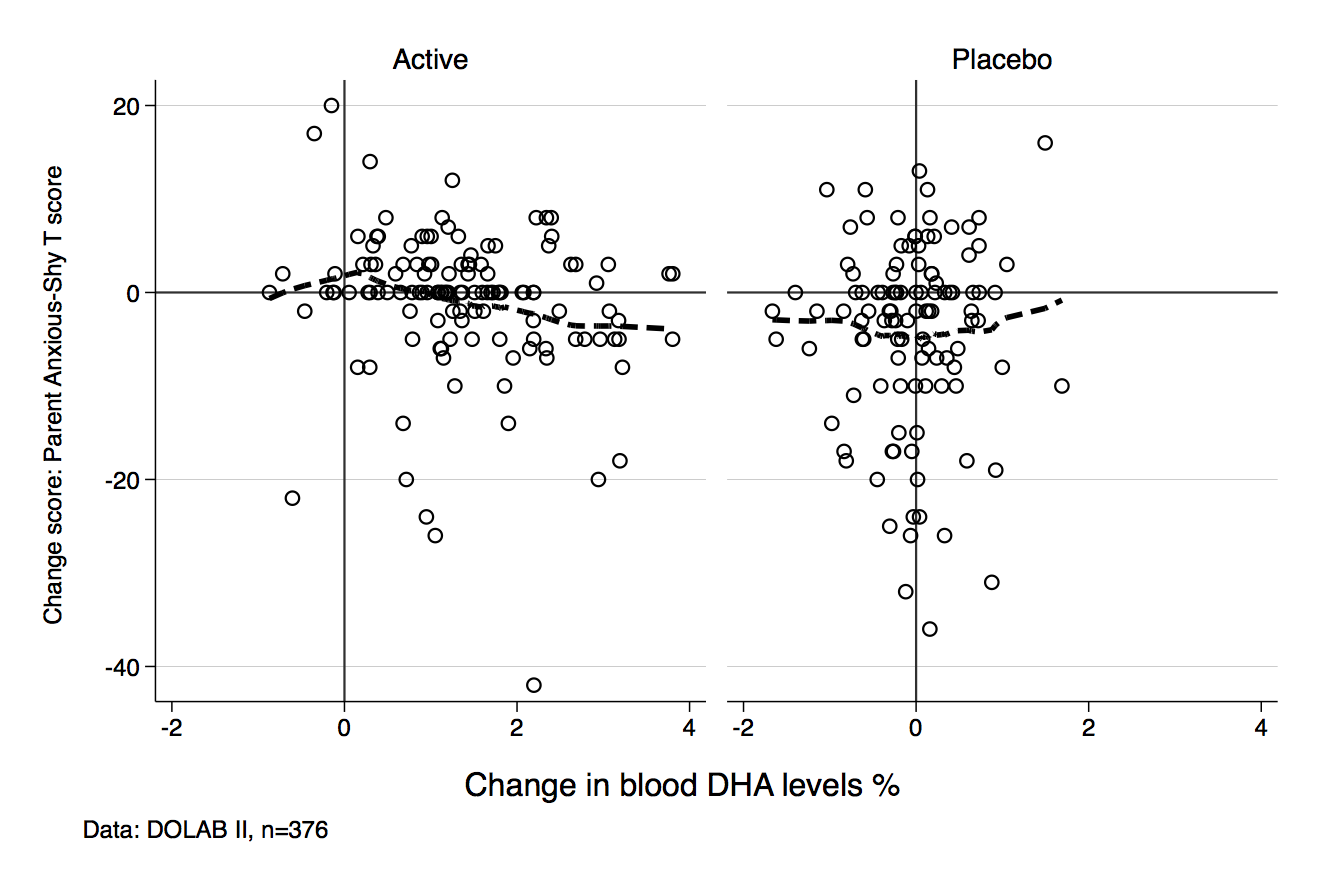


### Change score: Parent Perfectionism T score


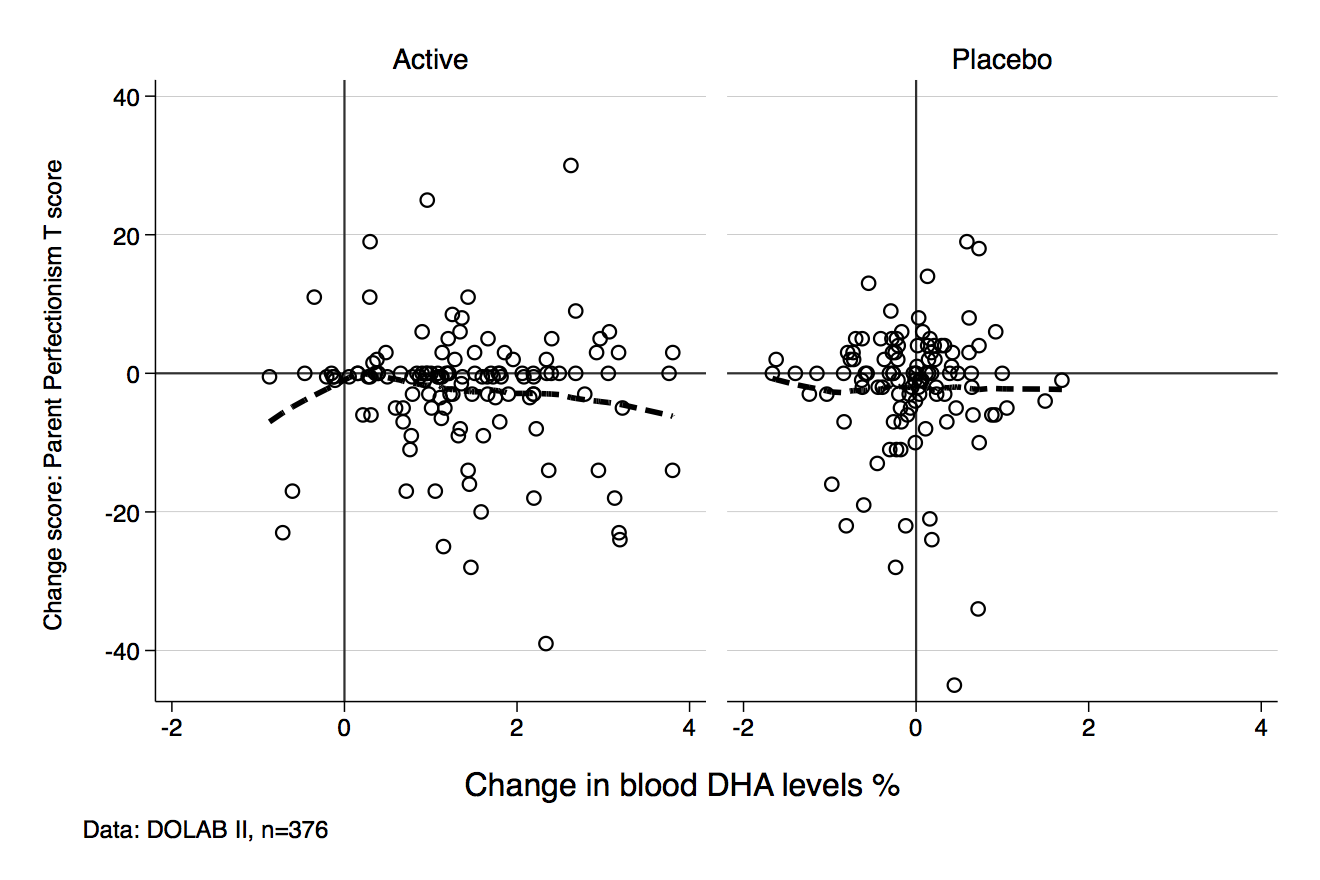


### Change score: Parent Social Problems T score


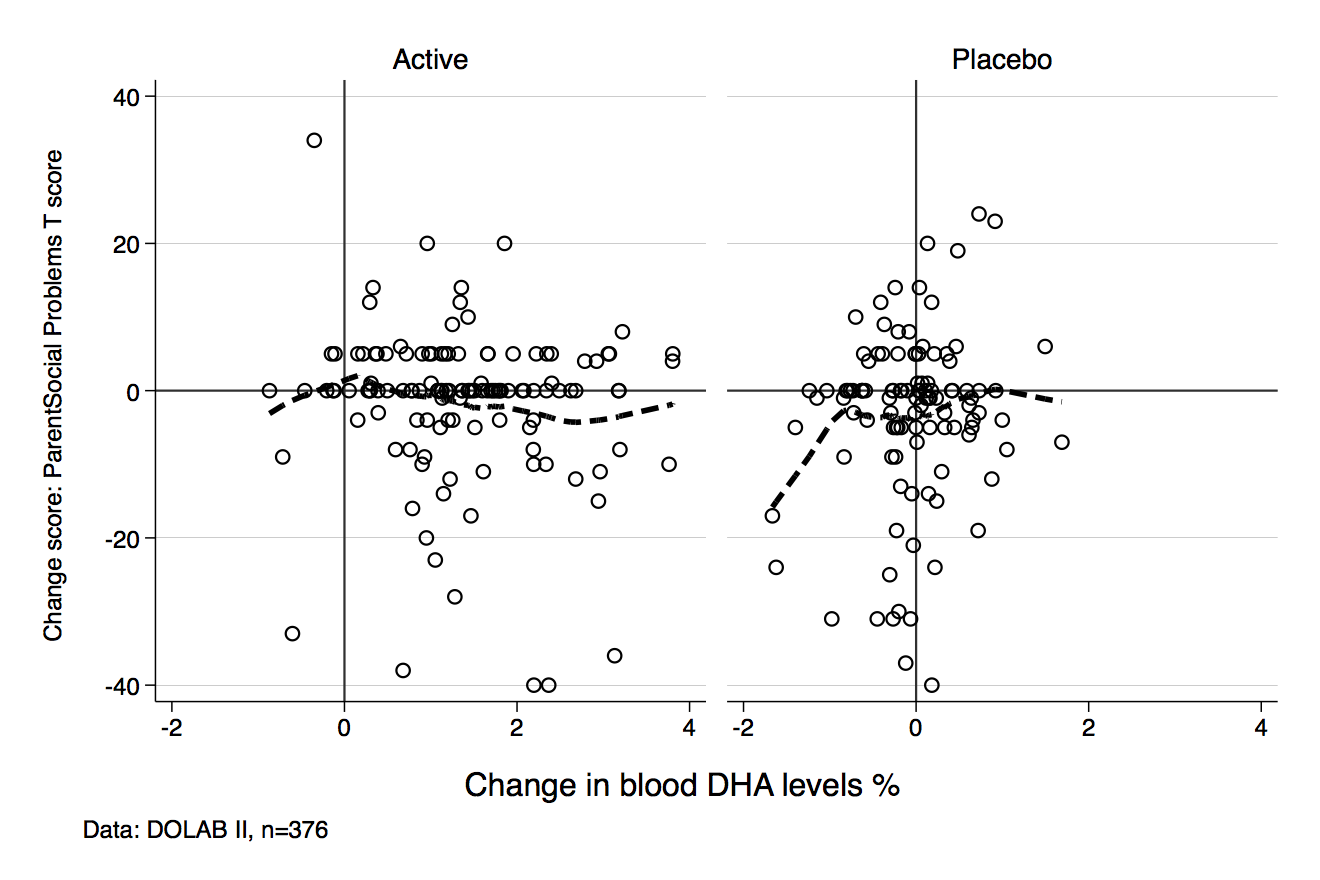


### Change score: Parent Psychosomatic T score


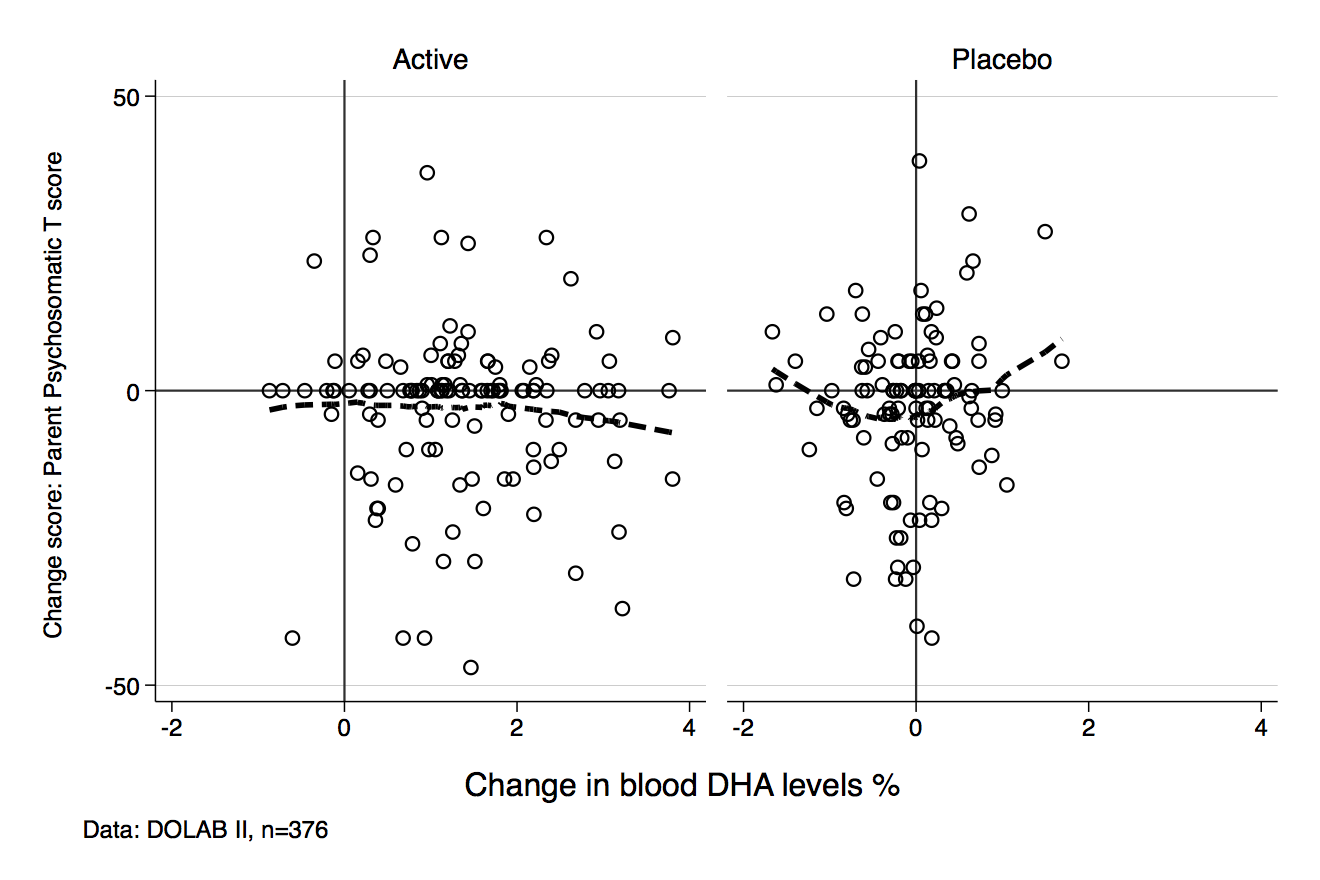


### Change score: Parent ADHD Index T score


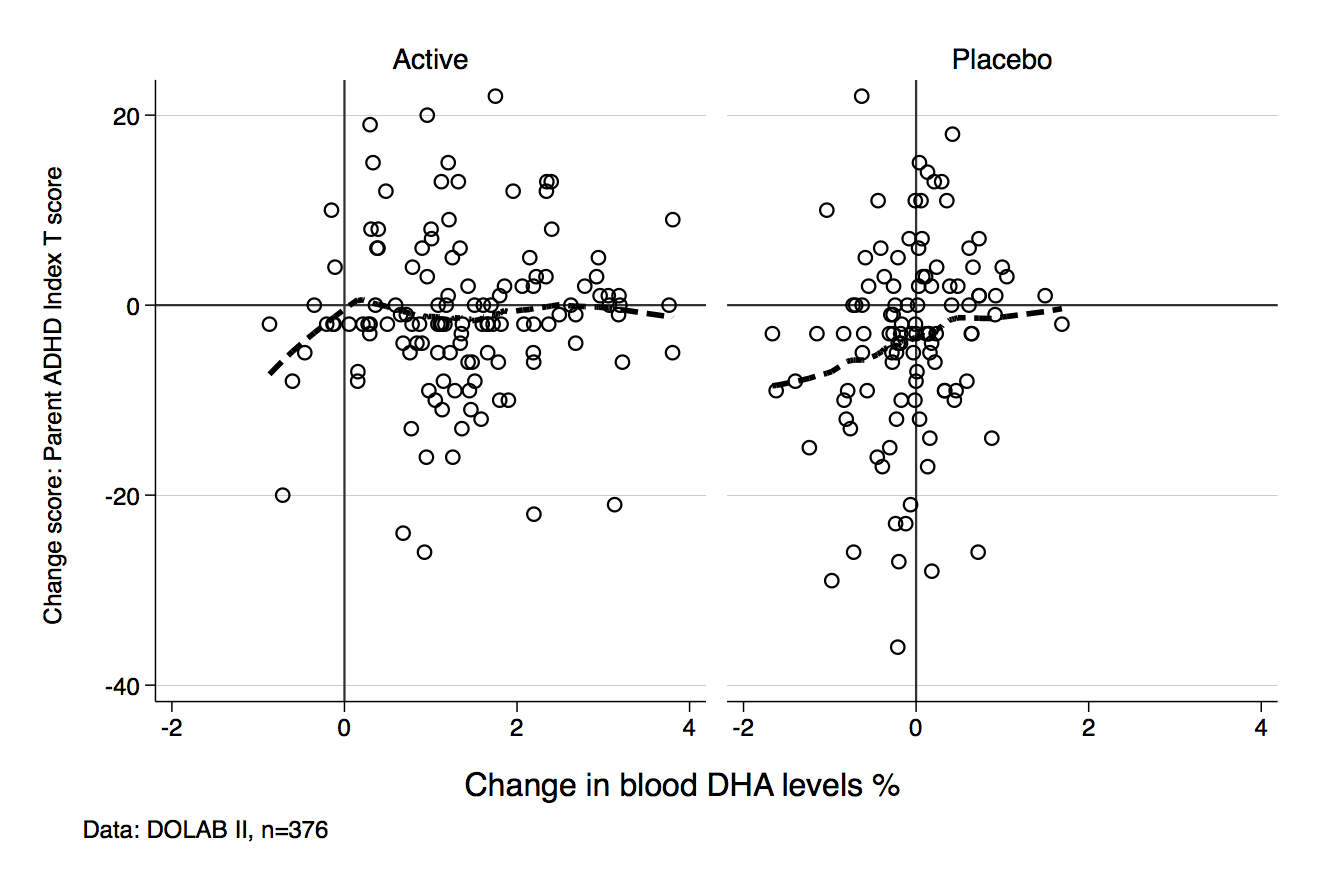


### Change score: Parent Global Index Restless-Impulsive T score


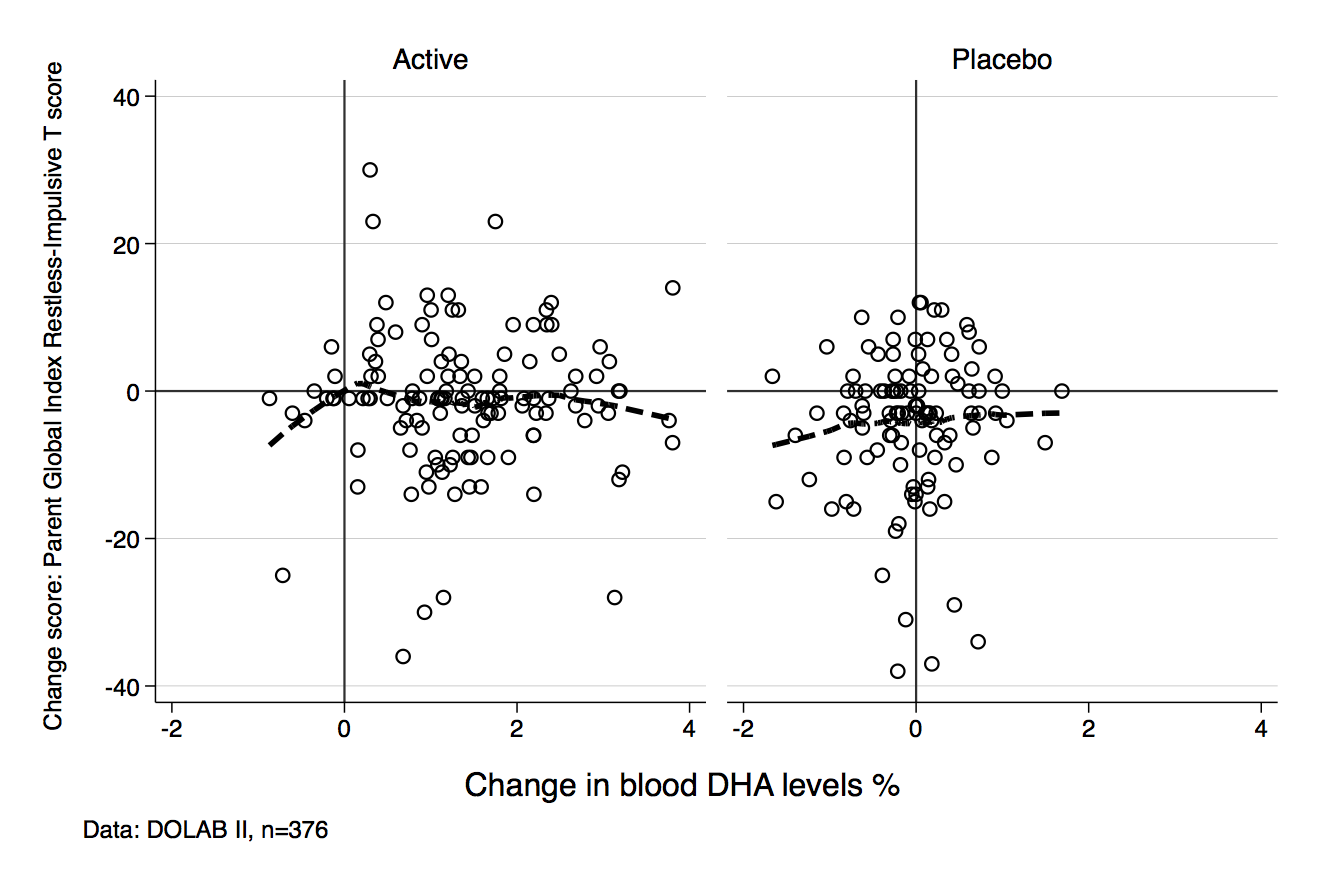


### Change score: Parent Global Index Emotional Lability T score


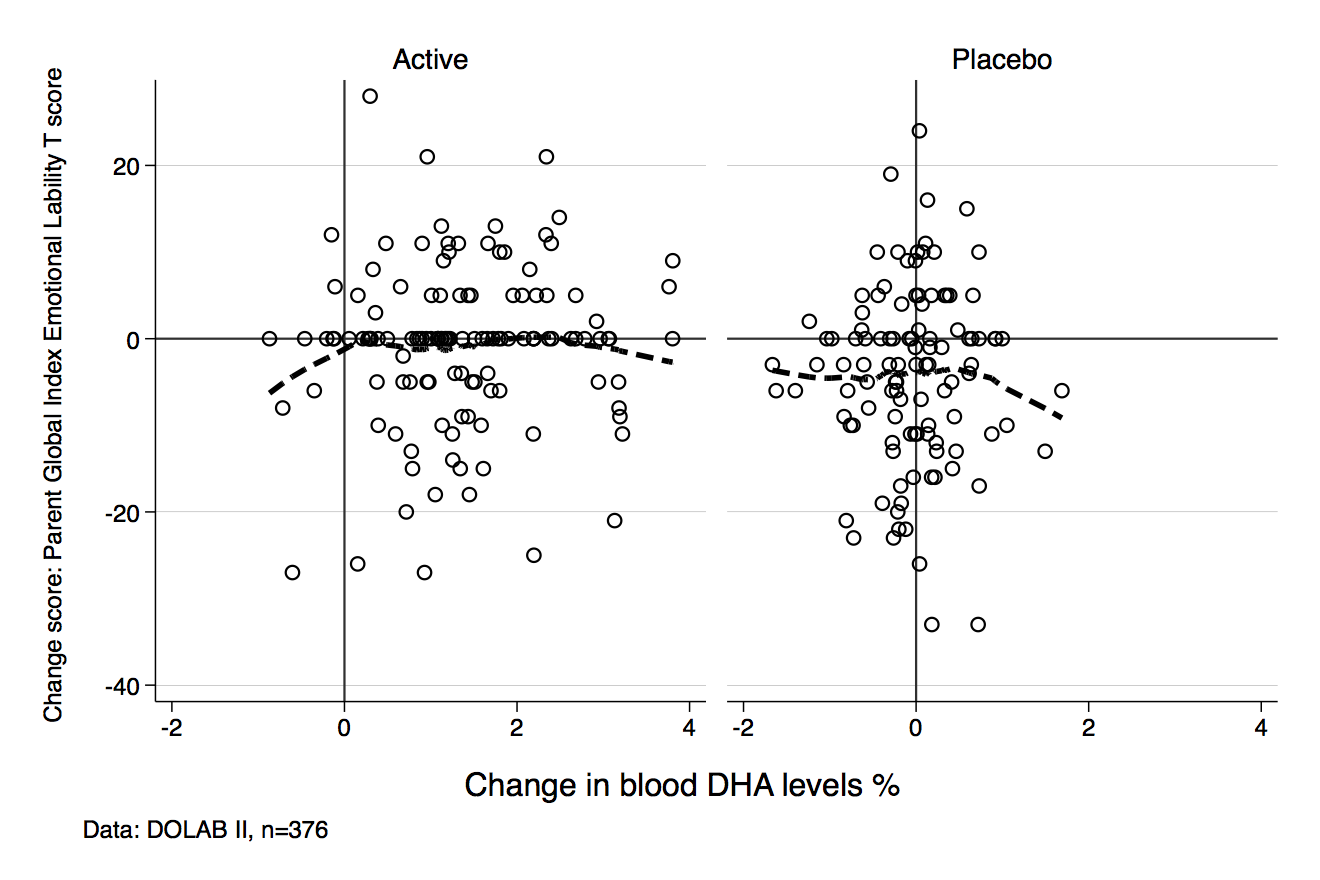


### Change score: Parent Global Index Total T score


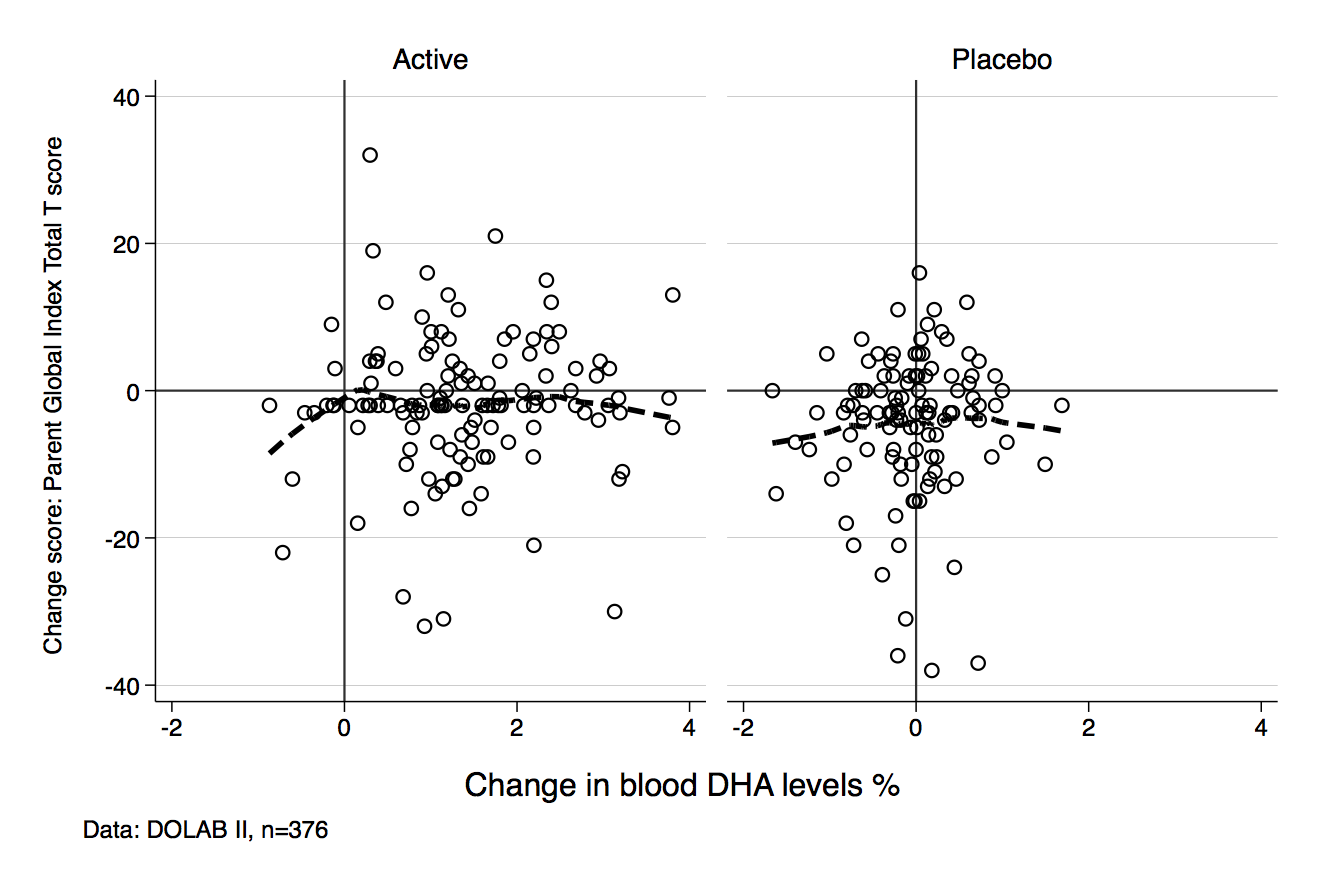


### Change score: Parent DSM-IV Inattentive T score


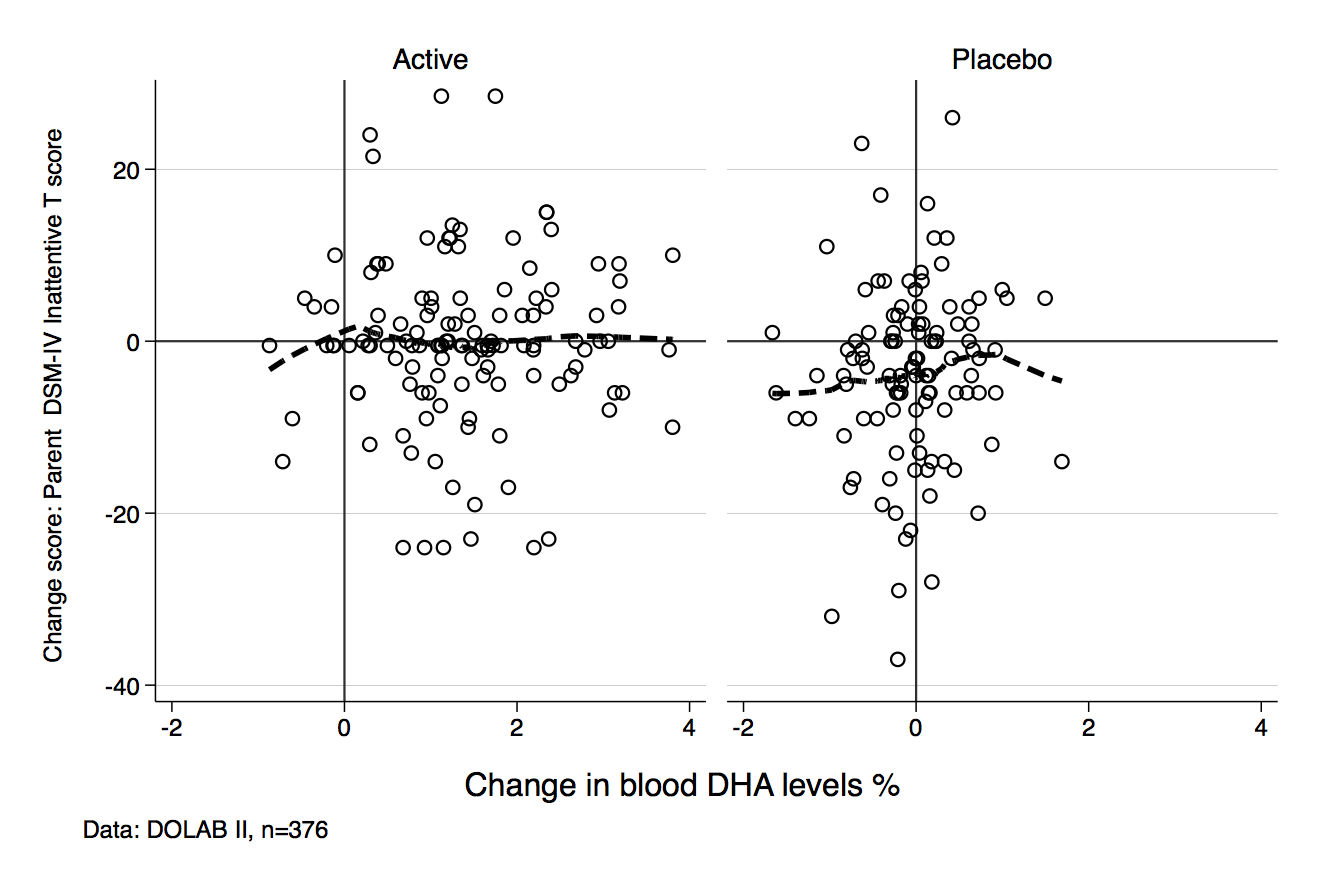


### Change score: Parent DSM-IV Hyperactive-Impulsive T score


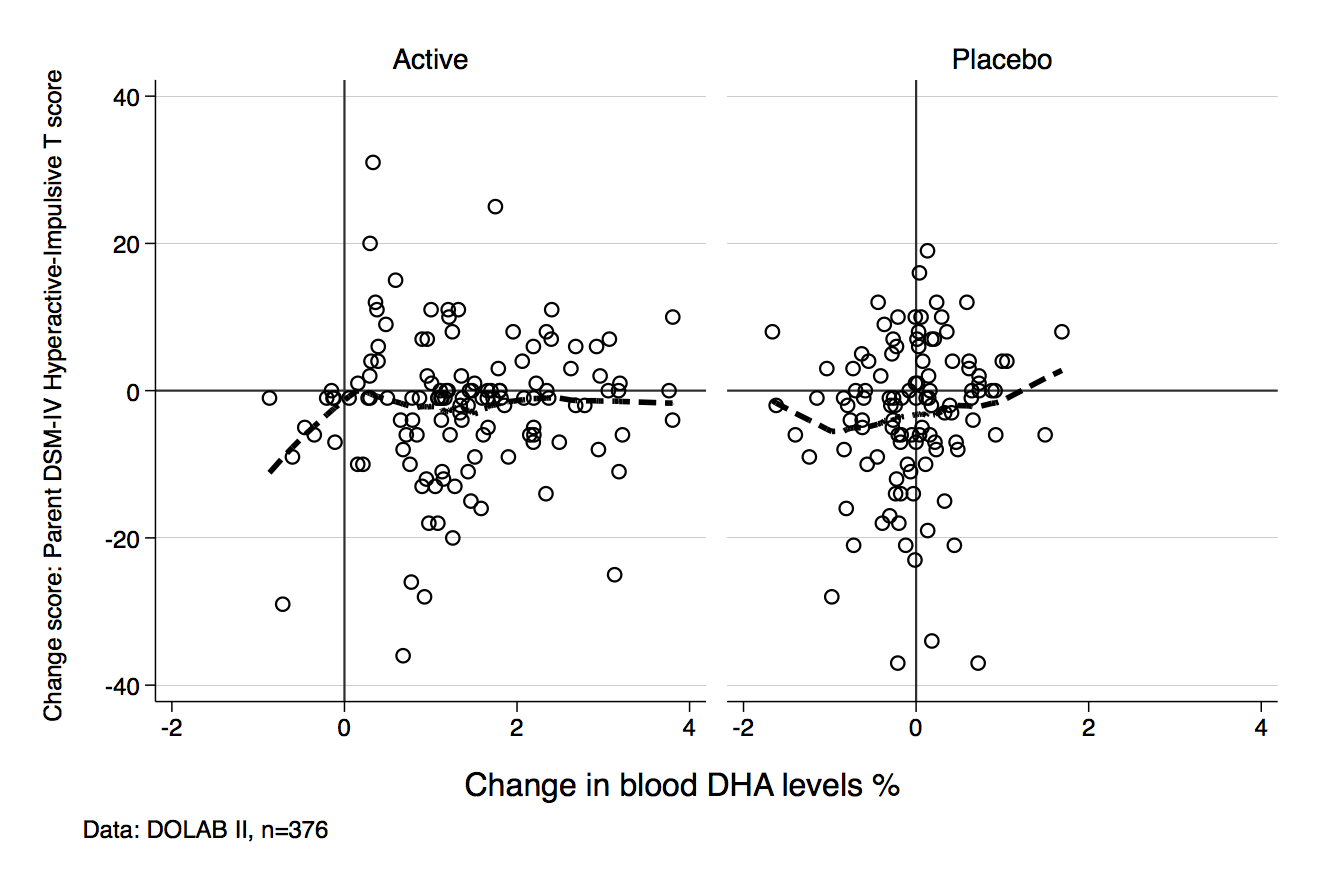


### Change score: Baseline Parent DSM-IV Total T score


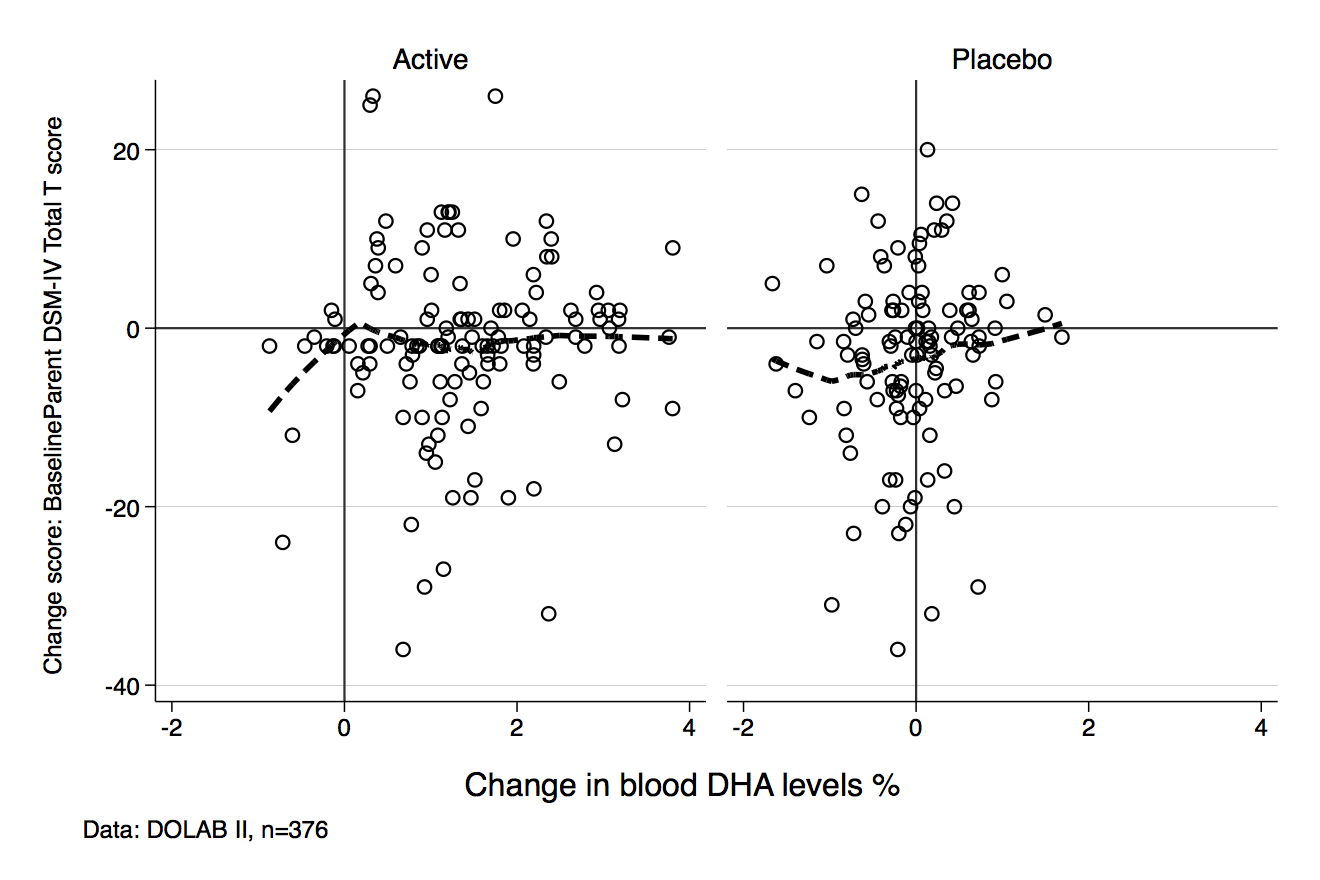

Supplement: S8 File — (DOCX) [file pone.0192909.s008.docx]
