# Supplementary material for: Docosahexaenoic acid for reading, working memory and behavior in UK children aged 7-9: A randomized controlled trial for replication (the DOLAB II study)
Source: PLoS One. 2018 Feb 20;13(2):e0192909. doi: 10.1371/journal.pone.0192909 (PMC5819802; doi:10.1371/journal.pone.0192909)
Supplement: S1 Table — (DOCX) [file pone.0192909.s009.docx]

Supplement 8 – Robustness check multivariate (OLS) analyses

The following tables present a more efficient analysis of the trial using multivariate (OLS) regressions of baseline scores and a treatment dummy on post-intervention outcome scores.

In addition, as a further robustness check against any influence from the minimization factors a second set of analyses with a gender dummy and participants’ school (as dummy) was run and is reported alongside the first basic regression (reported as Model 2).

## Table 1: New calibration BASII Reading (standard score)

|  | **Model_1 std. β** | **SE** | **t** | **p** | **95%** | **CI** | **Model_2 std. β** | **SE** | **t** | **p** | **95%** | **CI** |
| --- | --- | --- | --- | --- | --- | --- | --- | --- | --- | --- | --- | --- |
| Baseline | 0.867 | 0.028 | 30.454 | <0.001 | 0.811 | 0.923 | 0.865 | 0.035 | 25.012 | <0.001 | 0.797 | 0.934 |
| Active | 0.085 | 0.367 | 0.231 | 0.817 | -0.637 | 0.806 | 0.285 | 0.397 | 0.717 | 0.474 | -0.497 | 1.067 |
| Female |  |  |  |  |  |  | -0.589 | 0.464 | -1.268 | 0.206 | -1.503 | 0.325 |
| School Fixed Effects |  |  |  |  |  |  | Removed for presentation |  |  |  |  |  |
| Constant | 11.271 | 2.292 | 4.917 | <0.001 | 6.764 | 15.779 | 12.485 | 2.856 | 4.371 | <0.001 | 6.862 | 18.107 |
| N | 376 |  |  |  |  |  | 376 |  |  |  |  |  |
| r2 | 0.710 |  |  |  |  |  | 0.770 |  |  |  |  |  |
| r2 adj. | 0.710 |  |  |  |  |  | 0.700 |  |  |  |  |  |

## Table 2: BAS Working Memory FORWARD (t-score)

|  | **Model_1 std. β** | **SE** | **t** | **p** | **95%** | **CI** | **Model_2 std. β** | **SE** | **t** | **p** | **95%** | **CI** |
| --- | --- | --- | --- | --- | --- | --- | --- | --- | --- | --- | --- | --- |
| Baseline | 0.689 | 0.043 | 16.208 | <0.001 | 0.605 | 0.772 | 0.680 | 0.048 | 14.203 | <0.001 | 0.586 | 0.775 |
| Active | -0.588 | 0.732 | -0.803 | 0.423 | -2.028 | 0.852 | -1.171 | 0.777 | -1.507 | 0.133 | -2.700 | 0.358 |
| Female |  |  |  |  |  |  | 0.099 | 0.897 | 0.110 | 0.913 | -1.667 | 1.865 |
| School Fixed Effects |  |  |  |  |  |  | Removed for presentation |  |  |  |  |  |
| Constant | 14.287 | 1.893 | 7.546 | <0.001 | 10.564 | 18.010 | 16.365 | 2.933 | 5.579 | <0.001 | 10.592 | 22.138 |
| N | 376 |  |  |  |  |  | 376 |  |  |  |  |  |
| r2 | 0.420 |  |  |  |  |  | 0.550 |  |  |  |  |  |
| r2 adj. | 0.420 |  |  |  |  |  | 0.420 |  |  |  |  |  |

## Table 3: BAS Working Memory BACKWARD (t-score)

|  | **Model_1 std. β** | **SE** | **t** | **p** | **95%** | **CI** | **Model_2 std. β** | **SE** | **t** | **p** | **95%** | **CI** |
| --- | --- | --- | --- | --- | --- | --- | --- | --- | --- | --- | --- | --- |
| Baseline | 0.363 | 0.049 | 7.480 | <0.001 | 0.267 | 0.458 | 0.337 | 0.052 | 6.458 | <0.001 | 0.235 | 0.440 |
| Active | -1.357 | 0.818 | -1.659 | 0.098 | -2.967 | 0.252 | -1.618 | 0.808 | -2.002 | 0.046 | -3.208 | -0.027 |
| Female |  |  |  |  |  |  | 1.105 | 0.944 | 1.170 | 0.243 | -0.754 | 2.963 |
| School Fixed Effects |  |  |  |  |  |  | Removed for presentation |  |  |  |  |  |
| Constant | 27.990 | 2.172 | 12.890 | <0.001 | 23.720 | 32.260 | 29.024 | 3.134 | 9.261 | <0.001 | 22.856 | 35.192 |
| N | 374 |  |  |  |  |  | 374 |  |  |  |  |  |
| r2 | 0.140 |  |  |  |  |  | 0.420 |  |  |  |  |  |
| r2 adj. | 0.130 |  |  |  |  |  | 0.250 |  |  |  |  |  |

## Table 4: Parent Oppositional T-score

|  | **Model_1 std. β** | **SE** | **t** | **p** | **95%** | **CI** | **Model_2 std. β** | **SE** | **t** | **p** | **95%** | **CI** |
| --- | --- | --- | --- | --- | --- | --- | --- | --- | --- | --- | --- | --- |
| Baseline | 0.411 | 0.034 | 12.170 | <0.001 | 0.345 | 0.478 | 0.407 | 0.042 | 9.776 | <0.001 | 0.325 | 0.489 |
| Active | -0.584 | 0.765 | -0.763 | 0.446 | -2.088 | 0.920 | -0.595 | 0.857 | -0.694 | 0.489 | -2.282 | 1.093 |
| Female |  |  |  |  |  |  | -0.746 | 1.006 | -0.742 | 0.459 | -2.725 | 1.233 |
| School Fixed Effects |  |  |  |  |  |  | Removed for presentation |  |  |  |  |  |
| Constant | 31.106 | 1.923 | 16.175 | <0.001 | 27.325 | 34.888 | 32.948 | 3.404 | 9.679 | <0.001 | 26.248 | 39.648 |
| N | 376 |  |  |  |  |  | 376 |  |  |  |  |  |
| r2 | 0.280 |  |  |  |  |  | 0.380 |  |  |  |  |  |
| r2 adj. | 0.280 |  |  |  |  |  | 0.200 |  |  |  |  |  |

## Table 5: Parent Cognitive Problems/Inattention T-score

|  | **Model_1 std. β** | **SE** | **t** | **p** | **95%** | **CI** | **Model_2 std. β** | **SE** | **t** | **p** | **95%** | **CI** |
| --- | --- | --- | --- | --- | --- | --- | --- | --- | --- | --- | --- | --- |
| Baseline | 0.404 | 0.035 | 11.503 | <0.001 | 0.335 | 0.473 | 0.395 | 0.041 | 9.563 | <0.001 | 0.314 | 0.477 |
| Active | -1.685 | 0.734 | -2.297 | 0.022 | -3.127 | -0.242 | -2.010 | 0.776 | -2.591 | 0.010 | -3.536 | -0.483 |
| Female |  |  |  |  |  |  | 0.129 | 0.907 | 0.142 | 0.887 | -1.656 | 1.914 |
| School Fixed Effects |  |  |  |  |  |  | Removed for presentation |  |  |  |  |  |
| Constant | 34.817 | 2.179 | 15.977 | <0.001 | 30.532 | 39.102 | 34.227 | 3.255 | 10.516 | <0.001 | 27.821 | 40.634 |
| N | 376 |  |  |  |  |  | 376 |  |  |  |  |  |
| r2 | 0.270 |  |  |  |  |  | 0.440 |  |  |  |  |  |
| r2 adj. | 0.260 |  |  |  |  |  | 0.270 |  |  |  |  |  |

## Table 6: Parent Hyperactivity T-score

|  | **Model_1 std. β** | **SE** | **t** | **p** | **95%** | **CI** | **Model_2 std. β** | **SE** | **t** | **p** | **95%** | **CI** |
| --- | --- | --- | --- | --- | --- | --- | --- | --- | --- | --- | --- | --- |
| Baseline | 0.439 | 0.032 | 13.645 | <0.001 | 0.376 | 0.503 | 0.447 | 0.038 | 11.894 | <0.001 | 0.373 | 0.521 |
| Active | -0.606 | 0.764 | -0.793 | 0.428 | -2.108 | 0.896 | -0.572 | 0.815 | -0.702 | 0.483 | -2.177 | 1.032 |
| Female |  |  |  |  |  |  | -0.400 | 0.953 | -0.420 | 0.675 | -2.275 | 1.475 |
| School Fixed Effects |  |  |  |  |  |  | Removed for presentation |  |  |  |  |  |
| Constant | 29.207 | 1.920 | 15.213 | <0.001 | 25.432 | 32.982 | 26.149 | 3.193 | 8.191 | <0.001 | 19.866 | 32.433 |
| N | 376 |  |  |  |  |  | 376 |  |  |  |  |  |
| r2 | 0.340 |  |  |  |  |  | 0.480 |  |  |  |  |  |
| r2 adj. | 0.330 |  |  |  |  |  | 0.320 |  |  |  |  |  |

## Table 7: Parent Anxious-Shy T-score

|  | **Model_1 std. β** | **SE** | **t** | **p** | **95%** | **CI** | **Model_2 std. β** | **SE** | **t** | **p** | **95%** | **CI** |
| --- | --- | --- | --- | --- | --- | --- | --- | --- | --- | --- | --- | --- |
| Baseline | 0.411 | 0.029 | 14.024 | <0.001 | 0.354 | 0.469 | 0.405 | 0.034 | 12.072 | <0.001 | 0.339 | 0.471 |
| Active | -1.449 | 0.630 | -2.298 | 0.022 | -2.688 | -0.209 | -1.606 | 0.661 | -2.430 | 0.016 | -2.907 | -0.305 |
| Female |  |  |  |  |  |  | -1.076 | 0.766 | -1.404 | 0.161 | -2.584 | 0.432 |
| School Fixed Effects |  |  |  |  |  |  | Removed for presentation |  |  |  |  |  |
| Constant | 28.959 | 1.556 | 18.611 | <0.001 | 25.899 | 32.019 | 29.551 | 2.568 | 11.507 | <0.001 | 24.497 | 34.606 |
| N | 376 |  |  |  |  |  | 376 |  |  |  |  |  |
| r2 | 0.350 |  |  |  |  |  | 0.510 |  |  |  |  |  |
| r2 adj. | 0.340 |  |  |  |  |  | 0.360 |  |  |  |  |  |

## Table 8: Parent Perfectionism T-score

|  | **Model_1 std. β** | **SE** | **t** | **p** | **95%** | **CI** | **Model_2 std. β** | **SE** | **t** | **p** | **95%** | **CI** |
| --- | --- | --- | --- | --- | --- | --- | --- | --- | --- | --- | --- | --- |
| Baseline | 0.364 | 0.032 | 11.338 | <0.001 | 0.301 | 0.427 | 0.319 | 0.037 | 8.647 | <0.001 | 0.246 | 0.391 |
| Active | 0.137 | 0.670 | 0.205 | 0.838 | -1.180 | 1.455 | 0.110 | 0.702 | 0.156 | 0.876 | -1.272 | 1.492 |
| Female |  |  |  |  |  |  | -2.469 | 0.830 | -2.976 | 0.003 | -4.102 | -0.836 |
| School Fixed Effects |  |  |  |  |  |  | Removed for presentation |  |  |  |  |  |
| Constant | 29.443 | 1.673 | 17.600 | <0.001 | 26.153 | 32.732 | 31.634 | 2.703 | 11.704 | <0.001 | 26.314 | 36.953 |
| N | 376 |  |  |  |  |  | 376 |  |  |  |  |  |
| r2 | 0.260 |  |  |  |  |  | 0.440 |  |  |  |  |  |
| r2 adj. | 0.250 |  |  |  |  |  | 0.270 |  |  |  |  |  |

## Table 9: ParentSocial Problems T-score

|  | **Model_1 std. β** | **SE** | **t** | **p** | **95%** | **CI** | **Model_2 std. β** | **SE** | **t** | **p** | **95%** | **CI** |
| --- | --- | --- | --- | --- | --- | --- | --- | --- | --- | --- | --- | --- |
| Baseline | 0.388 | 0.033 | 11.759 | <0.001 | 0.323 | 0.453 | 0.422 | 0.039 | 10.699 | <0.001 | 0.345 | 0.500 |
| Active | -0.117 | 0.805 | -0.145 | 0.885 | -1.699 | 1.465 | -0.116 | 0.856 | -0.135 | 0.893 | -1.800 | 1.568 |
| Female |  |  |  |  |  |  | -1.382 | 0.999 | -1.384 | 0.167 | -3.347 | 0.584 |
| School Fixed Effects |  |  |  |  |  |  | Removed for presentation |  |  |  |  |  |
| Constant | 31.623 | 1.874 | 16.873 | <0.001 | 27.938 | 35.309 | 32.934 | 3.266 | 10.084 | <0.001 | 26.506 | 39.362 |
| N | 376 |  |  |  |  |  | 376 |  |  |  |  |  |
| r2 | 0.270 |  |  |  |  |  | 0.430 |  |  |  |  |  |
| r2 adj. | 0.270 |  |  |  |  |  | 0.270 |  |  |  |  |  |

## Table 10: Parent Psychosomatic T-score

|  | **Model_1 std. β** | **SE** | **t** | **p** | **95%** | **CI** | **Model_2 std. β** | **SE** | **t** | **p** | **95%** | **CI** |
| --- | --- | --- | --- | --- | --- | --- | --- | --- | --- | --- | --- | --- |
| Baseline | 0.283 | 0.041 | 6.826 | <0.001 | 0.202 | 0.365 | 0.298 | 0.047 | 6.351 | <0.001 | 0.206 | 0.391 |
| Active | -0.089 | 1.012 | -0.088 | 0.930 | -2.079 | 1.902 | -0.263 | 1.080 | -0.243 | 0.808 | -2.389 | 1.864 |
| Female |  |  |  |  |  |  | 0.381 | 1.260 | 0.302 | 0.762 | -2.100 | 2.862 |
| School Fixed Effects |  |  |  |  |  |  | Removed for presentation |  |  |  |  |  |
| Constant | 36.185 | 2.333 | 15.512 | <0.001 | 31.598 | 40.772 | 35.035 | 4.096 | 8.553 | <0.001 | 26.973 | 43.097 |
| N | 376 |  |  |  |  |  | 376 |  |  |  |  |  |
| r2 | 0.110 |  |  |  |  |  | 0.300 |  |  |  |  |  |
| r2 adj. | 0.110 |  |  |  |  |  | 0.090 |  |  |  |  |  |

## Table 11: Parent ADHD Index T-score

|  | **Model_1 std. β** | **SE** | **t** | **p** | **95%** | **CI** | **Model_2 std. β** | **SE** | **t** | **p** | **95%** | **CI** |
| --- | --- | --- | --- | --- | --- | --- | --- | --- | --- | --- | --- | --- |
| Baseline | 0.444 | 0.033 | 13.644 | <0.001 | 0.380 | 0.509 | 0.462 | 0.038 | 12.060 | <0.001 | 0.387 | 0.538 |
| Active | -0.956 | 0.689 | -1.388 | 0.166 | -2.310 | 0.399 | -1.237 | 0.736 | -1.681 | 0.094 | -2.685 | 0.211 |
| Female |  |  |  |  |  |  | -0.372 | 0.859 | -0.433 | 0.665 | -2.063 | 1.319 |
| School Fixed Effects |  |  |  |  |  |  | Removed for presentation |  |  |  |  |  |
| Constant | 30.410 | 1.956 | 15.548 | <0.001 | 26.564 | 34.256 | 27.386 | 3.066 | 8.932 | <0.001 | 21.352 | 33.420 |
| N | 376 |  |  |  |  |  | 376 |  |  |  |  |  |
| r2 | 0.340 |  |  |  |  |  | 0.480 |  |  |  |  |  |
| r2 adj. | 0.330 |  |  |  |  |  | 0.320 |  |  |  |  |  |

## Table 12: Parent Global Index Restless-Impulsive T-score

|  | **Model_1 std. β** | **SE** | **t** | **p** | **95%** | **CI** | **Model_2 std. β** | **SE** | **t** | **p** | **95%** | **CI** |
| --- | --- | --- | --- | --- | --- | --- | --- | --- | --- | --- | --- | --- |
| Baseline | 0.411 | 0.032 | 12.986 | <0.001 | 0.349 | 0.473 | 0.437 | 0.038 | 11.543 | <0.001 | 0.362 | 0.511 |
| Active | -1.055 | 0.722 | -1.461 | 0.145 | -2.475 | 0.365 | -1.139 | 0.777 | -1.465 | 0.144 | -2.669 | 0.391 |
| Female |  |  |  |  |  |  | -0.755 | 0.907 | -0.833 | 0.405 | -2.540 | 1.029 |
| School Fixed Effects |  |  |  |  |  |  | Removed for presentation |  |  |  |  |  |
| Constant | 31.423 | 1.885 | 16.669 | <0.001 | 27.716 | 35.130 | 28.607 | 3.078 | 9.294 | <0.001 | 22.549 | 34.666 |
| N | 376 |  |  |  |  |  | 376 |  |  |  |  |  |
| r2 | 0.320 |  |  |  |  |  | 0.450 |  |  |  |  |  |
| r2 adj. | 0.310 |  |  |  |  |  | 0.290 |  |  |  |  |  |

## Table 13: Parent Global Index Emotional Lability T-score

|  | **Model_1 std. β** | **SE** | **t** | **p** | **95%** | **CI** | **Model_2 std. β** | **SE** | **t** | **p** | **95%** | **CI** |
| --- | --- | --- | --- | --- | --- | --- | --- | --- | --- | --- | --- | --- |
| Baseline | 0.437 | 0.036 | 12.119 | <0.001 | 0.366 | 0.508 | 0.414 | 0.044 | 9.515 | <0.001 | 0.328 | 0.500 |
| Active | -1.836 | 0.779 | -2.357 | 0.019 | -3.368 | -0.304 | -2.038 | 0.854 | -2.386 | 0.018 | -3.720 | -0.357 |
| Female |  |  |  |  |  |  | -1.022 | 0.998 | -1.024 | 0.307 | -2.986 | 0.942 |
| School Fixed Effects |  |  |  |  |  |  | Removed for presentation |  |  |  |  |  |
| Constant | 29.691 | 2.034 | 14.597 | <0.001 | 25.692 | 33.691 | 32.398 | 3.485 | 9.296 | <0.001 | 25.538 | 39.257 |
| N | 376 |  |  |  |  |  | 376 |  |  |  |  |  |
| r2 | 0.290 |  |  |  |  |  | 0.410 |  |  |  |  |  |
| r2 adj. | 0.280 |  |  |  |  |  | 0.230 |  |  |  |  |  |

## Table 14: Parent Global Index Total T-score

|  | **Model_1 std. β** | **SE** | **t** | **p** | **95%** | **CI** | **Model_2 std. β** | **SE** | **t** | **p** | **95%** | **CI** |
| --- | --- | --- | --- | --- | --- | --- | --- | --- | --- | --- | --- | --- |
| Baseline | 0.417 | 0.032 | 13.067 | <0.001 | 0.354 | 0.480 | 0.434 | 0.039 | 11.270 | <0.001 | 0.358 | 0.510 |
| Active | -1.062 | 0.718 | -1.480 | 0.140 | -2.473 | 0.349 | -1.170 | 0.786 | -1.488 | 0.138 | -2.718 | 0.377 |
| Female |  |  |  |  |  |  | -0.854 | 0.917 | -0.931 | 0.353 | -2.659 | 0.951 |
| School Fixed Effects |  |  |  |  |  |  | Removed for presentation |  |  |  |  |  |
| Constant | 30.804 | 1.889 | 16.306 | <0.001 | 27.089 | 34.519 | 29.328 | 3.148 | 9.316 | <0.001 | 23.132 | 35.525 |
| N | 376 |  |  |  |  |  | 376 |  |  |  |  |  |
| r2 | 0.320 |  |  |  |  |  | 0.440 |  |  |  |  |  |
| r2 adj. | 0.310 |  |  |  |  |  | 0.270 |  |  |  |  |  |

## Table 15: Parent DSM-IV Inattentive T-score

|  | **Model_1 std. β** | **SE** | **t** | **p** | **95%** | **CI** | **Model_2 std. β** | **SE** | **t** | **p** | **95%** | **CI** |
| --- | --- | --- | --- | --- | --- | --- | --- | --- | --- | --- | --- | --- |
| Baseline | 0.436 | 0.036 | 12.084 | <0.001 | 0.365 | 0.506 | 0.447 | 0.042 | 10.605 | <0.001 | 0.364 | 0.530 |
| Active | -2.139 | 0.768 | -2.786 | 0.006 | -3.649 | -0.629 | -2.285 | 0.812 | -2.813 | 0.005 | -3.884 | -0.686 |
| Female |  |  |  |  |  |  | 0.327 | 0.949 | 0.345 | 0.731 | -1.540 | 2.194 |
| School Fixed Effects |  |  |  |  |  |  | Removed for presentation |  |  |  |  |  |
| Constant | 31.356 | 2.127 | 14.741 | <0.001 | 27.173 | 35.538 | 27.899 | 3.338 | 8.358 | <0.001 | 21.330 | 34.469 |
| N | 376 |  |  |  |  |  | 376 |  |  |  |  |  |
| r2 | 0.290 |  |  |  |  |  | 0.450 |  |  |  |  |  |
| r2 adj. | 0.290 |  |  |  |  |  | 0.290 |  |  |  |  |  |

## Table 16: Parent DSM-IV Hyperactive-Impulsive T-score

|  | **Model_1 std. β** | **SE** | **t** | **p** | **95%** | **CI** | **Model_2 std. β** | **SE** | **t** | **p** | **95%** | **CI** |
| --- | --- | --- | --- | --- | --- | --- | --- | --- | --- | --- | --- | --- |
| Baseline | 0.443 | 0.031 | 14.501 | <0.001 | 0.383 | 0.503 | 0.467 | 0.036 | 12.953 | <0.001 | 0.396 | 0.538 |
| Active | -0.418 | 0.723 | -0.578 | 0.563 | -1.839 | 1.003 | -0.328 | 0.777 | -0.422 | 0.673 | -1.858 | 1.202 |
| Female |  |  |  |  |  |  | -0.675 | 0.909 | -0.743 | 0.458 | -2.464 | 1.113 |
| School Fixed Effects |  |  |  |  |  |  | Removed for presentation |  |  |  |  |  |
| Constant | 29.688 | 1.841 | 16.128 | <0.001 | 26.068 | 33.307 | 26.435 | 3.032 | 8.717 | <0.001 | 20.466 | 32.403 |
| N | 376 |  |  |  |  |  | 376 |  |  |  |  |  |
| r2 | 0.360 |  |  |  |  |  | 0.490 |  |  |  |  |  |
| r2 adj. | 0.360 |  |  |  |  |  | 0.340 |  |  |  |  |  |

## Table 17: Parent DSM-IV Total T-score

|  | **Model_1 std. β** | **SE** | **t** | **p** | **95%** | **CI** | **Model_2 std. β** | **SE** | **t** | **p** | **95%** | **CI** |
| --- | --- | --- | --- | --- | --- | --- | --- | --- | --- | --- | --- | --- |
| Baseline | 0.424 | 0.032 | 13.393 | <0.001 | 0.362 | 0.486 | 0.437 | 0.038 | 11.590 | <0.001 | 0.363 | 0.512 |
| Active | -0.373 | 0.719 | -0.518 | 0.605 | -1.786 | 1.041 | -0.393 | 0.774 | -0.508 | 0.612 | -1.916 | 1.130 |
| Female |  |  |  |  |  |  | -0.268 | 0.903 | -0.297 | 0.766 | -2.045 | 1.508 |
| School Fixed Effects |  |  |  |  |  |  | Removed for presentation |  |  |  |  |  |
| Constant | 31.025 | 1.906 | 16.276 | <0.001 | 27.277 | 34.774 | 27.295 | 3.126 | 8.731 | <0.001 | 21.142 | 33.448 |
| N | 376 |  |  |  |  |  | 376 |  |  |  |  |  |
| r2 | 0.330 |  |  |  |  |  | 0.460 |  |  |  |  |  |
| r2 adj. | 0.320 |  |  |  |  |  | 0.300 |  |  |  |  |  |

## Table 18: Teachers Oppositional T-score

|  | **Model_1 std. β** | **SE** | **t** | **p** | **95%** | **CI** | **Model_2 std. β** | **SE** | **t** | **p** | **95%** | **CI** |
| --- | --- | --- | --- | --- | --- | --- | --- | --- | --- | --- | --- | --- |
| Baseline | 0.546 | 0.042 | 13.093 | <0.001 | 0.464 | 0.628 | 0.610 | 0.051 | 12.070 | <0.001 | 0.511 | 0.710 |
| Active | 0.216 | 0.897 | 0.241 | 0.810 | -1.549 | 1.981 | -0.035 | 0.958 | -0.037 | 0.971 | -1.920 | 1.850 |
| Female |  |  |  |  |  |  | 0.008 | 1.127 | 0.007 | 0.994 | -2.210 | 2.225 |
| School Fixed Effects |  |  |  |  |  |  | Removed for presentation |  |  |  |  |  |
| Constant | 25.016 | 2.316 | 10.800 | <0.001 | 20.461 | 29.571 | 24.240 | 4.282 | 5.661 | <0.001 | 15.812 | 32.667 |
| N | 376 |  |  |  |  |  | 376 |  |  |  |  |  |
| r2 | 0.320 |  |  |  |  |  | 0.460 |  |  |  |  |  |
| r2 adj. | 0.310 |  |  |  |  |  | 0.300 |  |  |  |  |  |

## Table 19: Teachers Cognitive Problems/Inattention T-score

|  | **Model_1 std. β** | **SE** | **t** | **p** | **95%** | **CI** | **Model_2 std. β** | **SE** | **t** | **p** | **95%** | **CI** |
| --- | --- | --- | --- | --- | --- | --- | --- | --- | --- | --- | --- | --- |
| Baseline | 0.537 | 0.050 | 10.631 | <0.001 | 0.437 | 0.636 | 0.515 | 0.058 | 8.875 | <0.001 | 0.401 | 0.629 |
| Active | 1.408 | 0.709 | 1.986 | 0.048 | 0.014 | 2.802 | 1.271 | 0.729 | 1.742 | 0.083 | -0.165 | 2.707 |
| Female |  |  |  |  |  |  | 3.515 | 0.872 | 4.033 | <0.001 | 1.800 | 5.231 |
| School Fixed Effects |  |  |  |  |  |  | Removed for presentation |  |  |  |  |  |
| Constant | 27.581 | 3.401 | 8.110 | <0.001 | 20.894 | 34.268 | 29.394 | 4.554 | 6.454 | <0.001 | 20.431 | 38.358 |
| N | 376 |  |  |  |  |  | 376 |  |  |  |  |  |
| r2 | 0.240 |  |  |  |  |  | 0.450 |  |  |  |  |  |
| r2 adj. | 0.240 |  |  |  |  |  | 0.280 |  |  |  |  |  |

## Table 20: Teachers Hyperactivity T-score

|  | **Model_1 std. β** | **SE** | **t** | **p** | **95%** | **CI** | **Model_2 std. β** | **SE** | **t** | **p** | **95%** | **CI** |
| --- | --- | --- | --- | --- | --- | --- | --- | --- | --- | --- | --- | --- |
| Baseline | 0.530 | 0.037 | 14.147 | <0.001 | 0.456 | 0.603 | 0.567 | 0.045 | 12.689 | <0.001 | 0.479 | 0.655 |
| Active | -0.239 | 0.717 | -0.333 | 0.739 | -1.650 | 1.171 | -0.144 | 0.788 | -0.182 | 0.856 | -1.694 | 1.407 |
| Female |  |  |  |  |  |  | 0.599 | 0.915 | 0.654 | 0.514 | -1.203 | 2.400 |
| School Fixed Effects |  |  |  |  |  |  | Removed for presentation |  |  |  |  |  |
| Constant | 24.789 | 2.133 | 11.624 | <0.001 | 20.596 | 28.983 | 21.857 | 3.475 | 6.290 | <0.001 | 15.018 | 28.696 |
| N | 376 |  |  |  |  |  | 376 |  |  |  |  |  |
| r2 | 0.350 |  |  |  |  |  | 0.460 |  |  |  |  |  |
| r2 adj. | 0.350 |  |  |  |  |  | 0.300 |  |  |  |  |  |

## Table 21: Teachers Anxious/Shy T-score

|  | **Model_1 std. β** | **SE** | **t** | **p** | **95%** | **CI** | **Model_2 std. β** | **SE** | **t** | **p** | **95%** | **CI** |
| --- | --- | --- | --- | --- | --- | --- | --- | --- | --- | --- | --- | --- |
| Baseline | 0.444 | 0.042 | 10.458 | <0.001 | 0.361 | 0.528 | 0.454 | 0.051 | 8.969 | <0.001 | 0.354 | 0.554 |
| Active | -3.041 | 0.932 | -3.263 | 0.001 | -4.873 | -1.208 | -3.703 | 0.978 | -3.785 | <0.001 | -5.629 | -1.778 |
| Female |  |  |  |  |  |  | 3.800 | 1.141 | 3.330 | 0.001 | 1.554 | 6.046 |
| School Fixed Effects |  |  |  |  |  |  | Removed for presentation |  |  |  |  |  |
| Constant | 32.171 | 2.579 | 12.473 | <0.001 | 27.100 | 37.243 | 31.865 | 4.412 | 7.223 | <0.001 | 23.182 | 40.548 |
| N | 376 |  |  |  |  |  | 376 |  |  |  |  |  |
| r2 | 0.240 |  |  |  |  |  | 0.420 |  |  |  |  |  |
| r2 adj. | 0.240 |  |  |  |  |  | 0.250 |  |  |  |  |  |

## Table 22: Teachers Perfectionism T-score

|  | **Model_1 std. β** | **SE** | **t** | **p** | **95%** | **CI** | **Model_2 std. β** | **SE** | **t** | **p** | **95%** | **CI** |
| --- | --- | --- | --- | --- | --- | --- | --- | --- | --- | --- | --- | --- |
| Baseline | 0.631 | 0.043 | 14.533 | <0.001 | 0.546 | 0.717 | 0.637 | 0.046 | 13.744 | <0.001 | 0.546 | 0.728 |
| Active | -1.167 | 0.566 | -2.062 | 0.040 | -2.281 | -0.054 | -1.642 | 0.557 | -2.947 | 0.003 | -2.739 | -0.545 |
| Female |  |  |  |  |  |  | 0.261 | 0.655 | 0.398 | 0.691 | -1.030 | 1.551 |
| School Fixed Effects |  |  |  |  |  |  | Removed for presentation |  |  |  |  |  |
| Constant | 18.608 | 2.075 | 8.969 | <0.001 | 14.528 | 22.687 | 16.099 | 2.729 | 5.899 | <0.001 | 10.728 | 21.471 |
| N | 376 |  |  |  |  |  | 376 |  |  |  |  |  |
| r2 | 0.370 |  |  |  |  |  | 0.580 |  |  |  |  |  |
| r2 adj. | 0.370 |  |  |  |  |  | 0.450 |  |  |  |  |  |

## Table 23: Teachers Social Problems T-score

|  | **Model_1 std. β** | **SE** | **t** | **p** | **95%** | **CI** | **Model_2 std. β** | **SE** | **t** | **p** | **95%** | **CI** |
| --- | --- | --- | --- | --- | --- | --- | --- | --- | --- | --- | --- | --- |
| Baseline | 0.570 | 0.041 | 13.860 | <0.001 | 0.489 | 0.651 | 0.578 | 0.046 | 12.692 | <0.001 | 0.488 | 0.668 |
| Active | -1.094 | 0.854 | -1.281 | 0.201 | -2.774 | 0.586 | -1.858 | 0.882 | -2.106 | 0.036 | -3.594 | -0.122 |
| Female |  |  |  |  |  |  | 2.530 | 1.029 | 2.460 | 0.014 | 0.506 | 4.555 |
| School Fixed Effects |  |  |  |  |  |  | Removed for presentation |  |  |  |  |  |
| Constant | 23.307 | 2.279 | 10.228 | <0.001 | 18.827 | 27.788 | 27.422 | 3.627 | 7.560 | <0.001 | 20.283 | 34.561 |
| N | 376 |  |  |  |  |  | 376 |  |  |  |  |  |
| r2 | 0.340 |  |  |  |  |  | 0.520 |  |  |  |  |  |
| r2 adj. | 0.340 |  |  |  |  |  | 0.370 |  |  |  |  |  |

## Table 24: Teachers ADHD Index T-score

|  | **Model_1 std. β** | **SE** | **t** | **p** | **95%** | **CI** | **Model_2 std. β** | **SE** | **t** | **p** | **95%** | **CI** |
| --- | --- | --- | --- | --- | --- | --- | --- | --- | --- | --- | --- | --- |
| Baseline | 0.529 | 0.044 | 12.053 | <0.001 | 0.443 | 0.615 | 0.574 | 0.053 | 10.817 | <0.001 | 0.470 | 0.679 |
| Active | -0.853 | 0.800 | -1.066 | 0.287 | -2.426 | 0.720 | -0.717 | 0.854 | -0.839 | 0.402 | -2.397 | 0.964 |
| Female |  |  |  |  |  |  | 1.436 | 0.996 | 1.442 | 0.150 | -0.524 | 3.396 |
| School Fixed Effects |  |  |  |  |  |  | Removed for presentation |  |  |  |  |  |
| Constant | 26.476 | 2.670 | 9.916 | <0.001 | 21.225 | 31.726 | 21.777 | 4.146 | 5.252 | <0.001 | 13.617 | 29.937 |
| N | 376 |  |  |  |  |  | 376 |  |  |  |  |  |
| r2 | 0.280 |  |  |  |  |  | 0.440 |  |  |  |  |  |
| r2 adj. | 0.280 |  |  |  |  |  | 0.270 |  |  |  |  |  |

## Table 25: Teachers Global Index: Restless Impulsive T-score

|  | **Model_1 std. β** | **SE** | **t** | **p** | **95%** | **CI** | **Model_2 std. β** | **SE** | **t** | **p** | **95%** | **CI** |
| --- | --- | --- | --- | --- | --- | --- | --- | --- | --- | --- | --- | --- |
| Baseline | 0.521 | 0.042 | 12.371 | <0.001 | 0.438 | 0.604 | 0.574 | 0.050 | 11.367 | <0.001 | 0.475 | 0.673 |
| Active | -0.250 | 0.788 | -0.318 | 0.751 | -1.800 | 1.299 | -0.026 | 0.850 | -0.031 | 0.976 | -1.699 | 1.646 |
| Female |  |  |  |  |  |  | 0.990 | 0.986 | 1.004 | 0.316 | -0.950 | 2.931 |
| School Fixed Effects |  |  |  |  |  |  | Removed for presentation |  |  |  |  |  |
| Constant | 26.933 | 2.560 | 10.522 | <0.001 | 21.900 | 31.966 | 21.967 | 4.004 | 5.486 | <0.001 | 14.086 | 29.847 |
| N | 376 |  |  |  |  |  | 376 |  |  |  |  |  |
| r2 | 0.300 |  |  |  |  |  | 0.430 |  |  |  |  |  |
| r2 adj. | 0.290 |  |  |  |  |  | 0.260 |  |  |  |  |  |

## Table 26: Teachers Global Index Emotional Lability T-score

|  | **Model_1 std. β** | **SE** | **t** | **p** | **95%** | **CI** | **Model_2 std. β** | **SE** | **t** | **p** | **95%** | **CI** |
| --- | --- | --- | --- | --- | --- | --- | --- | --- | --- | --- | --- | --- |
| Baseline | 0.532 | 0.040 | 13.223 | <0.001 | 0.453 | 0.611 | 0.572 | 0.048 | 11.980 | <0.001 | 0.478 | 0.666 |
| Active | -1.633 | 0.858 | -1.902 | 0.058 | -3.320 | 0.055 | -1.929 | 0.905 | -2.133 | 0.034 | -3.710 | -0.149 |
| Female |  |  |  |  |  |  | 1.076 | 1.066 | 1.010 | 0.313 | -1.021 | 3.174 |
| School Fixed Effects |  |  |  |  |  |  | Removed for presentation |  |  |  |  |  |
| Constant | 25.460 | 2.254 | 11.295 | <0.001 | 21.028 | 29.893 | 21.481 | 3.874 | 5.545 | <0.001 | 13.856 | 29.106 |
| N | 376 |  |  |  |  |  | 376 |  |  |  |  |  |
| r2 | 0.330 |  |  |  |  |  | 0.490 |  |  |  |  |  |
| r2 adj. | 0.330 |  |  |  |  |  | 0.330 |  |  |  |  |  |

## Table 27: Teachers Conners Global Index T-score

|  | **Model_1 std. β** | **SE** | **t** | **p** | **95%** | **CI** | **Model_2 std. β** | **SE** | **t** | **p** | **95%** | **CI** |
| --- | --- | --- | --- | --- | --- | --- | --- | --- | --- | --- | --- | --- |
| Baseline | 0.551 | 0.040 | 13.662 | <0.001 | 0.472 | 0.631 | 0.614 | 0.049 | 12.655 | <0.001 | 0.518 | 0.709 |
| Active | -1.208 | 0.787 | -1.536 | 0.125 | -2.755 | 0.339 | -1.137 | 0.851 | -1.335 | 0.183 | -2.813 | 0.539 |
| Female |  |  |  |  |  |  | 1.257 | 0.990 | 1.269 | 0.206 | -0.693 | 3.206 |
| School Fixed Effects |  |  |  |  |  |  | Removed for presentation |  |  |  |  |  |
| Constant | 25.425 | 2.437 | 10.433 | <0.001 | 20.633 | 30.217 | 19.335 | 3.938 | 4.910 | <0.001 | 11.585 | 27.085 |
| N | 376 |  |  |  |  |  | 376 |  |  |  |  |  |
| r2 | 0.340 |  |  |  |  |  | 0.470 |  |  |  |  |  |
| r2 adj. | 0.340 |  |  |  |  |  | 0.310 |  |  |  |  |  |

## Table 28: Teachers DSM-IV Symptoms Subscales Inattentive T-score

|  | **Model_1 std. β** | **SE** | **t** | **p** | **95%** | **CI** | **Model_2 std. β** | **SE** | **t** | **p** | **95%** | **CI** |
| --- | --- | --- | --- | --- | --- | --- | --- | --- | --- | --- | --- | --- |
| Baseline | 0.530 | 0.045 | 11.847 | <0.001 | 0.442 | 0.618 | 0.564 | 0.054 | 10.493 | <0.001 | 0.458 | 0.670 |
| Active | -0.313 | 0.737 | -0.425 | 0.671 | -1.762 | 1.136 | -0.354 | 0.767 | -0.462 | 0.645 | -1.864 | 1.156 |
| Female |  |  |  |  |  |  | 2.860 | 0.904 | 3.164 | 0.002 | 1.081 | 4.639 |
| School Fixed Effects |  |  |  |  |  |  | Removed for presentation |  |  |  |  |  |
| Constant | 26.622 | 2.833 | 9.397 | <0.001 | 21.051 | 32.193 | 21.564 | 4.123 | 5.231 | <0.001 | 13.450 | 29.678 |
| N | 376 |  |  |  |  |  | 376 |  |  |  |  |  |
| r2 | 0.270 |  |  |  |  |  | 0.460 |  |  |  |  |  |
| r2 adj. | 0.270 |  |  |  |  |  | 0.300 |  |  |  |  |  |

## Table 29: Teachers DSM-IV Symptoms Subscales Hyperactivite-Impulsive T-score

|  | **Model_1 std. β** | **SE** | **t** | **p** | **95%** | **CI** | **Model_2 std. β** | **SE** | **t** | **p** | **95%** | **CI** |
| --- | --- | --- | --- | --- | --- | --- | --- | --- | --- | --- | --- | --- |
| Baseline | 0.524 | 0.040 | 13.233 | <0.001 | 0.446 | 0.602 | 0.567 | 0.046 | 12.406 | <0.001 | 0.477 | 0.656 |
| Active | -1.234 | 0.774 | -1.595 | 0.112 | -2.755 | 0.287 | -1.248 | 0.816 | -1.530 | 0.127 | -2.854 | 0.358 |
| Female |  |  |  |  |  |  | 1.396 | 0.951 | 1.469 | 0.143 | -0.475 | 3.268 |
| School Fixed Effects |  |  |  |  |  |  | Removed for presentation |  |  |  |  |  |
| Constant | 25.059 | 2.191 | 11.438 | <0.001 | 20.751 | 29.367 | 21.051 | 3.573 | 5.891 | <0.001 | 14.018 | 28.084 |
| N | 376 |  |  |  |  |  | 376 |  |  |  |  |  |
| r2 | 0.330 |  |  |  |  |  | 0.480 |  |  |  |  |  |
| r2 adj. | 0.320 |  |  |  |  |  | 0.330 |  |  |  |  |  |

## Table 30: Teachers DSM-IV Symptoms Subscale Total T-score

|  | **Model_1 std. β** | **SE** | **t** | **p** | **95%** | **CI** | **Model_2 std. β** | **SE** | **t** | **p** | **95%** | **CI** |
| --- | --- | --- | --- | --- | --- | --- | --- | --- | --- | --- | --- | --- |
| Baseline | 0.519 | 0.043 | 12.019 | <0.001 | 0.434 | 0.604 | 0.556 | 0.052 | 10.787 | <0.001 | 0.454 | 0.657 |
| Active | -0.549 | 0.741 | -0.741 | 0.459 | -2.007 | 0.908 | -0.542 | 0.784 | -0.691 | 0.490 | -2.085 | 1.002 |
| Female |  |  |  |  |  |  | 2.687 | 0.918 | 2.929 | 0.004 | 0.881 | 4.493 |
| School Fixed Effects |  |  |  |  |  |  | Removed for presentation |  |  |  |  |  |
| Constant | 27.201 | 2.609 | 10.427 | <0.001 | 22.072 | 32.331 | 22.329 | 3.969 | 5.625 | <0.001 | 14.516 | 30.141 |
| N | 376 |  |  |  |  |  | 376 |  |  |  |  |  |
| r2 | 0.280 |  |  |  |  |  | 0.440 |  |  |  |  |  |
| r2 adj. | 0.280 |  |  |  |  |  | 0.280 |  |  |  |  |  |
